# Exercise alters the circadian rhythm of REV-ERB-α and downregulates autophagy-related genes in peripheral and central tissues

**DOI:** 10.1038/s41598-022-24277-4

**Published:** 2022-11-21

**Authors:** Alisson L. da Rocha, Ana P. Pinto, Bruno L. S. Bedo, Gustavo P. Morais, Luciana C. Oliveira, Ruither O. G. Carolino, Jose R. Pauli, Fernando M. Simabuco, Leandro P. de Moura, Eduardo R. Ropelle, Dennys E. Cintra, Donato A. Rivas, Adelino S. R. da Silva

**Affiliations:** 1 Postgraduate Program in Rehabilitation and Functional Performance, Ribeirão Preto Medical School, University of São Paulo (USP), Avenida Bandeirantes, 3900, Monte Alegre, Ribeirão Preto, São Paulo 14040-907 Brazil; 2 School of Physical Education and Sport of Ribeirão Preto, University of São Paulo (USP), Ribeirão Preto, São Paulo Brazil; 3 Department of Sport, School of Physical Education and Sport, University of São Paulo, São Paulo, Brazil; 4 Laboratory of Molecular Biology of Exercise (LaBMEx), School of Applied Sciences, University of Campinas (UNICAMP), Limeira, São Paulo Brazil; 5 Nutrition, Exercise Physiology and Sarcopenia Laboratory, Tufts University, Boston, MA USA

**Keywords:** Metabolism, Autophagy, Circadian rhythms

## Abstract

The transcriptional repressor REV-ERB-α, encoded by Nuclear Receptor Subfamily 1 Group D Member 1 (*Nr1d1*), has been considered to play an essential role in the skeletal muscle oxidative capacity adaptation and muscle mass control. Also, this molecule regulates autophagy via the repression of autophagy-related genes both in skeletal muscle and brain regions. Classically, training programs based on endurance or strength characteristics enhance skeletal muscle mass content and/or oxidative capacity, leading to autophagy activation in several tissues. Thus, it seems that REV-ERB-α regulates similar responses induced by exercise. However, how this molecule responds to different exercise models/intensities in different tissues is still unclear. Therefore, the main aim was to characterize the responses of REV-ERB-α and autophagy-related genes to different exercise protocols (endurance/interval run/strength) in distinct tissues (gastrocnemius, soleus and hippocampus). Since REV-ERB-α presents a circadian rhythm, the analyses were performed in a time-course manner. The endurance and strength groups attenuated REV-ERB-α transcriptional response during the time course in gastrocnemius and soleus. Conversely, the interval group enhanced the *Nr1d1* expression in the hippocampus. All protocols downregulated the REV-ERB-α protein levels in gastrocnemius following the exercise session with concomitant nuclear exclusion. The major autophagy-related genes presented downregulation after the exercise session in all analyzed tissues. Altogether, these results highlight that REV-ERB-α is extremely sensitive to physical exercise stimuli, including different models and intensities in skeletal muscle and the hippocampus.

## Introduction

Physical exercise stimulates several responses and adaptations in the human body, ranging from the prevention/treatment of diseases such as diabetes, hypertension, and obesity^1^ to performance improvements for amateur/elite athletes^2^. Skeletal muscle has an exceptional capacity for adaptation and remodeling in response to physical exercise, culminating in several parameter changes, such as a shift of contractile fibers profile, mitochondrial density, metabolic regulation, intracellular signaling pathways modulation and transcriptional activity^3^. These adaptations are specific to the training model. Although there are several, the classical literature classifies the training models as endurance training, which induces increased aerobic capacity, mitochondrial biogenesis, and muscle fibers with oxidative characteristics, and strength training, which causes increased protein synthesis, muscle hypertrophy and increased maximal contractile strength^3^.

In 2013, Woldt et al.^4^ highlighted the central role of an emerging protein, the nuclear receptor REV-ERB-α, in the oxidative capacity of skeletal muscle. REV-ERB-α is encoded by the nuclear receptor subfamily 1 group D member 1 gene (*Nr1d1*) and is a potent transcriptional repressor that can control the expression pattern of several genes^5^. Previous studies have described its function related to circadian rhythm^6^ and regulating hepatic metabolism and brown adipose tissue^7^. However, Woldt and coworkers^4^ revealed that REV-ERB-α plays a central role in the activation of the AMP-activated protein kinase (AMPK)—Sirtuin 1 (SIRT1)—Peroxisome proliferator-activated receptor-gamma coactivator 1-α (PGC-1α) signaling pathway, predominantly in oxidative muscles. When pharmacologically activated, REV-ERB-α is demonstrated to increase physical performance, mitochondrial content, and function.

Mayeuf-Louchart et al.^8^ displayed that REV-ERB-α has a vital role in the control of skeletal muscle mass. The authors showed that this transcriptional repressor regulates the expression of several atrophy-related genes, such as Muscle RING-finger protein-1(*Murf1*) and F-box only protein 32 (*Atrogin-1*). Global REV-ERB-α knockout mice exhibited upregulation of these atrophy-related genes, which are associated with decreased skeletal muscle mass and fiber size. Conversely, REV-ERB-α overexpression in C2C12 cells showed opposite characteristics, with increased fiber size and downregulation of atrophy-related genes. Interestingly, these positive adaptations are achieved by strength training programs. These discoveries (4, 8) strongly suggest the REV-ERB-α as an “exercise molecule”, which could be responsive to physical exercise and perform roles in skeletal muscle adaptations in this context.

The REV-ERB-α has a direct repressive regulatory role in autophagic gene transcription of skeletal muscle^4^. Huang et al.^9^ showed that REV-ERB-α also controls the rhythm and intensity of autophagy-related genes in the brain. Physical exercise activates the autophagic mechanism in cells, as well as in peripheral and central (central nervous system/CNS) tissues^10–12^. In fact, for general responses to exercise, the hippocampus is the most impacted brain region^13^. Schwalm et al.^14^ evidenced that the autophagy process depends on the exercise intensity. However, several mechanisms mediate exercise-induced autophagy and can respond precisely to specific exercise models and in different tissues^11,12,15^. Also, it is currently unknown if REV-ERB-α regulates the autophagy pathway in response to physical exercise. Unraveling this molecule's responses to different exercise models and intensities associated with the autophagy pathway will provide crucial information to support exercise programs' organization to specific goals.

To characterize the responses of REV-ERB-α to physical exercise, we utilized three different exercise protocols (endurance, interval run and strength). The interval model provided information on whether REV-ERB-α responses are sensitive to different exercise intensities. Firstly, we evaluated the time-course response of *Rev-erb-α* in specific tissues (gastrocnemius, soleus, and hippocampus) after different exercise protocols. Subsequently, we assessed the time-course response and subcellular localization of REV-ERB-α and inflammatory genes expression after these exercise protocols in gastrocnemius samples. Further, after these protocols, we evaluated the time-course response of crucial autophagy genes in different tissues. Finally, we correlated the REV-ERB-α responses with autophagy-related gene expression after different exercise protocols in skeletal muscle. We hypothesized that REV-ERB-α would be upregulated (mRNA and protein) in response to exercise with concomitant upregulation of the autophagy genes.

## Results

**Figure 1:**
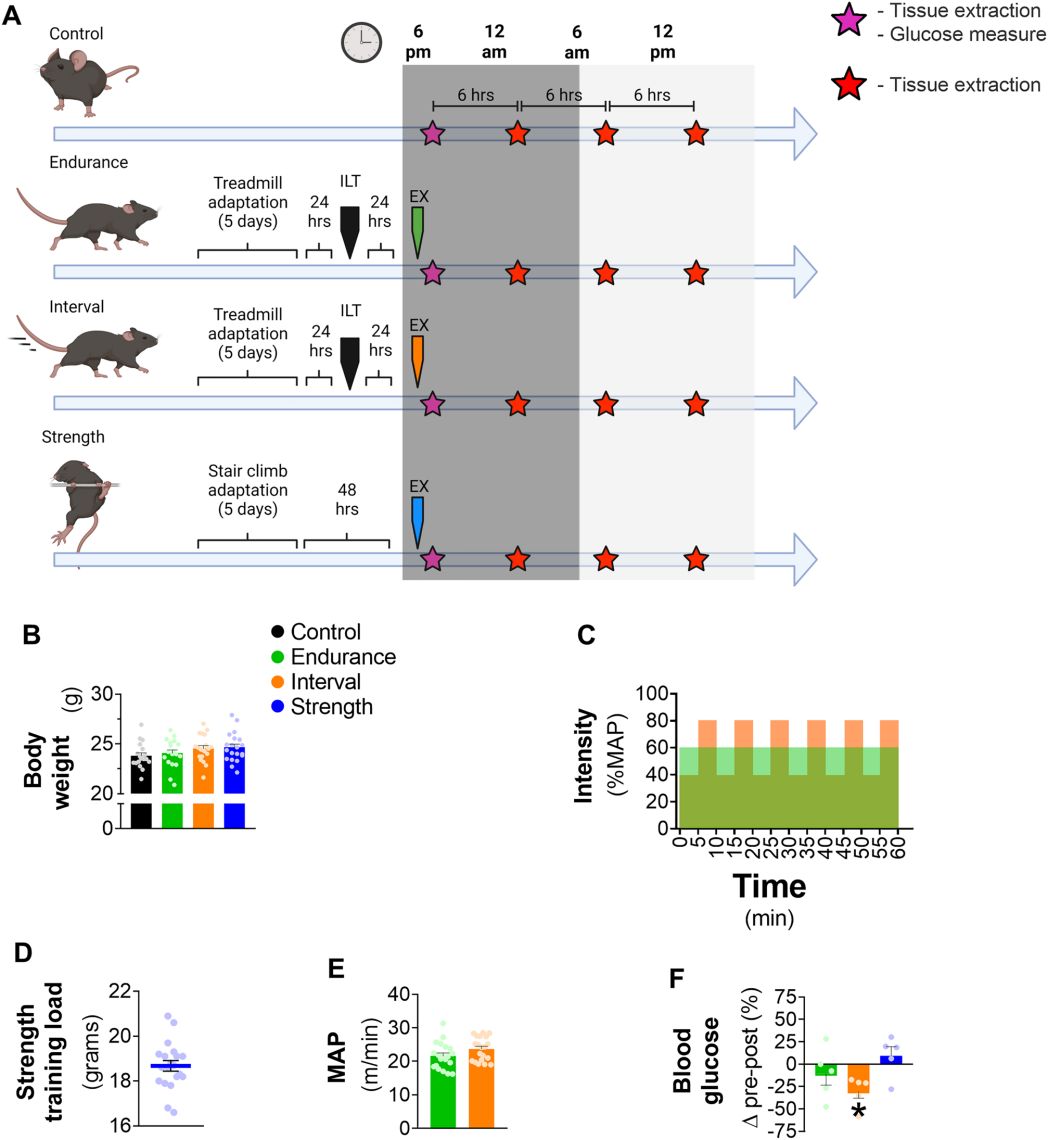
Schematic presentation of the experimental procedures (**A**)—created with BioRender.com; mean ± SEM of body weight (**B**); schematic presentation of the endurance and interval exercises protocol (**C**); mean ± SEM of strength training load (**D**). maximum aerobic power (**E**; MAP), and pre-post exercise variation of the blood glucose (**F**); Data correspond to n = 18–20 mice for (**B**–**E**), and n = 5 for F; *p < 0.05 pre *vs.* post-exercise session. *MAP* maximum aerobic power, *ILT* incremental load test, *EX* exercise session, *Control* sedentary mice, *Endurance* mice submitted to continuous aerobic exercise session, *Interval* mice submitted to interval aerobic exercise session, *Strength* mice submitted to resistance exercise session. Created with GraphPad Prism v.8.0.1 (http://www.graphpad.com).

The schematic presentation of the experimental procedures is presented in Fig. 1A. Briefly, the mice were randomly assigned to four groups: Control (CT), Endurance (END), Interval (INT) and Strength (STR). The mice at the END and INT groups were adapted to the treadmill for 5 consecutive days, rested for 24 h, performed the Incremental Load Test (ILT) to achieve Maximum Aerobic Power (MAP) values, rested for another 24 h and, finally, were submitted to the acute exercise session (starting at 6 p.m.). The STR group was adapted to stair climbing for 5 consecutive days, rested for 48 h and, finally, the mice were submitted to the acute strength exercise session (starting at 6 p.m.). The glucose levels were measured immediately after the exercise session (END, INT and STR). Tissue extraction occurred in a time-course manner (every 6 h, initiating immediately after the end of the exercise session).

### Only aerobic interval training reduced the blood glucose after the exercise session

The groups did not present differences in body weight (Fig. 1B). Figure 1C shows a schematic presentation of the END and INT exercise parameters (intensity × duration). The loads utilized in the STR exercise protocol are exhibited in Fig. 1D. The END and INT groups did not differ in the velocity corresponding to MAP (Fig. 1E). Only the INT group presented a reduction in blood glucose after the acute exercise protocol compared to basal levels (Fig. 1F).

### Endurance and strength exercise downregulated *Nr1d1* expression in gastrocnemius and soleus

**Figure 2:**
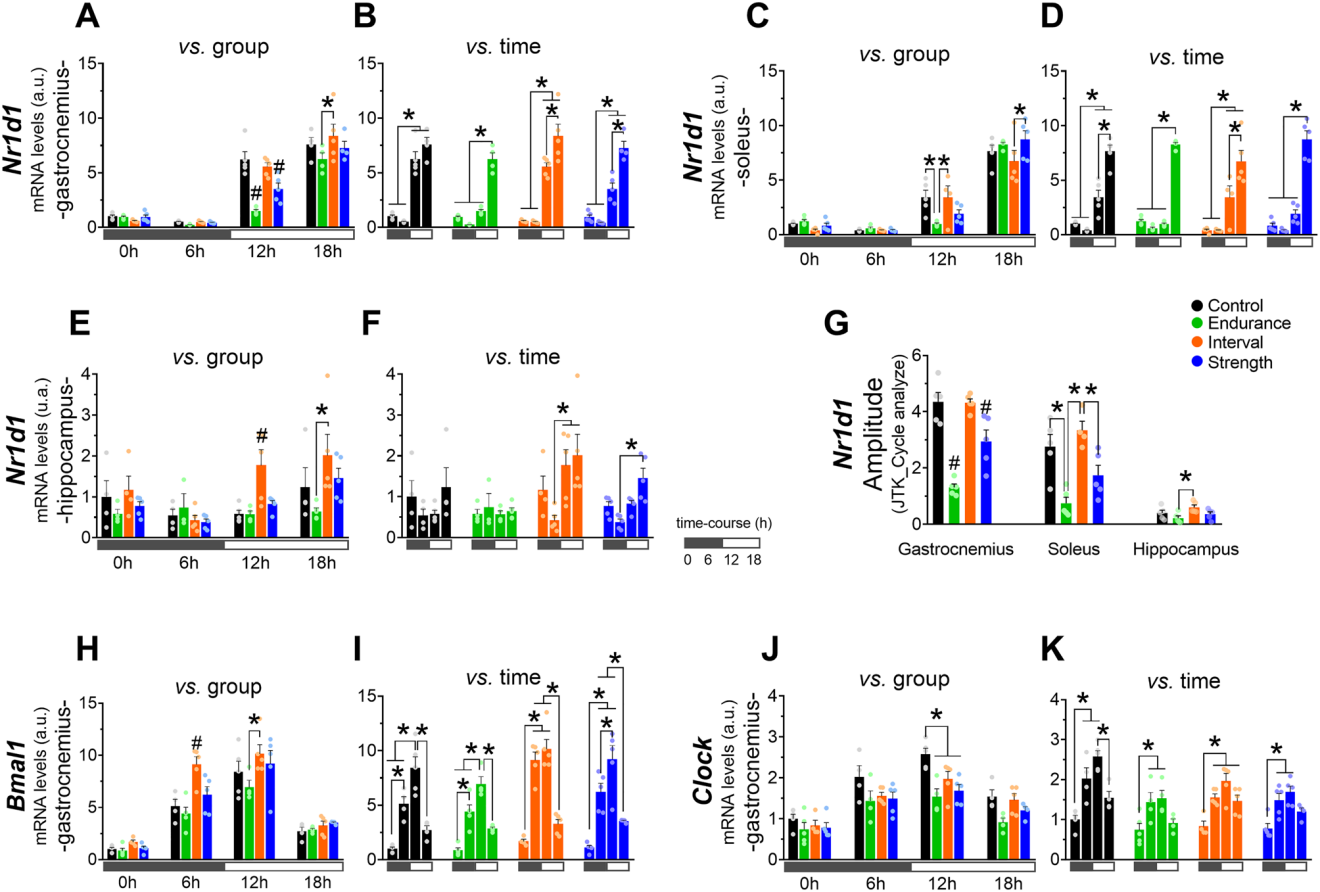
mRNA levels of *Nr1d1* in gastrocnemius (**A** and **B**; compared between groups and time, respectively), soleus (**C** and **D**; compared between groups and time, respectively), and hippocampus (**E** and **F**; compared between groups and time, respectively). The amplitude of the *Nr1d1* circadian rhythm utilizing the R statistical package v.4.2.1 (https://www.r-project.org/) and JTK_Cycle algorithm in gastrocnemius, soleus and hippocampus (**G**). mRNA levels of *Bmal1* (**H** and **I**; compared between groups and time, respectively) and *Clock* (**J** and **K**; compared between groups and time, respectively) in gastrocnemius. Data correspond to the mean ± SEM of 3–5 mice; a.u.: arbitrary units; *p < 0.05 vs. another experimental group. ^#^p < 0.05 vs. all the other experimental groups for the same time-point. Created with GraphPad Prism v.8.0.1 (http://www.graphpad.com).

In gastrocnemius samples**,** the END and STR groups showed lower levels of *Nr1d1* mRNA at 12 h compared to CT and INT. At 18 h post-exercise, the END group showed reduced levels compared to INT (Fig. 2A), while the CT, INT, and STR groups showed a circadian oscillatory pattern characterized by higher levels of *Nr1d1* mRNA at 12 and 18 h compared to 0 and 6 h. The END group showed higher levels only at 18 h compared to 0, 6, and 12 h. The INT and STR groups also showed higher levels of *Nr1d1* mRNA at 18 h compared to 12 h (Fig. 2B).

In soleus samples, the END group exhibited lower levels of *Nr1d1* mRNA at 12 h compared to CT and INT groups. The STR group presented higher mRNA levels at 18 h than INT (Fig. 2C). The CT and INT groups showed higher levels of *Nr1d1* mRNA at 12 and 18 h compared to 0 and 6 h. Both groups also presented higher levels at 18 h compared to 12 h. The END and STR groups showed higher levels of *Nr1d1* mRNA at 18 h compared to 0, 6, and 12 h (Fig. 2D).

### Interval exercise upregulated *Nr1d1* expression in the hippocampus

In hippocampus samples, the INT group exhibited higher levels of *Nr1d1* mRNA compared to all the other experimental groups at 12 h. At 18 h, the INT group showed higher levels only when compared to the END group (Fig. 2E). The INT group showed higher levels of *Nr1d1* mRNA at 12 and 18 h than at 6 h. The STR group only showed higher mRNA levels at 18 h compared to 6 h (Fig. 2F).

### Endurance and strength exercise attenuates the amplitude of the *Nr1d1* circadian rhythm in gastrocnemius and soleus, while interval exercise increases it in the hippocampus

The END and STR groups showed lower values for circadian rhythm amplitude analyzed in the JTK_cycle algorithm compared to CT and INT in gastrocnemius samples. Also, the END group showed lower values when compared to STR. In soleus samples, the END group presented lower values when compared to CT and INT, while STR showed reduced values only when compared to INT. In the hippocampus, the INT group exhibited a higher amplitude for Nr1d1 compared to the END group (Fig. 2G).

### Interval exercise increases *Bmal1* gene expression in the gastrocnemius

In gastrocnemius samples, the INT group presented higher levels of *Bmal1* mRNA compared to all other experimental groups at 6 h. At 12 h, the INT group showed higher levels only when compared to the END group (Fig. 2H). All experimental groups exhibited higher levels of *Bmal1* mRNA at 6 and 12 h compared to their respective 0 h. The CT, END and STR groups presented higher levels at 12 h compared to 6 h. The INT and STR groups showed lower levels at 18 h compared to 6 and 12 h, while CT and END showed differences only when compared to 12 h (Fig. 2I).

### All exercise models downregulated *Clock* gene expression in gastrocnemius

In gastrocnemius samples, all exercised groups showed lower levels of *Clock* mRNA when compared to CT at 12 h (Fig. 2J). The CT, END and STR groups presented higher levels of *Clock* mRNA at 6 and 12 h compared to 0 h, while the INT group showed increased levels in all other time points compared to 0 h. Only the CT group exhibited lower levels at 18 h compared to 12 h (Fig. 2K).

### Acute exercise, independently of the intensity and model, reduced the total REV-ERB-α protein content and nuclear localization in gastrocnemius

**Figure 3:**
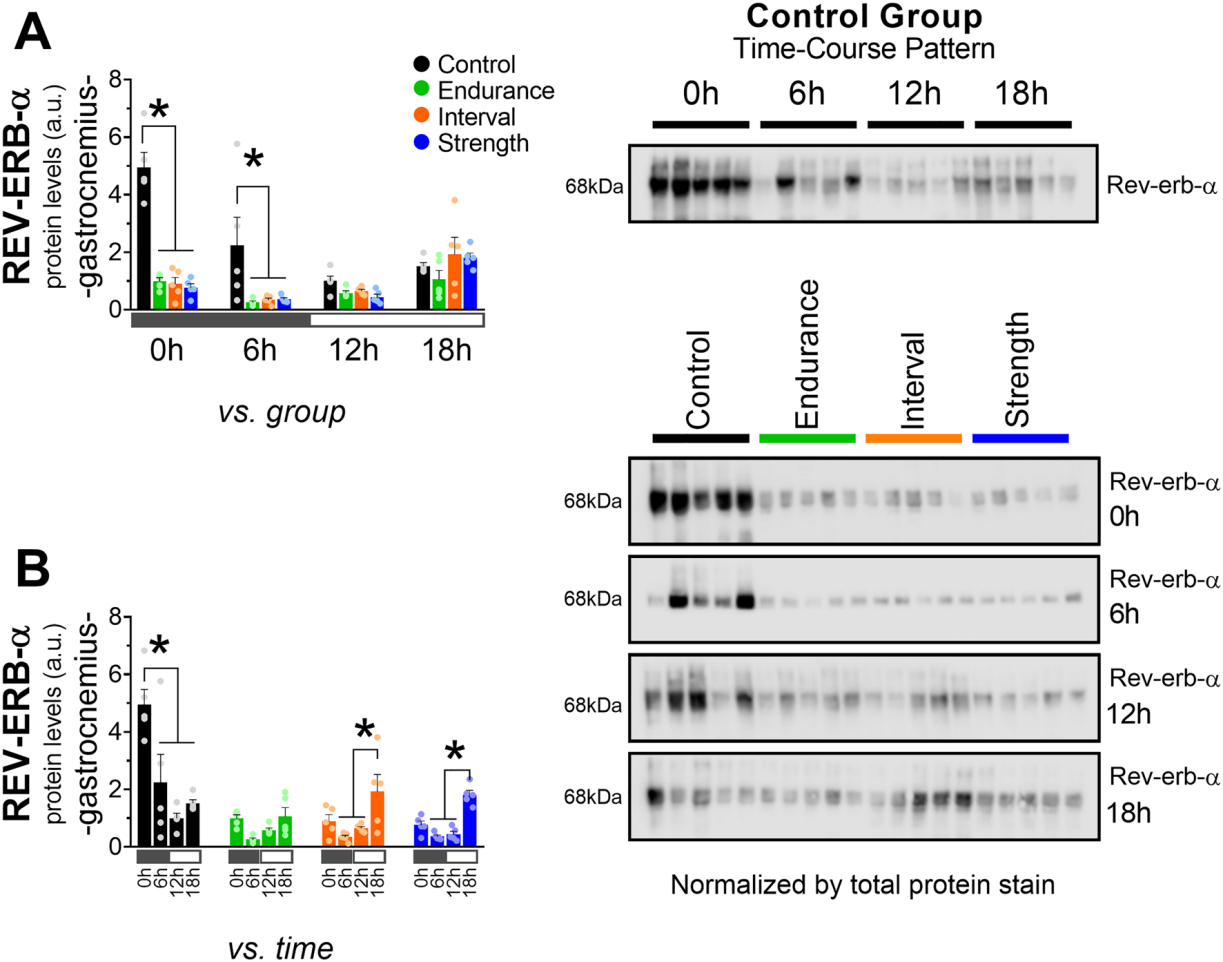
Comparison of REV-ERB-α protein levels in gastrocnemius between groups (**A**) and time (**B**). Data correspond to the mean ± SEM of 5 mice; a.u.: arbitrary units; *p < 0.05 vs. another experimental group. The blots were normalized by the total protein stain method (images are available in Supplementary File Information). Created with GraphPad Prism v.8.0.1 (http://www.graphpad.com).

**Figure 4:**
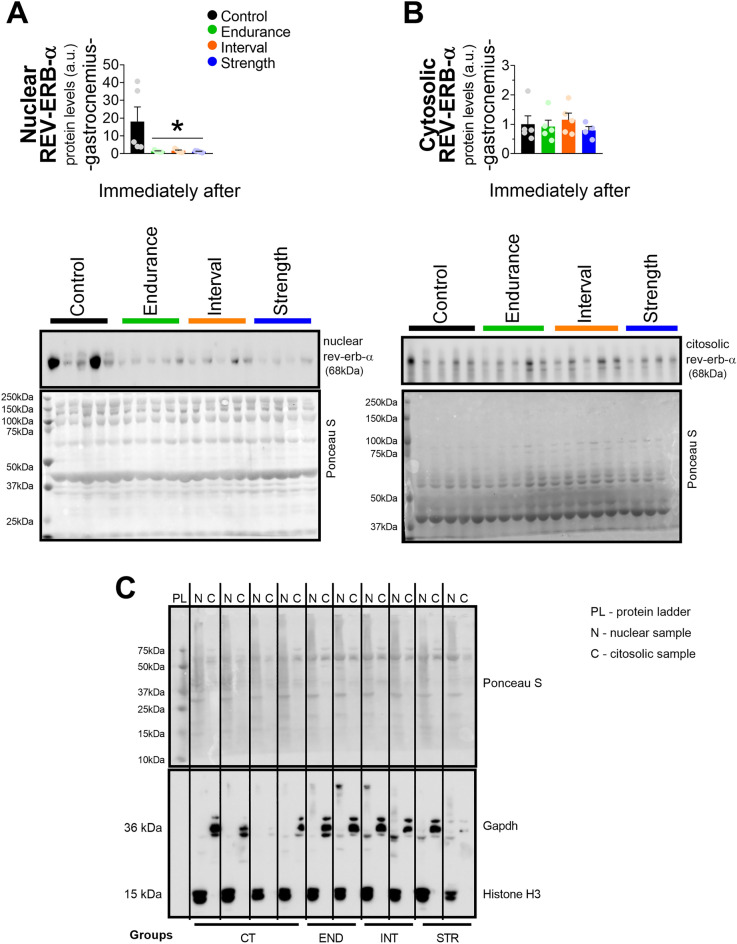
Protein levels of REV-ERB-α in nuclear (**A**) and cytosolic (**B**) fractions. The values were corrected using Ponceau S method. Data correspond to the mean ± SEM of 4–5 mice Contamination test of the subcellular fractionation (**C**); a.u.: arbitrary units; *p < 0.05 vs. control group. Created with GraphPad Prism v.8.0.1 (http://www.graphpad.com).

All exercised groups (END, INT, and STR) showed a marked reduction in REV-ERB-α protein content in gastrocnemius at 0 and 6 h (Fig. 3A). The CT group showed higher total REV-ERB-α at 0 than at 6, 12, and 18 h. Also, this group presented lower protein levels at 12 h compared to 6 h. The INT group showed higher levels at 18 h than at 12 and 6 h (Fig. 3B). The exercised groups showed lower nuclear REV-ERB-α than CT at 0 h (Fig. 4A) without alterations in the cytosolic fraction (Fig. 4B). Figure 4C shows the contamination experiment of the subcellular fractioning. The nuclear fractions showed only bands when incubated with Histone H3 antibody (a nuclear protein), and the cytosolic only showed bands when incubated with GAPDH antibody (a cytosolic protein).

### All exercised groups downregulated autophagy-related genes in all analyzed tissues and modulated inflammatory genes in gastrocnemius

**Figure 5:**
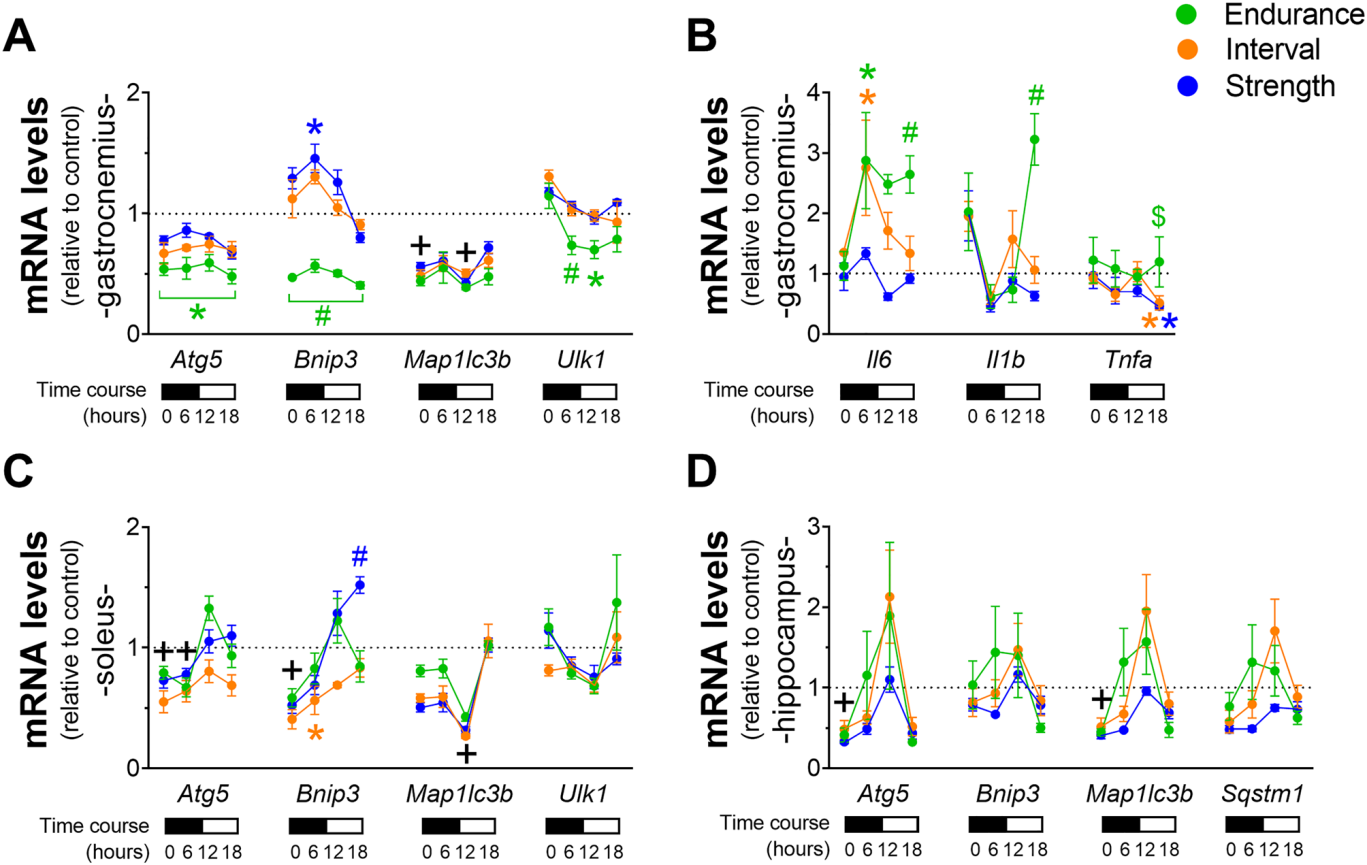
Gene expression of *Atg5, Bnip3, Map1lc3b* and *Ulk1* in gastrocnemius (**A**); *Il6*, *Il1b* and *Tnfa* in gastrocnemius (**B**); *Atg5, Bnip3, Map1lc3b* and *Ulk1* in soleus (**C**); *Atg5, Bnip3, Map1lc3b* and *Sqstm1* in the hippocampus (**D**) relative to the control group. Data correspond to the mean ± SEM of 4–5 mice; *p < 0.05 vs. control group; ^#^p < 0.05 vs. all other experimental groups. ^$^p < 0.05 vs. INT and STR. The symbol color is associated with group statistical differences. ^+^p < 0.05: all exercised groups vs. control group. Created with GraphPad Prism v.8.0.1 (http://www.graphpad.com).

In gastrocnemius, the END group showed reduced levels of *Atg5* mRNA compared to CT in all investigated time points. The END group showed lower levels of *Bnip3* compared to all experimental groups in all investigated time points. The STR group presented higher *Bnip3* levels at 6 h compared to CT. All exercised groups presented lower levels of *Map1lc3b* mRNA than CT at 0 and 12 h. The END group showed lower *Ulk1* levels than all experimental groups at 6 h. Also, this group exhibited reduced levels at 12 h compared to CT (Fig. 5A). The END and INT groups presented higher levels of *Il6* mRNA compared to CT at 6 h. At 18 h, the END group showed higher *Il6* and *Il1b* mRNA levels than all other experimental groups. All exercised groups showed an increase of around twofold (not statistically significant) of *Il1b* mRNA levels immediately after the exercise session (Fig. 5B). The INT and STR groups presented a reduction in *Tnfa* mRNA levels at 18 h compared to END and CT.

In soleus, all the exercised groups showed lower *Atg5* levels than CT at 0 and 6 h. All the exercised groups showed reduced *Bnip3* levels compared to CT at 0 h. The INT group showed lower *Bnip3* levels at 6 h compared to CT. The STR group showed higher *Bnip3* levels than the other experimental groups at 18 h. All exercise groups showed lower *Map1lc3b* levels than CT at 12 h (Fig. 5C). In the hippocampus, all exercised groups showed reduced *Atg5* and *Map1lc3b* levels compared to CT at 0 h (Fig. 5D).

### REV-ERB-α presents a moderate correlation with some autophagy-related genes in response to an acute exercise session in gastrocnemius

**Figure 6:**
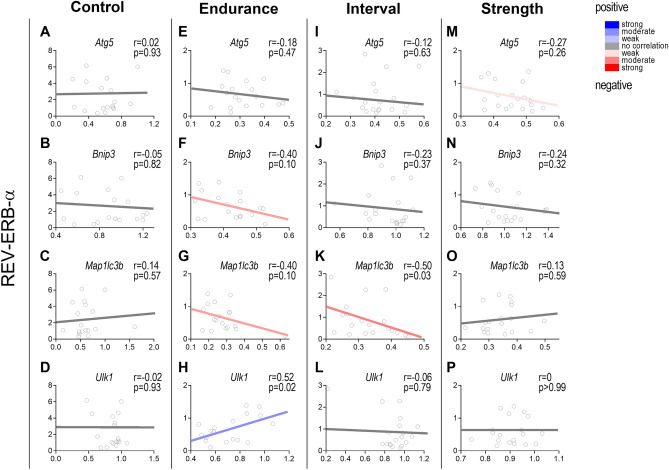
Correlations between REV-ERB-α protein levels and *Atg5, Bnip3*, *Map1lc3b,* and *Ulk1* mRNA levels for CT (**A**–**D**), END (**E**–**H**), INT (**I**–**L**) and STR (**M**–**P**) groups in gastrocnemius. Data correspond to 18–20 mice. Created with GraphPad Prism v.8.0.1 (http://www.graphpad.com).

The CT group did not show a significative correlation between REV-ERB-α and the analyzed autophagy-related genes (Fig. 6A–D). The END group did not present a correlation between REV-ERB-α and *Atg5* in gastrocnemius (Fig. 6E). However, it showed moderate negative correlations with *Bnip3* and *Map1lc3b* expressions (r = − 0.40; Fig. 6F,G, respectively). Also, for the END group, this protein showed a moderate positive correlation (r = 0.52) with *Ulk1* (Fig. 6H). The INT group did not present significant correlations between REV-ERB-α and *Atg5, Bnip3* and *Ulk1* (Fig. 6I,J,L). Nonetheless, this group showed a moderate negative correlation between REV-ERB-α and *Map1lc3b* (r = − 0.50) in gastrocnemius (Fig. 6K). For STR, the REV-ERB-α did not exhibit correlations with *Bnip3*, *Map1lc3b* and *Ulk1* in gastrocnemius (Fig. 6N–P). This group only showed a weak negative correlation for this protein with *Atg5* (r = − 0.27; Fig. 6M).

## Discussion

The main findings of the present investigation were: (a) the END and STR protocol reduced the transcriptional expression of *Rev-erb-α* 12 h post-exercise in gastrocnemius and soleus; (b) when compared to END, the INT group increased the transcriptional expression of *Rev-erb-α* 12 and 18 h post-exercise in the analyzed skeletal muscles and hippocampus; (c) all exercised groups reduced total REV-ERB-α protein levels in gastrocnemius for at least the first 6 h post-exercise; (d) all exercised groups reduced the REV-ERB-α nuclear content immediately after the exercise session in gastrocnemius; (e) overall, all exercise protocols reduced the autophagy genes expression in the analyzed skeletal muscles and hippocampus. For the first time, we reveal a differential response of REV-ERB-α to 3 different exercise models progressively over time, highlighting the nuclear exclusion in skeletal muscle. Also, we showed a moderate correlation between REV-ERB-α and autophagy-related genes responses to physical exercise, especially in aerobic exercise models.

In soleus and gastrocnemius samples, skeletal muscles with distinct characteristics and oxidative potential, endurance (END) and strength (STR) exercise models showed similar attenuation in *Rev-erb-α* expression during the circadian cycle. On the other hand, the interval aerobic exercise (INT) model showed higher *Rev-erb-α* expression during the circadian cycle (i.e., 12- and 18-h post-exercise) compared to the continuous aerobic exercise (END) in both the skeletal muscles and hippocampus. Although END and INT are exercise models thought to bring about similar physiological adaptations^16^, they induced different responses to this gene in skeletal muscle. It is essential to highlight that these two exercise models showed the same training load (i.e., 3.600 arbitrary units), which was obtained by the multiplication of intensity (% of MAP velocity) and volume (duration of the training session). Both protocols presented the same volume (60 min), showing differences in the intensity of the exercise. These results propose that the *Rev-erb-α* transcriptional response in skeletal muscle and hippocampus may be more influenced by exercise intensity than the exercise model.

Interval aerobic exercise has emerged as an alternative to increasing aerobic performance, proving as efficient as continuous aerobic training^17^. The advantage of this method versus the classical endurance protocol would be the shorter training session duration needed to increase aerobic performance^18^. Furthermore, Radak et al.^19^ suggested that the recovery time after an interval training session is faster than long-duration endurance exercise. The molecular mechanisms leading to skeletal muscle adaptations appear similar in both exercise methods, converging on the AMPK/PGC1α signaling pathway that culminates in mitochondrial biogenesis^20^. However, INT exercise recruits more fast-twitch fibers (type II) due to the higher intensity of exercise^21^. Regarding these two aerobic protocols, the INT group showed a more pronounced response of the *Rev-erb-α* in gastrocnemius muscle (composed of more IIb than IIa mixed fibers) compared to soleus (composed of oxidative I and more IIa than IIb mixed fibers). This data reinforces the theory that exercise intensity and muscle fiber composition are essential factors in *Rev-erb-α* responses to physical exercise.

REV-ERB-α presents a circadian rhythm^22^. This oscillation pattern is vital for controlling daily cycles and metabolism in various tissues, such as the hypothalamus, liver, adipose tissue, skeletal muscle, and others^23^. Dysfunction of the circadian cycle can increase the chance of developing obesity, type 2 diabetes, hyperlipidemia, increased blood pressure, and cardiovascular disease. Gabriel and Zierath^24^ reviewed the importance of physical exercise as a non-pharmacological intervention in treating metabolic disorders. Also, these authors highlighted the importance of the investigations targeting the link between physical exercise and the machinery responsible for the cyclic oscillations (i.e., clock genes) in metabolic tissues. Physical exercise can modulate the amplitude and phase of the circadian rhythm in skeletal muscle^25^, characterizing a resetting of the clock genes^24^. Indeed, we confirmed that different exercise models induced a change in the expression of central clock genes in the gastrocnemius (i.e., *Rev-erb-α*, *Bmal1* and *Clock*). Specifically, in skeletal muscle samples (gastrocnemius and soleus), we highlighted that the endurance and strength exercise attenuated the amplitude of *Rev-erb-α* circadian pattern. In the hippocampus, the interval run group exhibited higher amplitude for *Rev-erb-α* circadian rhythm compared to classical endurance exercise. This data reinforces the theory that REV-ERB-α transcriptional response may be more sensitive to exercise intensity than exercise model.

Interestingly, in the present investigation, physical exercise (regardless of the model) reduced the REV-ERB-α protein content in the gastrocnemius, abolishing its characteristic oscillation during the 18 h following the end of the exercise session. Furthermore, this phenomenon was accompanied by excluding REV-ERB-α from the cell nucleus immediately at the session end. These results reinforce the concept of exercise as a potent modulator of REV-ERB-α, an important component in the machinery of circadian rhythm in skeletal muscle. The molecular mechanisms by which physical exercise decreased REV-ERB-α protein content in skeletal muscle are unknown since this is the first time this dynamic has been demonstrated. However, it is already well described in the scientific literature that intracellular proteins, in stable situations, are in constant turnover, being degraded and resynthesized to maintain a functional protein pool^26^.

Most intracellular proteins are degraded in all tissues via the ubiquitin–proteasome (UPP) pathway^26^. Briefly, UPP consists of binding the polypeptide cofactor Ub (ubiquitin) to proteins, signaling them to be degraded by proteasomes. Pariollaud et al.^27^ identified the UPP signaling pathway linking inflammation and REV-ERB-α degradation. The authors stated that the UPP pathway is an essential regulatory system to control the function of this nuclear receptor in lung epithelial cells. Indeed, REV-ERB-α was rapidly degraded via UPP in response to the interleukin-1β (IL-1β) and tumor necrosis factor α^27^. It is known that physical exercise induces the production of several cytokines (pro and anti-inflammatory) depending on the model, intensity, and duration of the stimulus^28^. IL-1β transcriptional response in gastrocnemius was upregulated twofold for all exercise groups in the present investigation. Accordingly, we can suggest that the reduction of REV-ERB-α in skeletal muscle may occur through the UPP pathway induced by pro-inflammatory cytokines produced in response to physical exercise.

The nuclear location of REV-ERB-α may characterize another regulatory mechanism of this transcriptional repressor since its action is described in the nucleus^6^. Thus, its bioavailability in the nuclear compartment may suggest REV-ERB-α activity more accurately than its total protein content. Interestingly, in addition to reducing the total content of REV-ERB-α, the different exercise models reduced the nuclear presence of this protein without changes in the cytosolic extract. Therefore, this result shed light on some novel mechanisms responsible for lowering the REV-ERB-α content exclusively in the nucleus of skeletal muscle cells immediately at the end of the exercise session.

REV-ERB-α can be phosphorylated at serines 55 and 59 by glycogen synthase kinase-3β (GSK-3β), and this post-translational modification appears to increase this stability protein^23^. In addition to increasing REV-ERB-α stability, this phosphorylation may change its subcellular location, as some transcription factors do^29^. As proposed previously, this migration of phosphorylated REV-ERB-α from the nucleus to the cytosol may increase stability and protect its degradation via the UPP pathway. Thus, nuclear REV-ERB-α may be more susceptible to degradation when compared to its cytosolic location. Future studies should investigate this hypothesis and elucidate the mechanisms responsible for this response.

Woldt et al.^4^ demonstrated that several autophagic genes directly target REV-ERB-α action, suppressing their transcription. Nonetheless, we have not verified significant correlations between them at basal conditions. In the exercise context, most of the autophagy genes we measured showed transcriptional attenuation with concomitant reduction of REV-ERB-α. Importantly, REV-ERB-α showed a moderate negative correlation with some autophagy genes, especially for *Map1lc3b* in aerobic exercise models, which was downregulated after the exercise session in gastrocnemius. Although these correlations suggest an interaction between REV-ERB-α and some of these autophagy-related genes in skeletal muscle, it is impossible to confirm a causal relationship in these exercises context. Future studies should investigate this topic to elucidate this interaction.

Overall, the *Atg5* and *Map1lc3b* genes were downregulated in the skeletal muscle and hippocampus of all exercised groups. The proteins encoded by these genes participate in autophagosome formation and mitochondrial quality control after oxidative damage^30^. These data highlight the impact of physical exercise on metabolism, modulating autophagy in skeletal muscle and CNS. Furthermore, these observations suggest that physical exercise, regardless of the model, can modulate autophagy responses in the hippocampus, which plays a role in learning and memory^31^. Concerning the skeletal muscle, the autophagy response to physical exercise has a biphasic characteristic, highlighted by a rapid increase in autophagic flux occurring within minutes to hours, which is mediated by post-translational modifications of the proteins responsible for this pathway. Generally, after this initial stimulus, activation of the transcriptional program of autophagic genes occurs in the long term to potentiate this autophagic response^10^.

**Figure 7:**
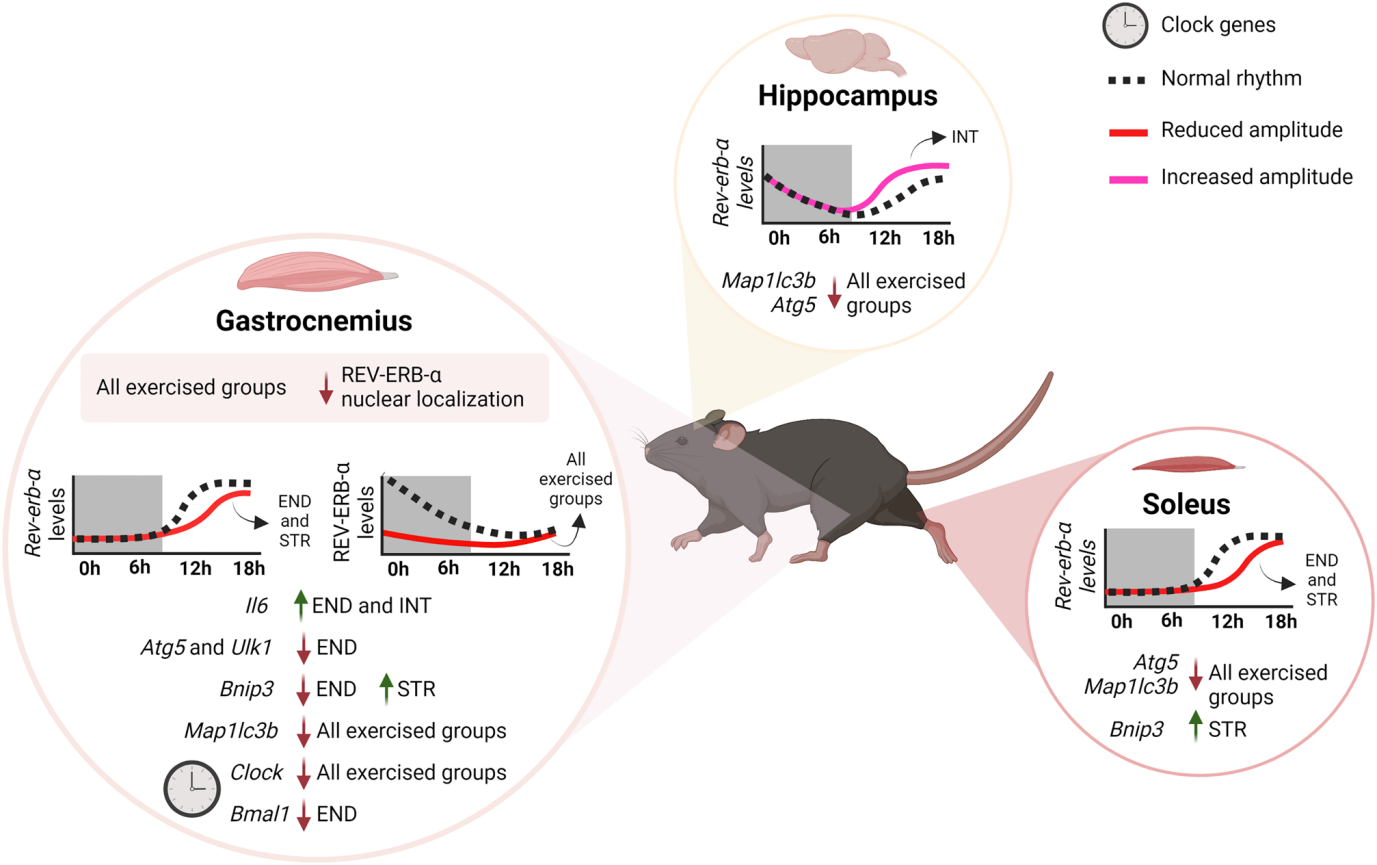
Schematic representation summarizing the main findings of the present study. *END* endurance, *INT* interval, *STR* strength. Created with BioRender.com.

In conclusion, the present investigation showed REV-ERB-α as an extremely sensitive molecule to physical exercise stimulus, including different models and intensities, both in skeletal muscle and hippocampus. REV-ERB-α transcriptional responses seem to be more influenced by the exercise intensity than the model (aerobic vs. strength). Also, it was shown that an acute exercise session excluded the REV-ERB-α from the nuclear compartment in gastrocnemius. This response did not culminate in the upregulation of the autophagic genes. Some of these genes were downregulated in peripheral tissue and the central nervous system. Specifically, concerning *Map1lc3b* in skeletal muscle, it seems that REV-ERB-α participates in repressing its expression in the aerobic exercise context. Figure 7 summarizes the main findings of the present investigation.

## Methods

### Experimental animals

Eight-week-old male C57BL/6 mice from the Central Animal Facility of the Ribeirão Preto campus from the University of São Paulo (USP) were used. The animals were accommodated in sterile micro-insulators (three animals per cage) in a ventilated rack (INSIGHT^®^, Ribeirão Preto, SP, Brazil) with a controlled temperature (22 ± 2 °C) on a 12:12-h light–dark cycle (light: 6 a.m. to 6 p.m.; dark: 6 p.m. to 6 a.m.). Food (Purina chow) and water were provided ad libitum. All experimental procedures were performed according to the Brazilian College of Animal Experimentation (COBEA), and following ARRIVE guidelines. Also, the experimental procedures were approved by the Ethics Committee of the University of São Paulo (I.D. 2017.5.33.90.7).

Mice were distributed into four experimental groups: Control (CT; sedentary), Endurance (END; submitted to an acute endurance exercise protocol), Interval (INT; submitted to an acute aerobic interval exercise protocol), and Strength (STR; submitted to an acute resistance exercise protocol). The sample size for each group/experiment is described in the figure legends. Mice from END and INT groups were submitted to 5 days of adaptation on a treadmill (INSIGHT™, Ribeirão Preto, SP, Brazil), 10 min per day, at a speed of 6 m/min. Also, mice from the STR group were submitted to five days of adaptation on a ladder-climbing (INSIGHT^®^, Ribeirão Preto, SP, Brazil) with and without external load. The ladder had 1110 mm of height, 80° of inclination, and 85 steps with a distance of 6 mm between each.

### Incremental load test (ILT)

After 24 h of the treadmill adaptation, the incremental load test started at an initial velocity of 6 m/min, at 0% inclination for the END and INT group, with 3 m/min increments every 3 min until voluntary exhaustion. The maximum velocity reached was classified as Maximum Aerobic Power (MAP) intensity. This value was used to prescribe the exercise intensity for the END and INT groups^32^.

### Acute exercise protocols

After 48 h of the last adaptation session, mice were weighed and initiated the specific acute exercise protocol at 6 pm. For the END group, mice ran at 60% of the MAP velocity at 0% inclination for 60 min. For the INT group, mice ran alternating intensities every 5 min between 40 and 80% of the MAP velocity for 60 min. Mice from the STR group performed one climb without external load to warm up. After that, an external load corresponding to 75% of body weight was attached at the base of the tail of each animal, and the mice performed ten climbs with a 2-min recovery between each repetition^33^.

### Blood glucose measure

The blood from the tail tip was collected, and glucose levels were measured before and immediately after the acute physical exercise protocols using a glycemic monitoring system (Accu-ChekTM Active model, Roche, Santo André, SP, Brazil).

### Tissue extraction

Immediately at the end, 6, 12, and 18 h after the acute exercise protocols, the animals were anesthetized by an intraperitoneal administration of xylazine (10 mg/kg of body weight) and ketamine (100 mg/kg of body weight). As soon as the loss of pedal reflexes confirmed the effect of anesthesia, mice were decapitated. Subsequently, the gastrocnemius, soleus, and hippocampus samples were removed, washed with sterile saline, and quickly frozen in liquid nitrogen. Afterward, the samples were stored at − 80 °C for further analysis utilizing Reverse Transcription-quantitative Polymerase Chain Reaction (RT-qPCR) and immunoblotting techniques.

### Reverse transcription-quantitative polymerase chain reaction (RT-qPCR)

Total RNA from skeletal muscles and hippocampus was extracted with TRIZOL (Invitrogen, Carlsbad, CA). All procedures were performed under standard RNase-free conditions to avoid exogenous RNase contamination. The Real time-quantitative PCR technique was performed by the StepOne™ Real-Time PCR System (Life Technologies Corporation, Carlsbad, CA, USA) for the analysis of mRNA expression for *Nr1d1* (Nuclear receptor subfamily 1, group D, member 1), *Atg5* (Autophagy protein 5), *Bnip3* (BCL2/adenovirus E1B 19 kDa protein-interacting protein 3), *Map1lc3b* (Microtubule-associated proteins 1A/1B light chain 3B), *Ulk1* (Serine/threonine-protein kinase ULK1), *Sqstm1* (Sequestosome-1), *Il6* (Interleukin-6), *Il1b* (Interleukin-1 β), *Tnfa* (Tumor Necrosis factor α), *Bmal1* (Brain and muscle ARNT-Like 1) and *Clock* (Circadian Locomotor Output Cycles Kaput).

RT-qPCR was performed using the following reagents: 5 µL of SYBR^®^ Green Master Mix (Bio-Rad, California, USA), 1 µL of forward primer, 1 µL of reverse primer (both at a final concentration of 100–200 nM), 1 µL cDNA (10 ng), and 1 µL of DEPC treated H_2_O. Each amplification reaction occurred with standard cycling with the following cycles: one cycle at 95 °C for 30 s, 40 cycles of 15 s at 95 °C, and 1 min at 60 °C. Relative quantitation was calculated by the 2^−ΔΔCT^ method using Thermo Fisher Cloud Software software, RQ version 3.7 (Life Technologies Corporation, Carlsbad, CA, USA). All values were corrected by the value obtained in *Gapdh* (Glyceraldehyde-3-phosphate dehydrogenase) amplification. Primer sequences are described in Table 1.

**Table 1:** Primer sequences.

Gene	Forward (5′–3′)	Reverse (5′–3′)
*Nr1d1*	AGAGAGGCCATCACAACCTC	TGTAGGTGATAACACCACCTGT
*Atg5*	GCTTTTGCCAAGAGTCAGCTAT	AACCAATTGGATAATGCCATTTCAG
*Bnip3*	CAGCATGAGAAACACAAGCG	TCCAATGTACCCCAAGCC
*Map1lc3b*	AGATAATCAGACGGCGCTTG	TCGTACACTTCGGAGATGGG
*Ulk1*	AACATCCGAGTCAAGATTGCTG	ATAATGACCTCAGGAGCCATGT
*Sqstm1*	ACAGCCAGAGGAACAGATGG	GTAGAGACTGGAGTTCACCTGTA
*Il6*	CTGCAAGAGACTTCCATCCAG	AGTGGTATAGACAGGTCTGTTGG
*Il1b*	TGCCACCTTTTGACAGTGATG	GCTCTTGTTGATGTGCTGCT
*Tnfa*	CAGGCGGTGCCTATGTCTC	CGATCACCCCGAAGTTCAGTAG
*Bmal1*	GGACTTCGCCTCTACCTGTTC	ACCCGTATTTCCCCGTTC
*Clock*	ATGGTGTTTACCGTAAGCTGTAG	CTCGCGTTACCAGGAAGCA
*Gapdh*	AAGAGGGATGCTGCCCTTAC	CGGGACGAGGAAACACTCTC

### Immunoblotting

The immunoblotting technique was performed as previously described by our research group^34^. The antibodies used were: REV-ERB-α (sc-393215) and Histone H3 (sc-517576) from Santa Cruz Biotechnology (Santa Cruz, CA, USA); GAPDH (#2118) from Cell Signaling Technology (Cell Signaling Technology, MA, USA). All the primary antibodies were utilized at a dilution of 1:1,000, and the secondary antibodies at a dilution between 1:5,000 and 1:10,000. Images were acquired by the ChemiDoc Imaging System (Bio-Rad, California, USA) and quantified using the software Image Studio for C-DiGit™ Blot Scanner. The subcellular fractionation was performed as previously described by Dimauro et al.^35^. The REV-ERB-α blots were normalized by the total protein normalization method by Ponceau S staining^36^. All immunoblotting images were checked for data integrity using the Proofig pipeline (https://www.proofig.com).

### Statistical analysis

Results are expressed as mean ± standard error of the mean (SEM). Levene’s test was used to verify the homogeneity of variances, and the Shapiro–Wilk W-test to check data normality. When normality was confirmed, a two-way analysis of variance (ANOVA) was used to compare the response of a specific protein/gene expression between exercise time and groups. Tukey's post hoc test was performed when the two-way ANOVA indicated significance. An unpaired Student's t-test was applied to investigate the possible differences between the two experimental groups. The paired Student’s t-test was used to compare the blood glucose before and after the exercise session. The one-way ANOVA compared three or four different experimental groups in a specific situation. Pearson’s or Spearman’s correlations were utilized to investigate possible correlations between REV-ERB-α protein levels and autophagy-related gene expressions. The *Nr1d1* RTqPCR values were used in the R statistical package^37^ v.4.2.1 utilizing the JTK_cycle algorithm to characterize the amplitude of circadian rhythm^38^. All statistical analyses were set at p < 0.05 and two-sided. Statistical analyses were performed using GraphPad Prism v.8.0.1 for Windows (GraphPad Software, CA, USA).

## Supplementary Information

Supplementary Information.

## Data Availability

All the experiment data and analyses are presented in the Supplementary Information File.
