# Supplementary material for: Exercise alters the circadian rhythm of REV-ERB-α and downregulates autophagy-related genes in peripheral and central tissues
Source: Sci Rep. 2022 Nov 21;12:20006. doi: 10.1038/s41598-022-24277-4 (PMC9678899; doi:10.1038/s41598-022-24277-4)
Supplement: Supplementary file 1 — Supplementary Information. [file 41598_2022_24277_MOESM1_ESM.pdf]

Full data of the article

Title: *Exercise alters the circadian rhythm of Rev-erb-α and downregulate autophagy-related genes in peripheral and central tissues*

Authors: *Alisson L. da Rocha, Ana P. Pinto, Bruno L. S. Bedo, Gustavo P. Morais, Luciana C. Oliveira, Ruither O. G. Carolino, Jose R. Pauli, Fernando M. Simabuco, Leandro P. de Moura, Eduardo R. Ropelle, Dennys E. Cintra, Donato A. Rivas, Adelino S. R. da Silva*

Scholarship or grant: *FAPESP 2017/12765-2*

Additional info: -

Date: OCT/20/2022

Figure 1

A

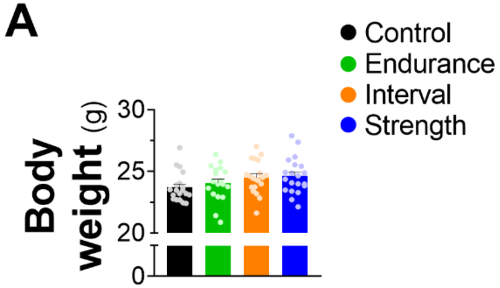

Control	Endurance	Interval	Strength
24.5	23.18	23.2	23.85
23.09	21.42	21.63	23.29
22.42	22.91	24.69	24.87
25.53	24.19	22.84	24.08
23.53	22.96	23.4	22.13
23.7	25.12	24	24.66
23.48	20.88	24.44	24.88
23.49	24.26	24.86	23.41
23.11	24.96	26.1	24.37
23.91	24.84	23.73	23.98
21.47	25.47	25.94	24.93
23.21	23.77	26.05	25.94
26.93	25.79	24.75	25.55
23.21	26.39	24.62	27.38
22.77	23.97	23.74	27.9
24.96	25.3	27.03	22.72
25.4	23.98	24.35	24
23.73	23.65	24.52	23.5
24.04		26.39	25.43
		24.26	26.19

	Control	Endurance	Interval	Strength
Mean	23.81	24.06	24.53	24.65
Std. Deviation	1.238	1.439	1.311	1.457
Std. Error of Mean	0.2841	0.3392	0.2931	0.3259
**Shapiro-Wilk test**				
W	0.9454	0.9603	0.9725	0.9689
P value	0.3289	0.6065	0.8057	0.7313
Passed normality test (alpha=0.05)?	Yes	Yes	Yes	Yes
**ANOVA summary**				
F	1.613			
P value	0.1937			
P value summary	ns			
Significant diff. among means (P < 0.05)?	No			
R square	0.06217			

C

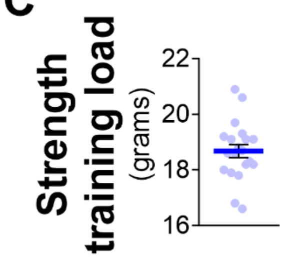

	Strength training load
	16.6
	18.6
	18.6
	18.7
	17.9
	19.2
	19.1
	18
	17.8
	19.1
	18.2
	19.3
	19.1
	20.6
	18.8
	18.3
	19.7
	20.9
	18.2
	16.8
Mean	18.68
Std. Deviation	1.056
Std. Error of Mean	0.2361

Figure 1

D

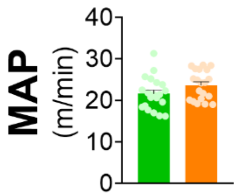

	Endurance	Interval
	25.7	22.3
	18.4	28.4
	17	24.3
	22.3	20.4
	20.6	27.6
	16.2	19.2
	18.7	27.5
	21.4	28.3
	23.8	24.2
	16.3	28.5
	24.3	25.1
	27.2	25.2
	20.7	20.1
	22.5	21.7
	25.2	19.7
	19.6	21
	21.8	27.4
	31.3	19
	17.7	19.1
	21.4	24.9
Mean	21.61	23.7
Std. Deviation	3.886	3.498
Std. Error of Mean	0.8688	0.7822
**Shapiro-Wilk test**		
W	0.9583	0.8949
P value	0.5103	0.0332
Passed normality test (alpha=0.05)?	Yes	No
P value summary	ns	*
**Mann Whitney test**		
P value	0.0821	
Exact or approximate P value?	Exact	
P value summary	ns	
Significantly different (P < 0.05)?	No	
One- or two-tailed P value?	Two-tailed	
Sum of ranks in column A,B	345.5 , 474.5	
Mann-Whitney U	135.5	

E

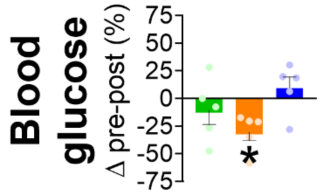

	Endurance		Interval	
	pre	post	pre	post
	177	227	169	110
	173	173	176	140
	168	88	183	76
	146	135	185	153
	158	116	171	135
Mean	164.4	147.8	176.8	122.8
Std. Deviation	12.5	53.99	7.085	30.46
Std. Error of Mean	5.591	24.14	3.169	13.62
**Shapiro-Wilk test**				
W	0.9386	0.9663	0.9117	0.9197
P value	0.6561	0.8512	0.4781	0.5277
Passed normality test (alpha=0.05)?	Yes	Yes	Yes	Yes
P value summary	ns	ns	ns	ns
**Paired t test**				
P value	0.4867		0.0185	
P value summary	ns		*	
Significantly different (P < 0.05)?	No		Yes	
One- or two-tailed P value?	Two-tailed		Two-tailed	
t, df	t=0.7654, df=4		t=3.835, df=4	
Number of pairs	5		5	

	Strength	
	pre	post
	193	139
	192	204
	167	200
	161	191
	155	202
Mean	173.6	187.2
Std. Deviation	17.77	27.4
Std. Error of Mean	7.947	12.25
**Shapiro-Wilk test**		
W	0.8485	0.7003
P value	0.1898	0.0096
Passed normality test (alpha=0.05)?	Yes	No
P value summary	ns	**
**Wilcoxon matched-pairs signed rank test**		
P value	0.625	
Exact or approximate P value?	Exact	
P value summary	ns	
Significantly different (P < 0.05)?	No	
One- or two-tailed P value?	Two-tailed	
Sum of positive, negative ranks	10.00 , -5.000	
Sum of signed ranks (W)	5	
Number of pairs	5	
Number of ties (ignored)	0	

Figure 2 - Gastrocnemius

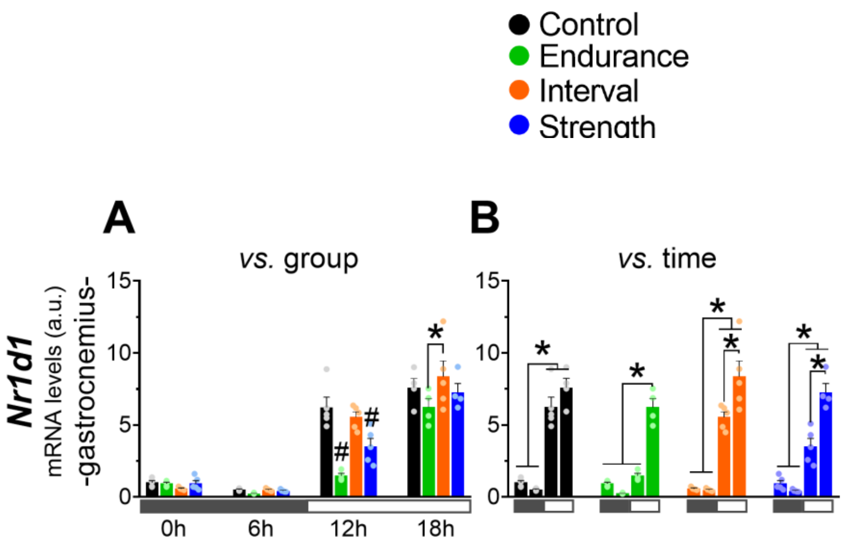

Gastrocnemius

Time course - 0h	Control	Endurance	Interval	Strength
	1.052853	1.124447	-	1.602443
	0.691725	1.070752	0.567488	1.192883
	1.367656	0.699095	0.725416	0.700147
	1.156033	0.750684	0.533797	0.663298
	0.731733	0.979154	0.477995	0.495894
Mean	1	0.9248	0.5762	0.9309
Std. Deviation	0.2869	0.1906	0.1061	0.4565
Std. Error of Mean	0.1283	0.08526	0.05306	0.2042
Shapiro-Wilk test				
W	0.9241	0.8913	0.9132	0.8939
P value	0.557	0.3636	0.4997	0.3771
Passed normality test (alpha=0.05)?	Yes	Yes	Yes	Yes
P value summary	ns	ns	ns	ns

Time course - 6h	Control	Endurance	Interval	Strength
	0.604338	0.214782	0.43904	0.349547
	0.45378	0.225311	0.489577	0.552748
	0.565382	0.292693	0.618025	0.41693
	0.41693	0.234786	0.393767	0.364287
	-	0.214782	0.510634	0.415877
Mean	0.5101	0.2365	0.4902	0.4199
Std. Deviation	0.08905	0.03252	0.08467	0.08018
Std. Error of Mean	0.04452	0.01454	0.03786	0.03586
Shapiro-Wilk test				
W	0.9128	0.7578	0.9648	0.849
P value	0.4975	0.0351	0.8413	0.1914
Passed normality test (alpha=0.05)?	Yes	No	Yes	Yes
P value summary	ns	*	ns	ns

Time course - 12h	Control	Endurance	Interval	Strength
	5.488524	1.179196	4.485155	2.575279
	6.161297	1.268688	6.350811	2.170983
	4.906296	1.925669	4.872605	3.460729
	5.739103	1.510844	6.178143	5.089493
	8.876606	-	5.851758	4.303011
Mean	6.234	1.471	5.548	3.52
Std. Deviation	1.545	0.3339	0.8246	1.203
Std. Error of Mean	0.6911	0.1669	0.3688	0.5382
Shapiro-Wilk test				
W	0.8215	0.9155	0.8892	0.9589
P value	0.1198	0.5122	0.3531	0.8004
Passed normality test (alpha=0.05)?	Yes	Yes	Yes	Yes
P value summary	ns	ns	ns	ns

Time course - 18h	Control	Endurance	Interval	Strength
	9.013477	5.627501	8.892398	6.991998
	7.900611	6.868814	6.833017	9.038745
	7.518425	4.942093	12.1973	6.204464
	5.930722	7.526848	7.919562	6.824595
	-	-	6.065487	-
Mean	7.591	6.241	8.382	7.265
Std. Deviation	1.276	1.171	2.387	1.23
Std. Error of Mean	0.6378	0.5853	1.068	0.615
Shapiro-Wilk test				
W	0.9756	0.9564	0.9171	0.8599
P value	0.876	0.7562	0.5117	0.26
Passed normality test (alpha=0.05)?	Yes	Yes	Yes	Yes
P value summary	ns	ns	ns	ns

Figure 2 - Gastrocnemius

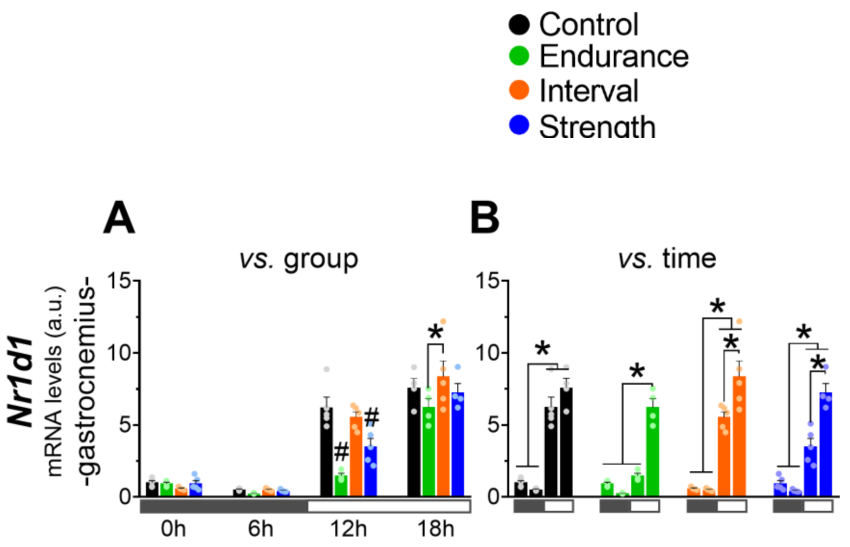

Two-way ANOVA	Ordinary
Alpha	0.05

Source of Variation	% of total variation	P value	P value summary	Significant?
Interaction	6.148	<0.0001	****	Yes
Row Factor	79.08	<0.0001	****	Yes
Column Factor	4.3	<0.0001	****	Yes

ANOVA table	SS (Type III)	DF	MS	F (DFn, DFd)	P value
Interaction	43.53	9	4.837	F (9, 58) = 4.996	P<0.0001
Row Factor	559.9	3	186.6	F (3, 58) = 192.8	P<0.0001
Column Factor	30.45	3	10.15	F (3, 58) = 10.48	P<0.0001
Residual	56.15	58	0.9681		

Tukey's multiple comparisons test	Mean Diff.	95.00% CI of diff.	Significant?	Summary	Adjusted P Value
0h					
Control vs. Endurance	0.07517	-1.571 to 1.721	No	ns	0.9994
Control vs. Interval	0.4238	-1.322 to 2.170	No	ns	0.9179
Control vs. Strength	0.06907	-1.577 to 1.715	No	ns	0.9995
Endurance vs. Interval	0.3487	-1.397 to 2.095	No	ns	0.9519
Endurance vs. Strength	-0.006107	-1.652 to 1.640	No	ns	>0.9999
Interval vs. Strength	-0.3548	-2.101 to 1.391	No	ns	0.9495
6h					
Control vs. Endurance	0.2736	-1.472 to 2.020	No	ns	0.9758
Control vs. Interval	0.0199	-1.726 to 1.766	No	ns	>0.9999
Control vs. Strength	0.09023	-1.656 to 1.836	No	ns	0.9991
Endurance vs. Interval	-0.2537	-1.900 to 1.392	No	ns	0.9769
Endurance vs. Strength	-0.1834	-1.829 to 1.463	No	ns	0.991
Interval vs. Strength	0.07033	-1.576 to 1.716	No	ns	0.9995
12h					
Control vs. Endurance	4.763	3.017 to 6.509	Yes	****	<0.0001
Control vs. Interval	0.6867	-0.9594 to 2.333	No	ns	0.6889
Control vs. Strength	2.714	1.068 to 4.360	Yes	***	0.0003
Endurance vs. Interval	-4.077	-5.822 to -2.331	Yes	****	<0.0001
Endurance vs. Strength	-2.049	-3.795 to -0.3029	Yes	*	0.0152
Interval vs. Strength	2.028	0.3818 to 3.674	Yes	**	0.0099
18h					
Control vs. Endurance	1.349	-0.4908 to 3.190	No	ns	0.2231
Control vs. Interval	-0.7907	-2.537 to 0.9551	No	ns	0.6306
Control vs. Strength	0.3259	-1.514 to 2.166	No	ns	0.9657
Endurance vs. Interval	-2.14	-3.886 to -0.3944	Yes	*	0.0103
Endurance vs. Strength	-1.024	-2.864 to 0.8167	No	ns	0.4612
Interval vs. Strength	1.117	-0.6293 to 2.862	No	ns	0.3373

Figure 2 - Gastrocnemius

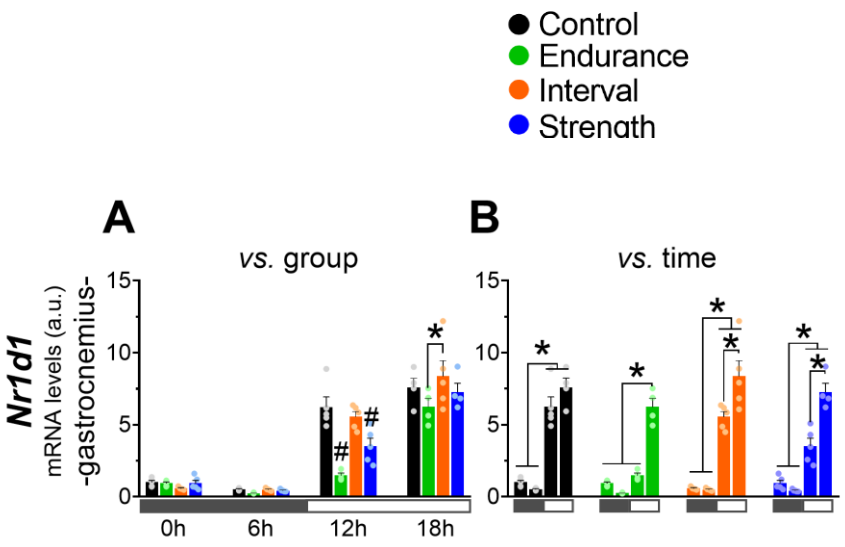

Two-way ANOVA	Ordinary
Alpha	0.05

Source of Variation	% of total variation	P value	P value summary	Significant?
Interaction	6.148	<0.0001	****	Yes
Row Factor	79.08	<0.0001	****	Yes
Column Factor	4.3	<0.0001	****	Yes

ANOVA table	SS (Type III)	DF	MS	F (DFn, DFd)	P value
Interaction	43.53	9	4.837	F (9, 58) = 4.996	P<0.0001
Row Factor	559.9	3	186.6	F (3, 58) = 192.8	P<0.0001
Column Factor	30.45	3	10.15	F (3, 58) = 10.48	P<0.0001
Residual	56.15	58	0.9681		

Tukey's multiple comparisons test	Mean Diff.	95.00% CI of diff.	Significant?	Summary	Adjusted P Value
Control					
0h vs. 6h	0.4899	-1.256 to 2.236	No	ns	0.8796
0h vs. 12h	-5.234	-6.880 to -3.588	Yes	****	<0.0001
0h vs. 18h	-6.591	-8.337 to -4.845	Yes	****	<0.0001
6h vs. 12h	-5.724	-7.470 to -3.978	Yes	****	<0.0001
6h vs. 18h	-7.081	-8.921 to -5.240	Yes	****	<0.0001
12h vs. 18h	-1.356	-3.102 to 0.3894	No	ns	0.1802
Endurance					
0h vs. 6h	0.6884	-0.9577 to 2.334	No	ns	0.6872
0h vs. 12h	-0.5463	-2.292 to 1.200	No	ns	0.8411
0h vs. 18h	-5.316	-7.062 to -3.571	Yes	****	<0.0001
6h vs. 12h	-1.235	-2.981 to 0.5113	No	ns	0.2519
6h vs. 18h	-6.005	-7.751 to -4.259	Yes	****	<0.0001
12h vs. 18h	-4.77	-6.611 to -2.930	Yes	****	<0.0001
Interval					
0h vs. 6h	0.08597	-1.660 to 1.832	No	ns	0.9992
0h vs. 12h	-4.972	-6.717 to -3.226	Yes	****	<0.0001
0h vs. 18h	-7.805	-9.551 to -6.059	Yes	****	<0.0001
6h vs. 12h	-5.057	-6.704 to -3.411	Yes	****	<0.0001
6h vs. 18h	-7.891	-9.537 to -6.245	Yes	****	<0.0001
12h vs. 18h	-2.834	-4.480 to -1.188	Yes	***	0.0002
Strength					
0h vs. 6h	0.5111	-1.135 to 2.157	No	ns	0.8442
0h vs. 12h	-2.589	-4.235 to -0.9429	Yes	***	0.0006
0h vs. 18h	-6.334	-8.080 to -4.588	Yes	****	<0.0001
6h vs. 12h	-3.1	-4.746 to -1.454	Yes	****	<0.0001
6h vs. 18h	-6.845	-8.591 to -5.099	Yes	****	<0.0001
12h vs. 18h	-3.745	-5.491 to -1.999	Yes	****	<0.0001

Figure 2 - Soleus

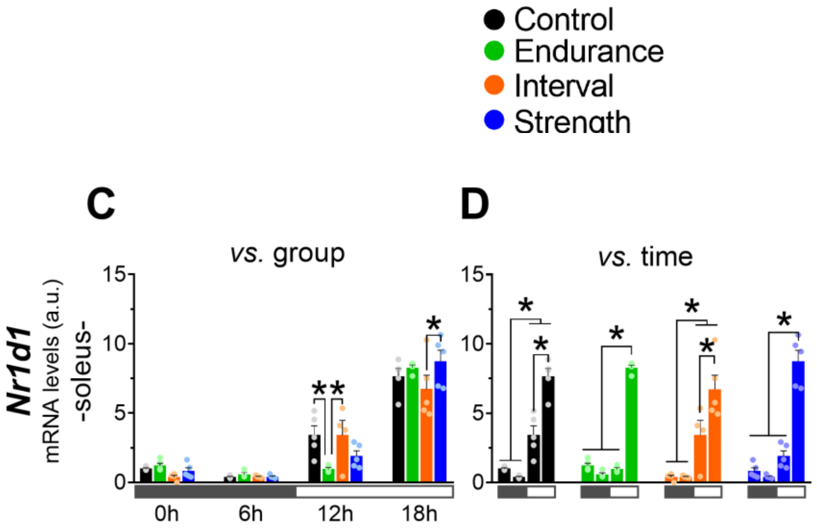

Soleus

Time course - 0h	Control	Endurance	Interval	Strength
	0.887784	1.789773	0.478516	1.633523
	0.976563	1.419567	0.611683	1.058239
	1.18608	1.020064	0.450107	0.482955
	0.995206	0.927734	0	0.587713
	0.954368	0.924183		0.391513
Mean	1	1.216	0.5134	0.8308
Std. Deviation	0.1117	0.3799	0.08626	0.517
Std. Error of Mean	0.04994	0.1699	0.0498	0.2312
Shapiro-Wilk test				
W	0.8685	0.8343	0.8771	0.8698
P value	0.2603	0.1498	0.316	0.2656
Passed normality test (alpha=0.05)?	Yes	Yes	Yes	Yes
P value summary	ns	ns	ns	ns

Time course - 6h	Control	Endurance	Interval	Strength
	0.348011	0.440341	0.403054	0.29652
	0.369318	0.414595	0.370206	0.390625
	0.29652	0.954368	0.41282	0.608132
	0.528232	0.470526	0.521129	0.366655
			0.332031	0.350675
Mean	0.3855	0.57	0.4078	0.4025
Std. Deviation	0.09993	0.2573	0.07079	0.12
Std. Error of Mean	0.04996	0.1286	0.03166	0.05368
Shapiro-Wilk test				
W	0.8846	0.7118	0.9223	0.8153
P value	0.3588	0.0158	0.5446	0.1073
Passed normality test (alpha=0.05)?	Yes	No	Yes	Yes
P value summary	ns	*	ns	ns

Time course - 12h	Control	Endurance	Interval	Strength
	1.528764	0.941051	0.430575	1.057351
	4.533026	0.879794	3.78196	1.201172
	2.685547	1.271307	5.355114	1.658381
	3.25728	0.753729	4.108665	2.668679
	5.167791			2.917259
Mean	3.434	0.9615	3.419	1.901
Std. Deviation	1.452	0.2208	2.104	0.8489
Std. Error of Mean	0.6494	0.1104	1.052	0.3796
Shapiro-Wilk test				
W	0.9728	0.9155	0.89	0.8775
P value	0.8928	0.512	0.3832	0.298
Passed normality test (alpha=0.05)?	Yes	Yes	Yes	Yes
P value summary	ns	ns	ns	ns

Time course - 18h	Control	Endurance	Interval	Strength
	8.18892	8.592862	5.209517	6.954013
	7.519531	8.362926	4.934304	9.443359
	8.108132	8.46946	7.498224	10.64187
	8.876953	7.674893	5.761719	9.892578
	5.613459		10.28498	6.792436
Mean	7.661	8.275	6.738	8.745
Std. Deviation	1.242	0.411	2.219	1.762
Std. Error of Mean	0.5554	0.2055	0.9925	0.7881
Shapiro-Wilk test				
W	0.8804	0.8302	0.8568	0.8596
P value	0.3111	0.1684	0.2171	0.2266
Passed normality test (alpha=0.05)?	Yes	Yes	Yes	Yes
P value summary	ns	ns	ns	ns

Figure 2 - Soleus

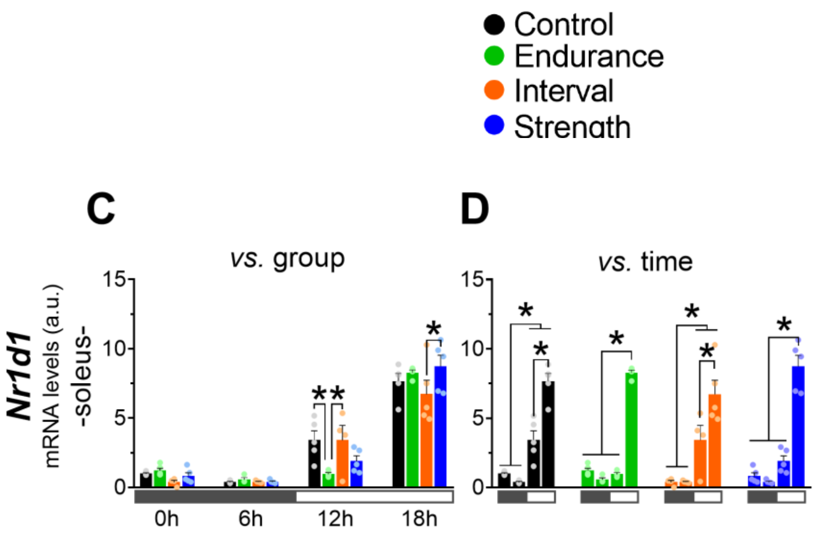

Two-way ANOVA	Ordinary
Alpha	0.05

Source of Variation	% of total variation	P value	P value summary	Significant?
Interaction	3.952	0.0066	**	Yes
Row Factor	86.95	<0.0001	****	Yes
Column Factor	0.2442	0.658	ns	No

ANOVA table	SS (Type III)	DF	MS	F (DFn, DFd)	P value
Interaction	29.63	9	3.292	F (9, 58) = 2.904	P=0.0066
Row Factor	651.9	3	217.3	F (3, 58) = 191.6	P<0.0001
Column Factor	1.831	3	0.6103	F (3, 58) = 0.5383	P=0.6580
Residual	65.77	58	1.134		

Tukey's multiple comparisons test	Mean Diff.	95.00% CI of diff.	Significant?	Summary	Adjusted P Value
0h					
Control vs. Endurance	-0.2163	-1.998 to 1.565	No	ns	0.9884
Control vs. Interval	0.6149	-1.275 to 2.504	No	ns	0.8249
Control vs. Strength	0.1692	-1.612 to 1.951	No	ns	0.9944
Endurance vs. Interval	0.8312	-1.058 to 2.721	No	ns	0.652
Endurance vs. Strength	0.3855	-1.396 to 2.167	No	ns	0.9399
Interval vs. Strength	-0.4457	-2.335 to 1.444	No	ns	0.924
6h					
Control vs. Endurance	-0.1844	-2.176 to 1.807	No	ns	0.9948
Control vs. Interval	-0.02233	-1.912 to 1.867	No	ns	>0.9999
Control vs. Strength	-0.017	-1.906 to 1.872	No	ns	>0.9999
Endurance vs. Interval	0.1621	-1.727 to 2.052	No	ns	0.9958
Endurance vs. Strength	0.1674	-1.722 to 2.057	No	ns	0.9954
Interval vs. Strength	0.005327	-1.776 to 1.787	No	ns	>0.9999
12h					
Control vs. Endurance	2.473	0.5835 to 4.362	Yes	**	0.0054
Control vs. Interval	0.0154	-1.874 to 1.905	No	ns	>0.9999
Control vs. Strength	1.534	-0.2475 to 3.315	No	ns	0.1152
Endurance vs. Interval	-2.458	-4.449 to -0.4659	Yes	**	0.0097
Endurance vs. Strength	-0.9391	-2.829 to 0.9504	No	ns	0.5575
Interval vs. Strength	1.519	-0.3710 to 3.408	No	ns	0.1571
18h					
Control vs. Endurance	-0.6136	-2.503 to 1.276	No	ns	0.8258
Control vs. Interval	0.9237	-0.8578 to 2.705	No	ns	0.5221
Control vs. Strength	-1.083	-2.865 to 0.6980	No	ns	0.3818
Endurance vs. Interval	1.537	-0.3522 to 3.427	No	ns	0.1491
Endurance vs. Strength	-0.4698	-2.359 to 1.420	No	ns	0.9124
Interval vs. Strength	-2.007	-3.789 to -0.2257	Yes	*	0.0213

Figure 2 - Soleus

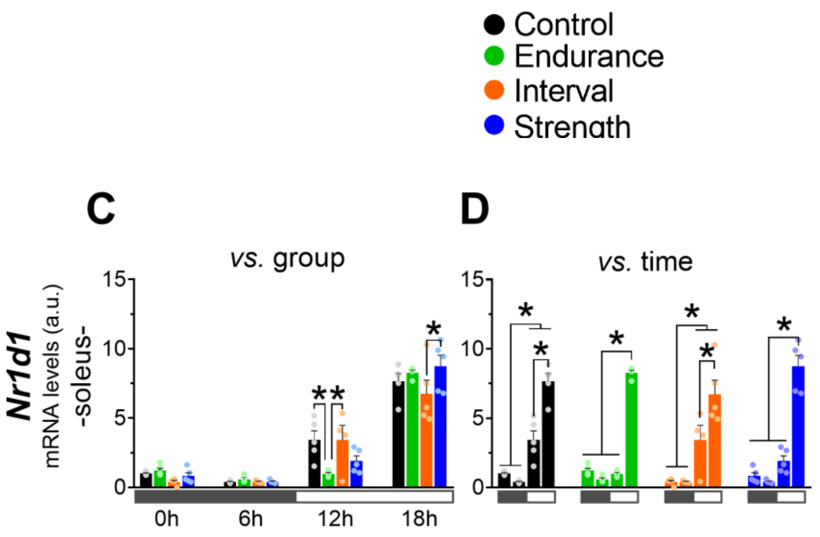

Two-way ANOVA	Ordinary
Alpha	0.05

Source of Variation	% of total variation	P value	P value summary	Significant?
Interaction	3.952	0.0066	**	Yes
Row Factor	86.95	<0.0001	****	Yes
Column Factor	0.2442	0.658	ns	No

ANOVA table	SS (Type III)	DF	MS	F (DFn, DFd)	P value
Interaction	29.63	9	3.292	F (9, 58) = 2.904	P=0.0066
Row Factor	651.9	3	217.3	F (3, 58) = 191.6	P<0.0001
Column Factor	1.831	3	0.6103	F (3, 58) = 0.5383	P=0.6580
Residual	65.77	58	1.134		

Tukey's multiple comparisons test	Mean Diff.	95.00% CI of diff.	Significant?	Summary	Adjusted P Value
Control					
0h vs. 6h	0.6145	-1.275 to 2.504	No	ns	0.8252
0h vs. 12h	-2.434	-4.216 to -0.6531	Yes	**	0.0034
0h vs. 18h	-6.661	-8.443 to -4.880	Yes	****	<0.0001
6h vs. 12h	-3.049	-4.938 to -1.159	Yes	***	0.0004
6h vs. 18h	-7.276	-9.165 to -5.386	Yes	****	<0.0001
12h vs. 18h	-4.227	-6.008 to -2.446	Yes	****	<0.0001
Endurance					
0h vs. 6h	0.6463	-1.243 to 2.536	No	ns	0.8023
0h vs. 12h	0.2548	-1.635 to 2.144	No	ns	0.9843
0h vs. 18h	-7.059	-8.948 to -5.169	Yes	****	<0.0001
6h vs. 12h	-0.3915	-2.383 to 1.600	No	ns	0.954
6h vs. 18h	-7.705	-9.697 to -5.713	Yes	****	<0.0001
12h vs. 18h	-7.314	-9.305 to -5.322	Yes	****	<0.0001
Interval					
0h vs. 6h	-0.02277	-1.912 to 1.867	No	ns	>0.9999
0h vs. 12h	-3.034	-5.026 to -1.042	Yes	***	0.0009
0h vs. 18h	-6.353	-8.242 to -4.463	Yes	****	<0.0001
6h vs. 12h	-3.011	-4.901 to -1.122	Yes	***	0.0005
6h vs. 18h	-6.33	-8.111 to -4.548	Yes	****	<0.0001
12h vs. 18h	-3.319	-5.208 to -1.429	Yes	***	0.0001
Strength					
0h vs. 6h	0.4283	-1.353 to 2.210	No	ns	0.92
0h vs. 12h	-1.07	-2.851 to 0.7116	No	ns	0.3931
0h vs. 18h	-7.914	-9.695 to -6.133	Yes	****	<0.0001
6h vs. 12h	-1.498	-3.279 to 0.2834	No	ns	0.1287
6h vs. 18h	-8.342	-10.12 to -6.561	Yes	****	<0.0001
12h vs. 18h	-6.844	-8.626 to -5.063	Yes	****	<0.0001

Figure 2 - Hippocampus

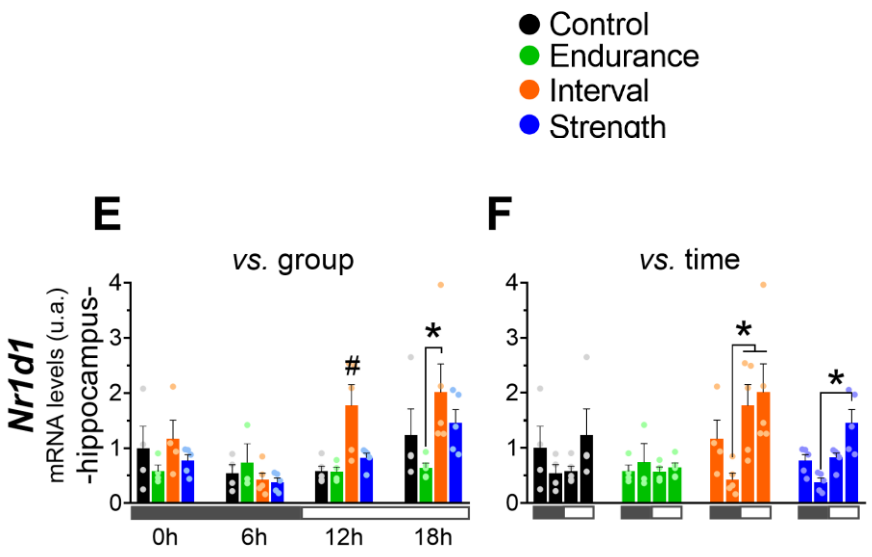

Hippocampus

Time course - 0h	Control	Endurance	Interval	Strength
	2.077922	0.552727	2.115325	0.442597
	1.065974	0.903896	0.527792	0.592208
	0.251429	0.463377	1.090909	0.976623
	0.604675	0.396883	0.937143	0.881039
				0.966234
Mean	1	0.5792	1.168	0.7717
Std. Deviation	0.7922	0.2257	0.6749	0.241
Std. Error of Mean	0.3961	0.1128	0.3375	0.1078
**Shapiro-Wilk test**				
W	0.9423	0.864	0.9124	0.8532
P value	0.6684	0.2746	0.4954	0.2048
Passed normality test (alpha=0.05)?	Yes	Yes	Yes	Yes
P value summary	ns	ns	ns	ns

Time course - 6h	Control	Endurance	Interval	Strength
	0.702338	0.361558	0.521558	0.182857
	0.893506	1.419221	0.322078	0.409351
	0.367792	0.442597	0.274286	0.527792
	0.22026		0.847792	0.554805
			0.164156	0.232727
Mean	0.546	0.7411	0.426	0.3815
Std. Deviation	0.3072	0.5886	0.269	0.1687
Std. Error of Mean	0.1536	0.3399	0.1203	0.07543
**Shapiro-Wilk test**				
W	0.9492	0.8071	0.913	0.891
P value	0.711	0.1316	0.4861	0.362
Passed normality test (alpha=0.05)?	Yes	Yes	Yes	Yes
P value summary	ns	ns	ns	ns

Time course - 12h	Control	Endurance	Interval	Strength
	0.428052	0.637922	2.491429	0.511169
	0.401039	0.407273	0.770909	0.935065
	0.494545	0.783377	2.539221	0.901818
	0.905974	0.450909	2.09039	0.96
	0.658701		0.968312	0.831169
Mean	0.5777	0.5699	1.772	0.8278
Std. Deviation	0.2091	0.174	0.8449	0.1835
Std. Error of Mean	0.0935	0.08699	0.3779	0.08208
**Shapiro-Wilk test**				
W	0.8756	0.9235	0.8336	0.7762
P value	0.2898	0.5569	0.1481	0.0511
Passed normality test (alpha=0.05)?	Yes	Yes	Yes	Yes
P value summary	ns	ns	ns	ns

Time course - 18h	Control	Endurance	Interval	Strength
	0.872727	0.691948	1.456623	0.885195
	0.851948	0.471688	3.962597	1.967792
	0.571429	0.930909	1.259221	0.980779
	2.649351	0.635844	1.265455	2.055065
		0.486234	2.123636	1.39013
Mean	1.236	0.6433	2.014	1.456
Std. Deviation	0.952	0.1866	1.146	0.5424
Std. Error of Mean	0.476	0.08345	0.5123	0.2426
**Shapiro-Wilk test**				
W	0.7538	0.9043	0.7649	0.8768
P value	0.0419	0.434	0.0407	0.2949
Passed normality test (alpha=0.05)?	No	Yes	No	Yes
P value summary	*	ns	*	ns

Figure 2 - Hippocampus

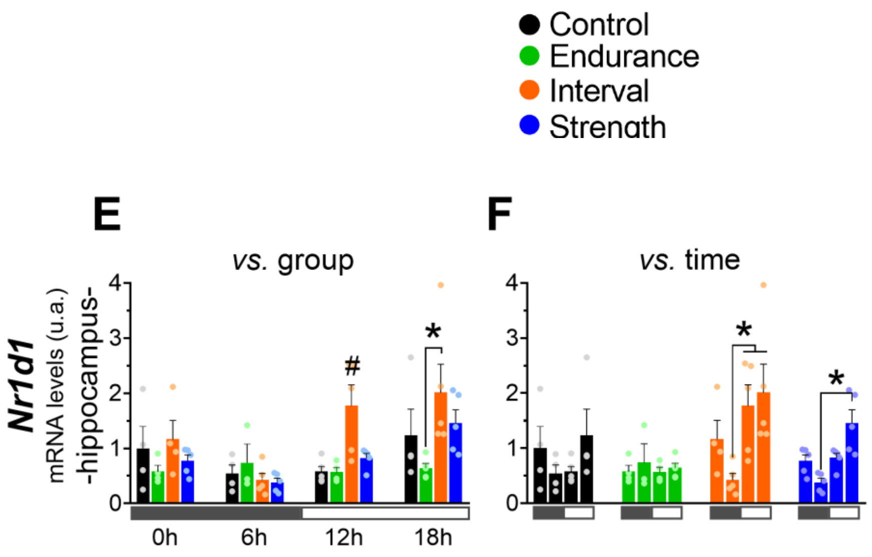

Two-way ANOVA	Ordinary
Alpha	0.05

Source of Variation	% of total variation	P value	P value summary	Significant?
Interaction	14.15	0.1015	ns	No
Row Factor	16.88	0.001	**	Yes
Column Factor	13.95	0.0033	**	Yes

ANOVA table	SS (Type III)	DF	MS	F (DFn, DFd)	P value
Interaction	4.907	9	0.5452	F (9, 56) = 1.739	P=0.1015
Row Factor	5.851	3	1.95	F (3, 56) = 6.221	P=0.0010
Column Factor	4.837	3	1.612	F (3, 56) = 5.143	P=0.0033
Residual	17.55	56	0.3135		

Tukey's multiple comparisons test	Mean Diff.	95.00% CI of diff.	Significant?	Summary	Adjusted P Value
0h					
Control vs. Endurance	0.4208	-0.6275 to 1.469	No	ns	0.7133
Control vs. Interval	-0.1678	-1.216 to 0.8805	No	ns	0.9742
Control vs. Strength	0.2283	-0.7662 to 1.223	No	ns	0.9292
Endurance vs. Interval	-0.5886	-1.637 to 0.4597	No	ns	0.4522
Endurance vs. Strength	-0.1925	-1.187 to 0.8020	No	ns	0.9558
Interval vs. Strength	0.3961	-0.5984 to 1.391	No	ns	0.7183
6h					
Control vs. Endurance	-0.1952	-1.327 to 0.9371	No	ns	0.9681
Control vs. Interval	0.12	-0.8745 to 1.114	No	ns	0.9886
Control vs. Strength	0.1645	-0.8300 to 1.159	No	ns	0.9716
Endurance vs. Interval	0.3152	-0.7675 to 1.398	No	ns	0.8673
Endurance vs. Strength	0.3596	-0.7231 to 1.442	No	ns	0.8154
Interval vs. Strength	0.04447	-0.8932 to 0.9821	No	ns	0.9993
12h					
Control vs. Endurance	0.007792	-0.9867 to 1.002	No	ns	>0.9999
Control vs. Interval	-1.194	-2.132 to -0.2568	Yes	**	0.0072
Control vs. Strength	-0.2502	-1.188 to 0.6874	No	ns	0.8941
Endurance vs. Interval	-1.202	-2.197 to -0.2077	Yes	*	0.0118
Endurance vs. Strength	-0.258	-1.252 to 0.7365	No	ns	0.9017
Interval vs. Strength	0.9442	0.006586 to 1.882	Yes	*	0.0478
18h					
Control vs. Endurance	0.593	-0.4015 to 1.588	No	ns	0.3987
Control vs. Interval	-0.7771	-1.772 to 0.2174	No	ns	0.1758
Control vs. Strength	-0.2194	-1.214 to 0.7751	No	ns	0.9364
Endurance vs. Interval	-1.37	-2.308 to -0.4326	Yes	**	0.0016
Endurance vs. Strength	-0.8125	-1.750 to 0.1252	No	ns	0.1116
Interval vs. Strength	0.5577	-0.3799 to 1.495	No	ns	0.401

Figure 2 - Hippocampus

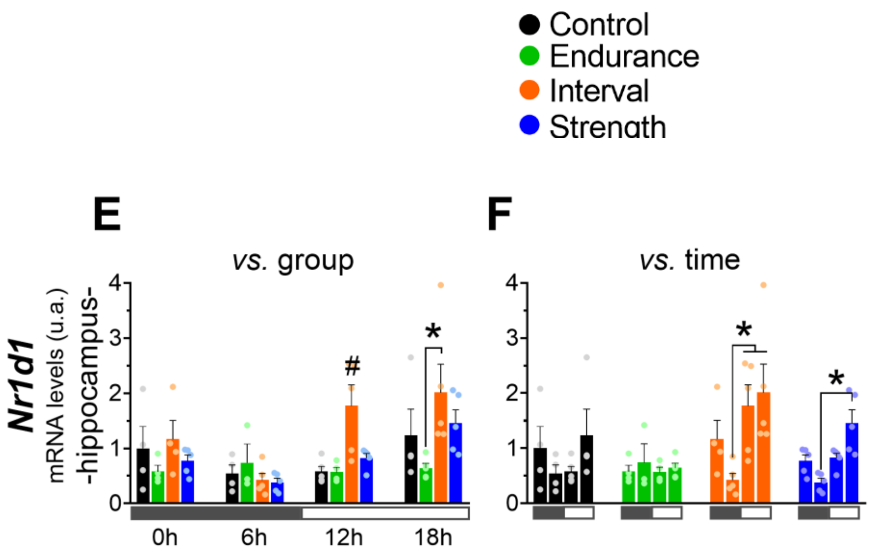

Two-way ANOVA	Ordinary
Alpha	0.05

Source of Variation	% of total variation	P value	P value summary	Significant?
Interaction	14.15	0.1015	ns	No
Row Factor	16.88	0.001	**	Yes
Column Factor	13.95	0.0033	**	Yes

ANOVA table	SS (Type III)	DF	MS	F (DFn, DFd)	P value
Interaction	4.907	9	0.5452	F (9, 56) = 1.739	P=0.1015
Row Factor	5.851	3	1.95	F (3, 56) = 6.221	P=0.0010
Column Factor	4.837	3	1.612	F (3, 56) = 5.143	P=0.0033
Residual	17.55	56	0.3135		

Tukey's multiple comparisons test	Mean Diff.	95.00% CI of diff.	Significant?	Summary	Adjusted P Value
Control					
0h vs. 6h	0.454	-0.5943 to 1.502	No	ns	0.6624
0h vs. 12h	0.4223	-0.5722 to 1.417	No	ns	0.6761
0h vs. 18h	-0.2364	-1.285 to 0.8119	No	ns	0.9326
6h vs. 12h	-0.03169	-1.026 to 0.9628	No	ns	0.9998
6h vs. 18h	-0.6904	-1.739 to 0.3579	No	ns	0.3112
12h vs. 18h	-0.6587	-1.653 to 0.3358	No	ns	0.3063
Endurance					
0h vs. 6h	-0.1619	-1.294 to 0.9704	No	ns	0.9813
0h vs. 12h	0.00935	-1.039 to 1.058	No	ns	>0.9999
0h vs. 18h	-0.0641	-1.059 to 0.9304	No	ns	0.9982
6h vs. 12h	0.1713	-0.9610 to 1.304	No	ns	0.978
6h vs. 18h	0.0978	-0.9849 to 1.180	No	ns	0.9951
12h vs. 18h	-0.07345	-1.068 to 0.9210	No	ns	0.9973
Interval					
0h vs. 6h	0.7418	-0.2527 to 1.736	No	ns	0.2096
0h vs. 12h	-0.6043	-1.599 to 0.3902	No	ns	0.382
0h vs. 18h	-0.8457	-1.840 to 0.1488	No	ns	0.1221
6h vs. 12h	-1.346	-2.284 to -0.4085	Yes	**	0.002
6h vs. 18h	-1.588	-2.525 to -0.6499	Yes	***	0.0002
12h vs. 18h	-0.2415	-1.179 to 0.6962	No	ns	0.9036
Strength					
0h vs. 6h	0.3902	-0.5474 to 1.328	No	ns	0.6898
0h vs. 12h	-0.0561	-0.9937 to 0.8815	No	ns	0.9986
0h vs. 18h	-0.6841	-1.622 to 0.2536	No	ns	0.2267
6h vs. 12h	-0.4463	-1.384 to 0.4913	No	ns	0.5916
6h vs. 18h	-1.074	-2.012 to -0.1367	Yes	*	0.0186
12h vs. 18h	-0.6279	-1.566 to 0.3097	No	ns	0.2968

Figure 2 - Amplitude

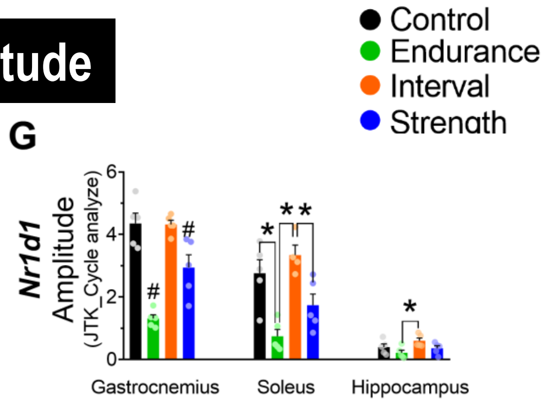

JTK_CYCLE OUTPUT

Gastrocnemius

Probeset	BH,Q	ADJ,P	PER	LAG	AMP	X0	X6	X12	X18
ct5	1	1	4	3	5,3830533	0,731733	0,510107	8,876606	7,590808
i4	1	1	4	3	4,656348	0,533797	0,393767	6,178143	7,919562
ct2	1	1	4	3	4,5666381	0,691725	0,45378	6,161297	7,900611
ct1	1	1	4	3	4,5413261	1,052853	0,604338	5,488524	9,013477
i3	1	1	4	3	4,5126636	0,725416	0,618025	4,872605	12,1973
i1	1	1	4	3	4,291553	0,576174	0,43904	4,485155	8,892398
i2	1	1	4	3	4,2874582	0,567488	0,489577	6,350811	6,833017
i5	1	1	4	3	3,8638492	0,477995	0,510634	5,851758	6,065487
s4	1	1	4	3	3,84896	0,663298	0,364287	5,089493	6,824595
s5	1	1	4	3	3,7675321	0,495894	0,415877	4,303011	7,26495
ct3	1	1	4	3	3,7093701	1,367656	0,565382	4,906296	7,518425
ct4	1	1	4	3	3,5697798	1,156033	0,41693	5,739103	5,930722
s3	1	1	4	3	3,0222154	0,700147	0,41693	3,460729	6,204464
s1	1	1	4	3	2,3607453	1,602443	0,349547	2,575279	6,991998
e3	1	1	4	3	1,7320326	0,699095	0,292693	1,925669	4,942093
s2	1	1	4	3	1,7163974	1,192883	0,552748	2,170983	9,038745
e4	1	1	4	3	1,3534639	0,750684	0,234786	1,510844	7,526848
e5	1	1	4	3	1,3325254	0,979154	0,214782	1,471099	6,241314
e2	1	1	4	3	1,1066684	1,070752	0,225311	1,268688	6,868814
e1	1	1	4	3	1,0229155	1,124447	0,214782	1,179196	5,627501

JTK_CYCLE OUTPUT

Soleus

Probeset	BH,Q	ADJ,P	PER	LAG	AMP	X0	X6	X12	X18
i3	1	1	4	3	4,2392505	0,450107	0,41282	5,355114	7,498224
ct2	1	1	4	3	3,7853816	0,976563	0,369318	4,533026	7,519531
ct5	1	1	4	3	3,3380255	0,954368	0,3855203	5,167791	5,613459
i5	1	1	4	3	3,2743083	0,5134353	0,332031	3,4190785	10,28498
i4	1	1	4	3	3,123934	0,5134353	0,521129	4,108665	5,761719
ct4	1	1	4	3	2,8945925	0,995206	0,528232	3,25728	8,876953
i2	1	1	4	3	2,7345145	0,611683	0,370206	3,78196	4,934304
s5	1	1	4	3	2,7222734	0,391513	0,350675	2,917259	6,792436
ct3	1	1	4	3	2,5339458	1,18608	0,29652	2,685547	8,108132
s4	1	1	4	3	2,4416652	0,587713	0,366655	2,668679	9,892578
e1	1	1	4	0	1,4312888	1,789773	0,440341	0,941051	8,592862
s1	1	1	4	0	1,4181058	1,633523	0,29652	1,057351	6,954013
ct1	1	1	4	3	1,2523777	0,887784	0,348011	1,528764	8,18892
s3	1	1	4	3	1,2467275	0,482955	0,608132	1,658381	10,64187
e2	1	1	4	0	1,0659338	1,419567	0,414595	0,879794	8,362926
s2	1	1	4	3	0,8597149	1,058239	0,390625	1,201172	9,443359
e4	1	1	4	0	0,4849423	0,927734	0,470526	0,753729	7,674893
e5	1	1	4	3	0,415262	0,924183	0,5699575	0,9614703	8,2750353
e3	1	1	4	3	0,3361646	1,020064	0,954368	1,271307	8,46946

JTK_CYCLE OUTPUT

Hippocampus

Probeset	BH,Q	ADJ,P	PER	LAG	AMP	X0	X6	X12	X18
i3	1	1	4	3	0,8602827	1,090909	0,274286	2,539221	1,259221
i5	1	1	4	0	0,7633076	1,167792	0,164156	0,968312	2,123636
ct4	1	1	4	3	0,7273095	0,604675	0,22026	0,905974	2,649351
s2	1	1	4	3	0,5576039	0,592208	0,409351	0,935065	1,967792
e2	1	1	4	1	0,5105863	0,903896	1,419221	0,407273	0,471688
i2	1	1	4	3	0,4760572	0,527792	0,322078	0,770909	3,962597
ct1	1	1	4	0	0,4716491	2,077922	0,702338	0,428052	0,872727
i1	1	1	4	3	0,4635682	2,115325	0,521558	2,491429	1,456623
s5	1	1	4	0	0,4569564	0,966234	0,232727	0,831169	1,39013
i4	1	1	4	3	0,4429985	0,937143	0,847792	2,09039	1,265455
s4	1	1	4	3	0,4297742	0,881039	0,554805	0,96	2,055065
ct5	1	1	4	0	0,4113495	1,13178	0,54597	0,658701	1,236363
e3	1	1	4	3	0,2857814	0,463377	0,442597	0,783377	0,930909
s1	1	1	4	3	0,2725578	0,442597	0,182857	0,511169	0,885195
ct2	1	1	4	1	0,2270089	1,065974	0,893506	0,401039	0,851948
ct3	1	1	4	3	0,157951	0,251429	0,367792	0,494545	0,571429
e1	1	1	4	3	0,1469315	0,552727	0,361558	0,637922	0,691948
e5	1	1	4	1	0,0934232	0,57922	0,741125	0,5698703	0,486234
s3	1	1	4	0	0,0837508	0,976623	0,527792	0,901818	0,980779
e4	1	1	4	2	0,0563236	0,396883	0,7411253	0,450909	0,635844

Figure 2 - Amplitude

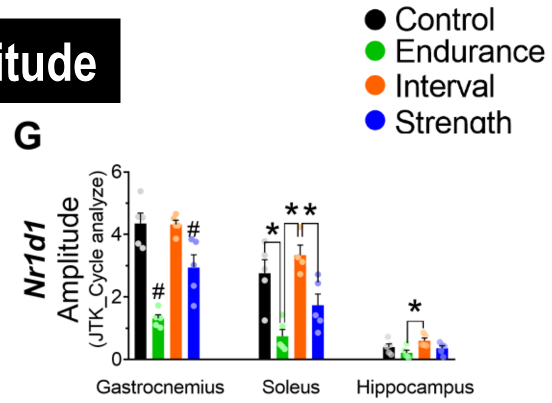

Gastrocnemius

ANOVA summary	
F	26.85
P value	<0.0001
P value summary	****
Significant diff. among means (P < 0.05)?	Yes
R square	0.8343

Tukey's multiple comparisons test	Mean Diff.	95.00% CI of diff.	Significant?	Summary	Adjusted P Value
CT vs. END	3.045	1.920 to 4.170	Yes	****	<0.0001
CT vs. int	0.03166	-1.093 to 1.157	No	ns	0.9998
CT vs. STR	1.411	0.2859 to 2.536	Yes	*	0.0118
END vs. int	-3.013	-4.138 to -1.888	Yes	****	<0.0001
END vs. STR	-1.634	-2.759 to -0.5087	Yes	**	0.0037
int vs. STR	1.379	0.2542 to 2.504	Yes	*	0.0139

Soleus

ANOVA summary	
F	10.75
P value	0.0005
P value summary	***
Significant diff. among means (P < 0.05)?	Yes
R square	0.6826

Tukey's multiple comparisons test	Mean Diff.	95.00% CI of diff.	Significant?	Summary	Adjusted P Value
CT vs. END	2.014	0.6460 to 3.382	Yes	**	0.0035
CT vs. int	-0.5821	-2.033 to 0.8690	No	ns	0.6620
CT vs. STR	1.023	-0.3450 to 2.391	No	ns	0.1808
END vs. int	-2.596	-4.047 to -1.145	Yes	***	0.0006
END vs. STR	-0.9910	-2.359 to 0.3772	No	ns	0.2017
int vs. STR	1.605	0.1541 to 3.056	Yes	*	0.0279

Hippocampus

ANOVA summary	
F	3.179
P value	0.0526
P value summary	ns
Significant diff. among means (P < 0.05)?	No
R square	0.3735

Tukey's multiple comparisons test	Mean Diff.	95.00% CI of diff.	Significant?	Summary	Adjusted P Value
CT vs. END	0.1804	-0.1781 to 0.5390	No	ns	0.4942
CT vs. int	-0.2022	-0.5607 to 0.1563	No	ns	0.3992
CT vs. STR	0.03893	-0.3196 to 0.3974	No	ns	0.9892
END vs. int	-0.3826	-0.7412 to -0.02411	Yes	*	0.0344
END vs. STR	-0.1415	-0.5000 to 0.2170	No	ns	0.6774
int vs. STR	0.2411	-0.1174 to 0.5996	No	ns	0.2573

Figure 2 - Gastrocnemius

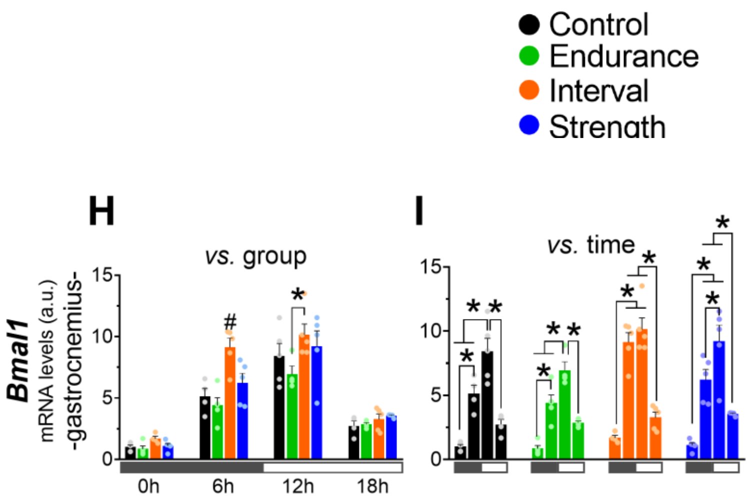

Gastrocnemius

	Control	Endurance	Interval	Strength
	1	1,209	2,585	0,691
	1,747	0,89	3,475	1,849
Time course - 0h	1,944	0,682	2,455	1,838
	0,987	2,661	2,049	1,693
	2,149	1,27		2,567
Mean	1,565	1,342	2,641	1,728
Std. Deviation	0,5411	0,775	0,601	0,6722
Std. Error of Mean	0,242	0,3466	0,3005	0,3006
Shapiro-Wilk test				
W	0,8541	0,824	0,9258	0,913
P value	0,208	0,1254	0,5699	0,4857
Passed normality test (alpha=0.05)?	Yes	Yes	Yes	Yes
P value summary	ns	ns	ns	ns

	Control	Endurance	Interval	Strength
	8,98	6,325	13,571	6,748
	10,273	4,01	10,135	6,95
Time course - 6h	7,309	7,048	15,405	11,813
	5,451	6,994	16,266	10,454
		10,062	16,149	12,668
Mean	8,003	6,888	14,31	9,727
Std. Deviation	2,09	2,163	2,568	2,744
Std. Error of Mean	1,045	0,9672	1,149	1,227
Shapiro-Wilk test				
W	0,9848	0,9414	0,8362	0,8665
P value	0,9293	0,6757	0,1548	0,2526
Passed normality test (alpha=0.05)?	Yes	Yes	Yes	Yes
P value summary	ns	ns	ns	ns

	Control	Endurance	Interval	Strength
	9,214	13,932	21,157	17,095
	18,055	9,966	15,858	13,961
Time course - 12h	13,246	10,242	14,998	18,062
	15,071	9,373	13,648	15,877
	10,239		13,685	7,165
Mean	13,17	10,88	15,87	14,43
Std. Deviation	3,596	2,068	3,099	4,341
Std. Error of Mean	1,608	1,034	1,386	1,941
Shapiro-Wilk test				
W	0,9588	0,7879	0,7916	0,8487
P value	0,7999	0,0822	0,0691	0,1905
Passed normality test (alpha=0.05)?	Yes	Yes	Yes	Yes
P value summary	ns	ns	ns	ns

	Control	Endurance	Interval	Strength
	5,312	3,897	6,218	5,68
	5,098	4,963	5,745	5,183
Time course - 18h	4,01	4,681	6,544	5,626
	2,627	4,282	3,346	5,659
			3,74	
Mean	4,262	4,456	5,119	5,537
Std. Deviation	1,23	0,4656	1,473	0,237
Std. Error of Mean	0,615	0,2328	0,6586	0,1185
Shapiro-Wilk test				
W	0,9047	0,9815	0,8551	0,7145
P value	0,455	0,9107	0,211	0,0169
Passed normality test (alpha=0.05)?	Yes	Yes	Yes	No
P value summary	ns	ns	ns	*

Figure 2 - Gastrocnemius

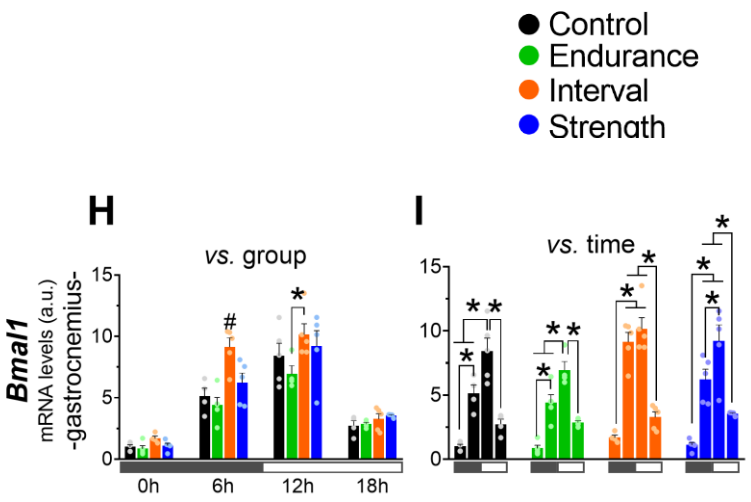

Two-way ANOVA	Ordinary
Alpha	0,05

Source of Variation	% of total variation	P value	P value summary	Significant?
Interaction	4,177	0,0609	ns	No
Row Factor	72,53	<0,0001	****	Yes
Column Factor	6,332	<0,0001	****	Yes

ANOVA table	SS (Type III)	DF	MS	F (DFn, DFd)	P value
Interaction	35,65	9	3,961	F (9, 58) = 1,961	P=0,0609
Row Factor	619	3	206,3	F (3, 58) = 102,1	P<0,0001
Column Factor	54,04	3	18,01	F (3, 58) = 8,916	P<0,0001
Residual	117,2	58	2,021		

Tukey's multiple comparisons test	Mean Diff.	95.00% CI of diff.	Significant?	Summary	Adjusted P Value
0h					
Control vs. Endurance	0,1425	-2,236 to 2,520	No	ns	0,9986
Control vs. Interval	-0,6871	-3,209 to 1,835	No	ns	0,8885
Control vs. Strength	-0,1036	-2,482 to 2,274	No	ns	0,9994
Endurance vs. Interval	-0,8296	-3,352 to 1,693	No	ns	0,8203
Endurance vs. Strength	-0,2461	-2,624 to 2,132	No	ns	0,9928
Interval vs. Strength	0,5835	-1,939 to 3,106	No	ns	0,9279
6h					
Control vs. Endurance	0,7126	-1,810 to 3,235	No	ns	0,8775
Control vs. Interval	-4,026	-6,548 to -1,504	Yes	***	0,0005
Control vs. Strength	-1,101	-3,623 to 1,421	No	ns	0,6576
Endurance vs. Interval	-4,738	-7,116 to -2,360	Yes	****	<0,0001
Endurance vs. Strength	-1,813	-4,191 to 0,5645	No	ns	0,1936
Interval vs. Strength	2,925	0,5469 to 5,303	Yes	*	0,01
12h					
Control vs. Endurance	1,461	-1,061 to 3,983	No	ns	0,4254
Control vs. Interval	-1,727	-4,105 to 0,6505	No	ns	0,2304
Control vs. Strength	-0,8094	-3,187 to 1,569	No	ns	0,8047
Endurance vs. Interval	-3,188	-5,711 to -0,6661	Yes	**	0,0077
Endurance vs. Strength	-2,27	-4,792 to 0,2520	No	ns	0,0922
Interval vs. Strength	0,9181	-1,460 to 3,296	No	ns	0,7377
18h					
Control vs. Endurance	-0,1239	-2,783 to 2,535	No	ns	0,9993
Control vs. Interval	-0,5474	-3,070 to 1,975	No	ns	0,9394
Control vs. Strength	-0,8146	-3,473 to 1,844	No	ns	0,8493
Endurance vs. Interval	-0,4234	-2,946 to 2,099	No	ns	0,9705
Endurance vs. Strength	-0,6907	-3,349 to 1,968	No	ns	0,9016
Interval vs. Strength	-0,2673	-2,789 to 2,255	No	ns	0,9922

Figure 2 - Gastrocnemius

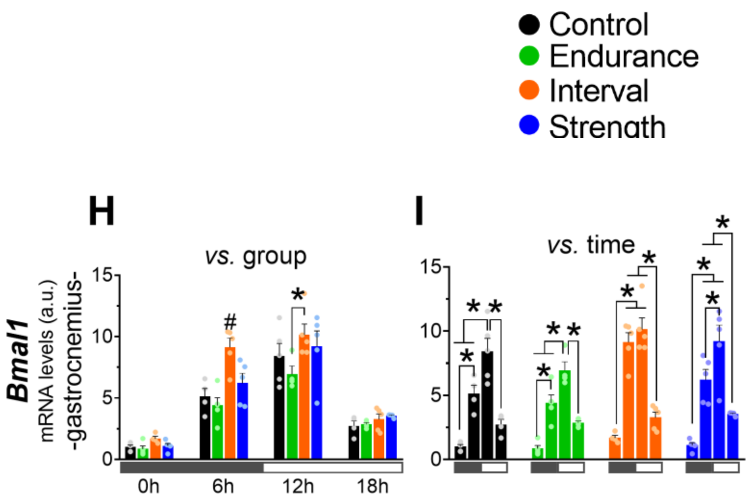

Two-way ANOVA	Ordinary
Alpha	0,05

Source of Variation	% of total variation	P value	P value summary	Significant?
Interaction	4,177	0,0609	ns	No
Row Factor	72,53	<0,0001	****	Yes
Column Factor	6,332	<0,0001	****	Yes

ANOVA table	SS (Type III)	DF	MS	F (DFn, DFd)	P value
Interaction	35,65	9	3,961	F (9, 58) = 1,961	P=0,0609
Row Factor	619	3	206,3	F (3, 58) = 102,1	P<0,0001
Column Factor	54,04	3	18,01	F (3, 58) = 8,916	P<0,0001
Residual	117,2	58	2,021		

Tukey's multiple comparisons test	Mean Diff.	95.00% CI of diff.	Significant?	Summary	Adjusted P Value
Control					
0h vs. 6h	-4,113	-6,635 to -1,590	Yes	***	0,0004
0h vs. 12h	-7,41	-9,788 to -5,032	Yes	****	<0,0001
0h vs. 18h	-1,722	-4,245 to 0,7997	No	ns	0,2808
6h vs. 12h	-3,297	-5,820 to -0,7752	Yes	**	0,0055
6h vs. 18h	2,39	-0,2685 to 5,049	No	ns	0,0928
12h vs. 18h	5,688	3,165 to 8,210	Yes	****	<0,0001
Endurance					
0h vs. 6h	-3,542	-5,920 to -1,165	Yes	**	0,0012
0h vs. 12h	-6,092	-8,614 to -3,569	Yes	****	<0,0001
0h vs. 18h	-1,989	-4,511 to 0,5334	No	ns	0,1699
6h vs. 12h	-2,549	-5,071 to -0,02694	Yes	*	0,0467
6h vs. 18h	1,554	-0,9686 to 4,076	No	ns	0,3705
12h vs. 18h	4,103	1,444 to 6,761	Yes	***	0,0008
Interval					
0h vs. 6h	-7,451	-9,973 to -4,929	Yes	****	<0,0001
0h vs. 12h	-8,45	-10,97 to -5,928	Yes	****	<0,0001
0h vs. 18h	-1,583	-4,105 to 0,9395	No	ns	0,3541
6h vs. 12h	-0,9991	-3,377 to 1,379	No	ns	0,6841
6h vs. 18h	5,869	3,491 to 8,246	Yes	****	<0,0001
12h vs. 18h	6,868	4,490 to 9,246	Yes	****	<0,0001
Strength					
0h vs. 6h	-5,11	-7,488 to -2,732	Yes	****	<0,0001
0h vs. 12h	-8,116	-10,49 to -5,738	Yes	****	<0,0001
0h vs. 18h	-2,433	-4,956 to 0,08872	No	ns	0,0624
6h vs. 12h	-3,006	-5,384 to -0,6279	Yes	**	0,0077
6h vs. 18h	2,676	0,1542 to 5,199	Yes	*	0,0335
12h vs. 18h	5,682	3,160 to 8,204	Yes	****	<0,0001

Figure 2 - Gastrocnemius

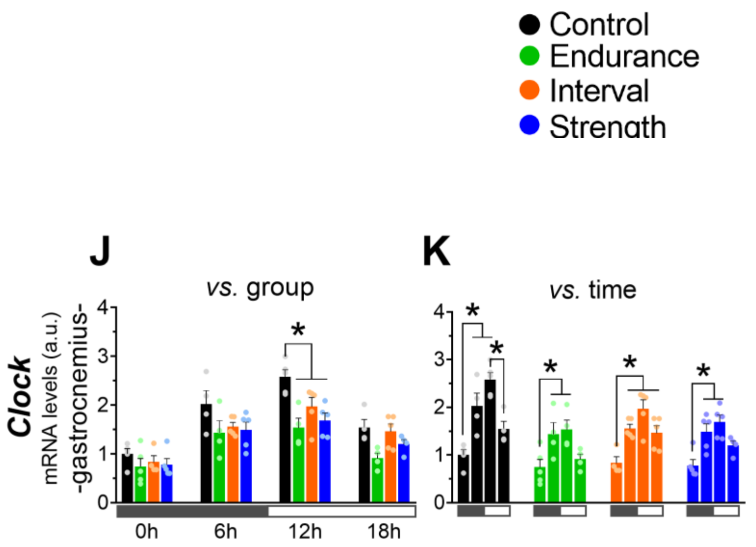

Gastrocnemius

	Control	Endurance	Interval	Strength
	1	0,645	0,762	0,689
	1,109	0,388	1,218	1,261
Time course - 0h	1,084	0,939	0,678	0,607
	0,622	0,477	0,68	0,752
	1,212	1,274		0,603
Mean	1,005	0,7446	0,8345	0,7824
Std. Deviation	0,2273	0,3629	0,2586	0,2746
Std. Error of Mean	0,1016	0,1623	0,1293	0,1228
Shapiro-Wilk test				
W	0,8474	0,9312	0,7377	0,7396
P value	0,1865	0,6049	0,0294	0,0238
Passed normality test (alpha=0.05)?	Yes	Yes	No	No
P value summary	ns	ns	*	*

	Control	Endurance	Interval	Strength
	1,402	0,916	1,425	1,187
	2,689	2,084	1,764	1,006
Time course - 6h	1,817	1,481	1,466	1,694
	2,19	1,263	1,366	1,706
			1,767	1,851
Mean	2,025	1,436	1,558	1,489
Std. Deviation	0,5476	0,4907	0,1931	0,369
Std. Error of Mean	0,2738	0,2453	0,08635	0,165
Shapiro-Wilk test				
W	0,9961	0,9746	0,8136	0,8663
P value	0,986	0,8696	0,1042	0,2516
Passed normality test (alpha=0.05)?	Yes	Yes	Yes	Yes
P value summary	ns	ns	ns	ns

	Control	Endurance	Interval	Strength
	2,734	2,059	2,238	1,361
	2,997	1,251	2,204	1,852
Time course - 12h	2,225	1,614	2,264	1,804
	2,659	1,218	1,87	2,09
	2,263		1,289	1,341
Mean	2,576	1,536	1,973	1,69
Std. Deviation	0,328	0,3924	0,4143	0,3276
Std. Error of Mean	0,1467	0,1962	0,1853	0,1465
Shapiro-Wilk test				
W	0,9131	0,8821	0,7906	0,8849
P value	0,4862	0,3479	0,0678	0,3319
Passed normality test (alpha=0.05)?	Yes	Yes	Yes	Yes
P value summary	ns	ns	ns	ns

	Control	Endurance	Interval	Strength
	1,337	0,649	1,585	1,365
	2,017	0,845	1,766	1,279
Time course - 18h	1,313	1,02	1,763	0,934
	1,51	1,134	1,08	1,222
			1,123	
Mean	1,544	0,912	1,463	1,2
Std. Deviation	0,3272	0,2118	0,3387	0,1868
Std. Error of Mean	0,1636	0,1059	0,1515	0,09341
Shapiro-Wilk test				
W	0,8184	0,9759	0,8118	0,8925
P value	0,1394	0,8774	0,1008	0,3947
Passed normality test (alpha=0.05)?	Yes	Yes	Yes	Yes
P value summary	ns	ns	ns	ns

Figure 2 - Gastrocnemius

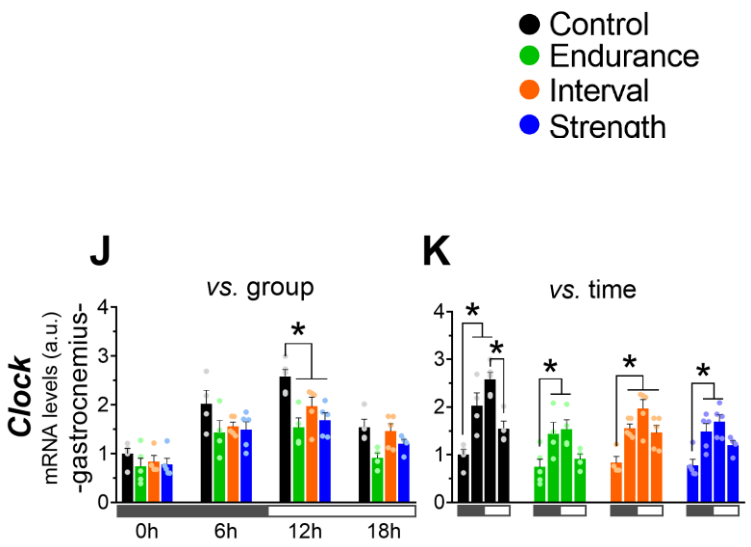

Two-way ANOVA	Ordinary
Alpha	0,05

Source of Variation	% of total variation	P value	P value summary	Significant?
Interaction	4,755	0,3594	ns	No
Row Factor	50,94	<0,0001	****	Yes
Column Factor	15,87	<0,0001	****	Yes

ANOVA table	SS (Type III)	DF	MS	F (DFn, DFd)	P value
Interaction	1,171	9	0,1301	F (9, 57) = 1,127	P=0,3594
Row Factor	12,54	3	4,18	F (3, 57) = 36,22	P<0,0001
Column Factor	3,906	3	1,302	F (3, 57) = 11,28	P<0,0001
Residual	6,579	57	0,1154		

Tukey's multiple comparisons test	Mean Diff.	95.00% CI of diff.	Significant?	Summary	Adjusted P Value
0h					
Control vs. Endurance	0,2608	-0,3078 to 0,8294	No	ns	0,6208
Control vs. Interval	0,1709	-0,4322 to 0,7740	No	ns	0,8763
Control vs. Strength	0,223	-0,3456 to 0,7916	No	ns	0,7281
Endurance vs. Interval	-0,0899	-0,6930 to 0,5132	No	ns	0,979
Endurance vs. Strength	-0,0378	-0,6064 to 0,5308	No	ns	0,998
Interval vs. Strength	0,0521	-0,5510 to 0,6552	No	ns	0,9957
6h					
Control vs. Endurance	0,5885	-0,04726 to 1,224	No	ns	0,0792
Control vs. Interval	0,4669	-0,1362 to 1,070	No	ns	0,1826
Control vs. Strength	0,5357	-0,06743 to 1,139	No	ns	0,0987
Endurance vs. Interval	-0,1216	-0,7247 to 0,4815	No	ns	0,9505
Endurance vs. Strength	-0,0528	-0,6559 to 0,5503	No	ns	0,9956
Interval vs. Strength	0,0688	-0,4998 to 0,6374	No	ns	0,9885
12h					
Control vs. Endurance	1,04	0,4370 to 1,643	Yes	***	0,0002
Control vs. Interval	0,6026	0,03396 to 1,171	Yes	*	0,0338
Control vs. Strength	0,886	0,3174 to 1,455	Yes	***	0,0007
Endurance vs. Interval	-0,4375	-1,041 to 0,1656	No	ns	0,2314
Endurance vs. Strength	-0,1541	-0,7572 to 0,4490	No	ns	0,9057
Interval vs. Strength	0,2834	-0,2852 to 0,8520	No	ns	0,5549
18h					
Control vs. Endurance	0,6323	-0,003508 to 1,268	No	ns	0,0518
Control vs. Interval	0,08085	-0,5223 to 0,6840	No	ns	0,9845
Control vs. Strength	0,3443	-0,2915 to 0,9800	No	ns	0,4844
Endurance vs. Interval	-0,5514	-1,155 to 0,05173	No	ns	0,0848
Endurance vs. Strength	-0,288	-0,9238 to 0,3478	No	ns	0,6301
Interval vs. Strength	0,2634	-0,3397 to 0,8665	No	ns	0,6568

Figure 2 - Gastrocnemius

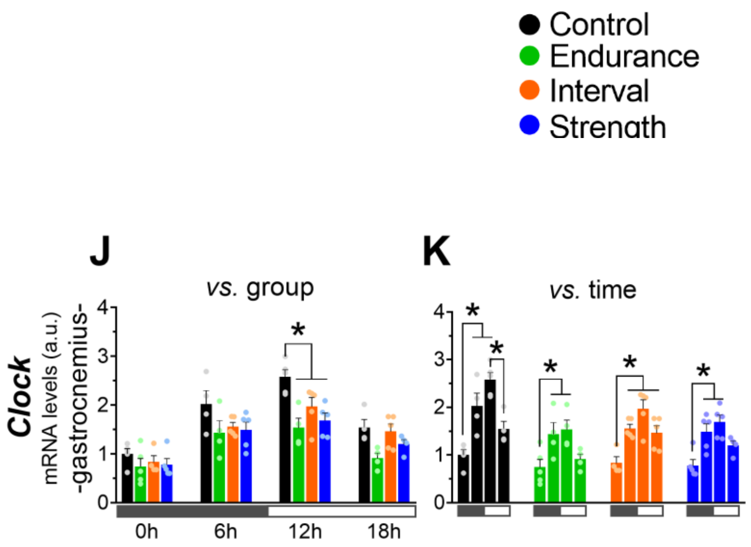

Two-way ANOVA	Ordinary
Alpha	0,05

Source of Variation	% of total variation	P value	P value summary	Significant?
Interaction	4,755	0,3594	ns	No
Row Factor	50,94	<0,0001	****	Yes
Column Factor	15,87	<0,0001	****	Yes

ANOVA table	SS (Type III)	DF	MS	F (DFn, DFd)	P value
Interaction	1,171	9	0,1301	F (9, 57) = 1,127	P=0,3594
Row Factor	12,54	3	4,18	F (3, 57) = 36,22	P<0,0001
Column Factor	3,906	3	1,302	F (3, 57) = 11,28	P<0,0001
Residual	6,579	57	0,1154		

Tukey's multiple comparisons test	Mean Diff.	95.00% CI of diff.	Significant?	Summary	Adjusted P Value
Control					
0h vs. 6h	-1,019	-1,622 to -0,4160	Yes	***	0,0002
0h vs. 12h	-1,57	-2,139 to -1,002	Yes	****	<0,0001
0h vs. 18h	-0,5388	-1,142 to 0,06428	No	ns	0,0958
6h vs. 12h	-0,5511	-1,154 to 0,05203	No	ns	0,085
6h vs. 18h	0,4803	-0,1555 to 1,116	No	ns	0,2004
12h vs. 18h	1,031	0,4282 to 1,634	Yes	***	0,0002
Endurance					
0h vs. 6h	-0,6914	-1,295 to -0,08827	Yes	*	0,0185
0h vs. 12h	-0,7909	-1,394 to -0,1878	Yes	**	0,0054
0h vs. 18h	-0,1674	-0,7705 to 0,4357	No	ns	0,8828
6h vs. 12h	-0,0995	-0,7353 to 0,5363	No	ns	0,9758
6h vs. 18h	0,524	-0,1118 to 1,160	No	ns	0,1408
12h vs. 18h	0,6235	-0,01226 to 1,259	No	ns	0,0565
Interval					
0h vs. 6h	-0,7231	-1,326 to -0,1200	Yes	*	0,0126
0h vs. 12h	-1,139	-1,742 to -0,5354	Yes	****	<0,0001
0h vs. 18h	-0,6289	-1,232 to -0,02577	Yes	*	0,0378
6h vs. 12h	-0,4154	-0,9840 to 0,1532	No	ns	0,2259
6h vs. 18h	0,0942	-0,4744 to 0,6628	No	ns	0,9716
12h vs. 18h	0,5096	-0,05904 to 1,078	No	ns	0,0943
Strength					
0h vs. 6h	-0,7064	-1,275 to -0,1378	Yes	**	0,0091
0h vs. 12h	-0,9072	-1,476 to -0,3386	Yes	***	0,0005
0h vs. 18h	-0,4176	-1,021 to 0,1855	No	ns	0,2691
6h vs. 12h	-0,2008	-0,7694 to 0,3678	No	ns	0,7865
6h vs. 18h	0,2888	-0,3143 to 0,8919	No	ns	0,5873
12h vs. 18h	0,4896	-0,1135 to 1,093	No	ns	0,1505

Figure 3 - Gastrocnemius

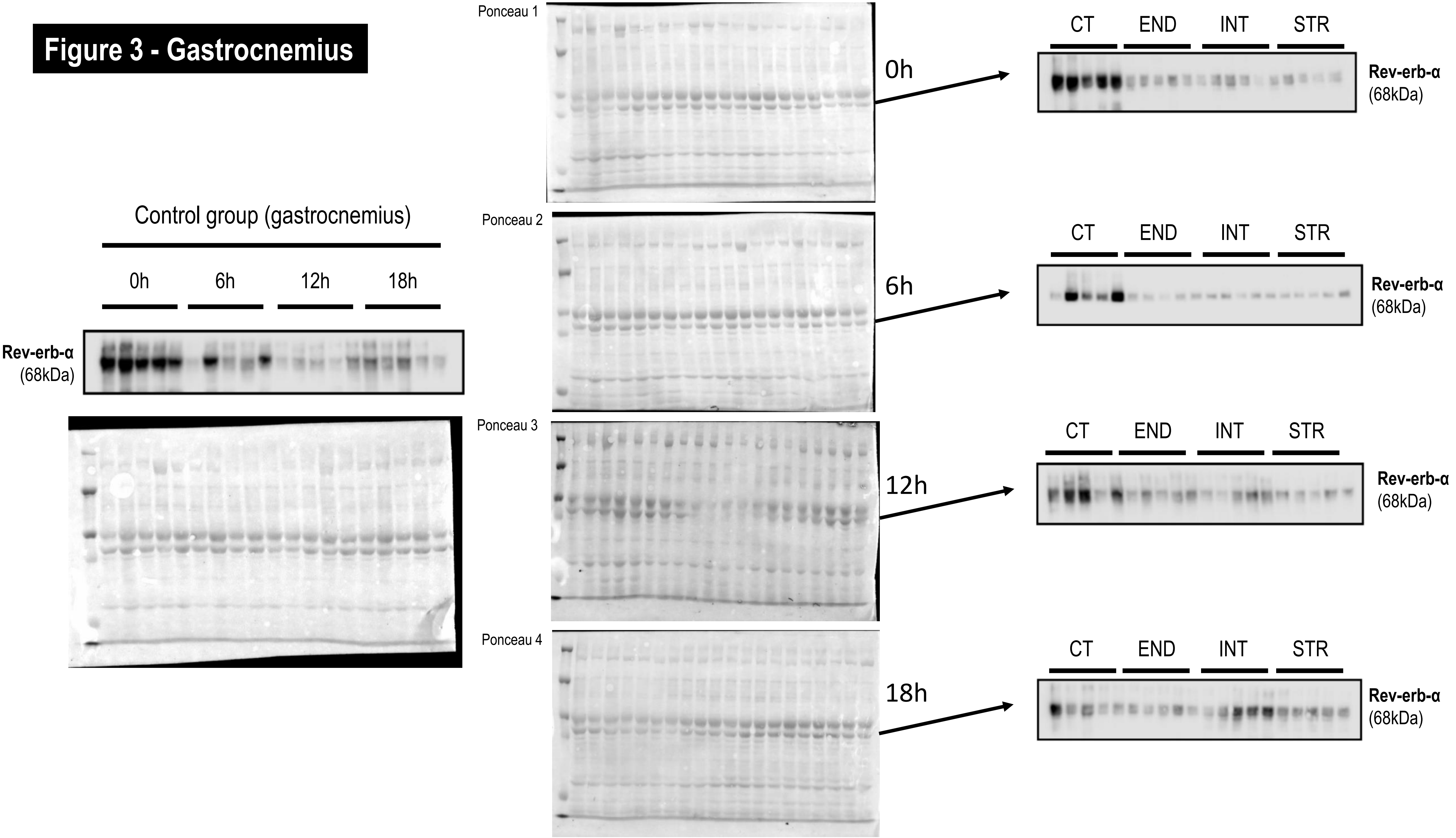

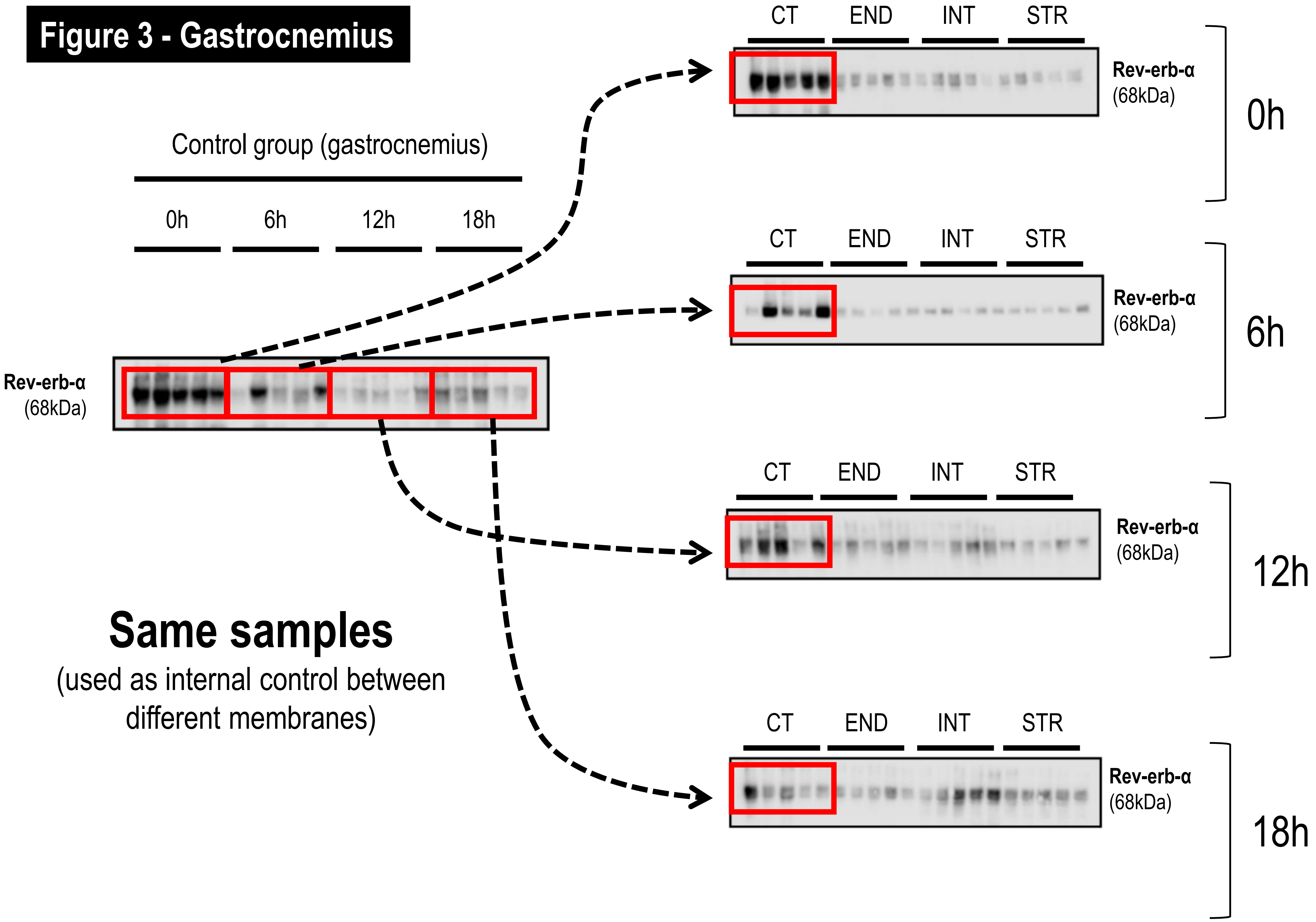

Figure 3 - Gastrocnemius
Control group (gastrocnemius)
0h
6h
12h
18h
Rev-erb-α
(68kDa)
Same samples
(used as internal control between different membranes)
CT
END
INT
STR
Rev-erb-α
(68kDa)
0h
CT
END
INT
STR
Rev-erb-α
(68kDa)
6h
CT
END
INT
STR
Rev-erb-α
(68kDa)
12h
CT
END
INT
STR
Rev-erb-α
(68kDa)
18h

Figure 3 - Gastrocnemius

Ponceau 1

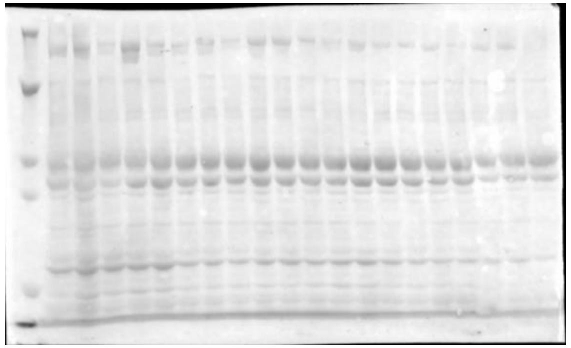

PONCEAU 1 quantification

CT	END	INT	STR
49423	39564	30641	38338
57777	38328	35615	48580
39332	41805	43594	30285
44268	52423	40613	42516
28225	48932	42601	23899

ANOVA summary	
F	0.9826
P value	0.4257
P value summary	ns
Significant diff. among means (P < 0.05)?	No
R square	0.1556

Ponceau 3

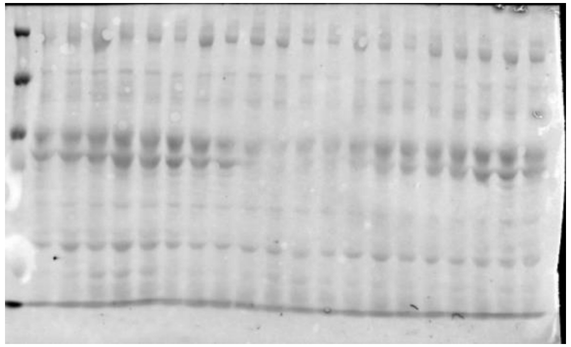

PONCEAU 3 quantification

CT	END	INT	STR
55838	42834	31844	36046
58034	65889	20594	52937
55400	39984	39352	57127
51855	46886	57342	52993
63197	39290	56112	63586

ANOVA summary	
F	1.914
P value	0.1679
P value summary	ns
Significant diff. among means (P < 0.05)?	No
R square	0.2641

Ponceau 2

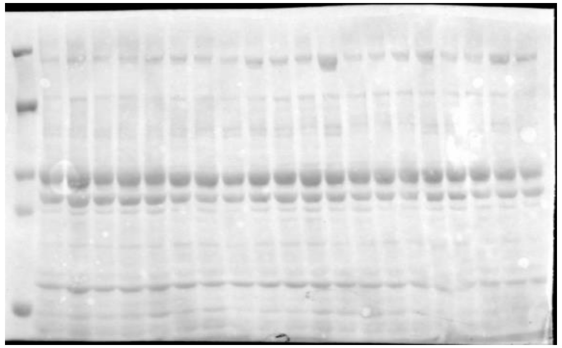

PONCEAU 2 quantification

CT	END	INT	STR
48446	40346	43679	35995
67655	51235	51139	22580
41731	35571	49737	22962
57980	50750	31423	38531
33279	59164	24459	45190

ANOVA summary	
F	2.275
P value	0.1190
P value summary	ns
Significant diff. among means (P < 0.05)?	No
R square	0.2991

Ponceau 4

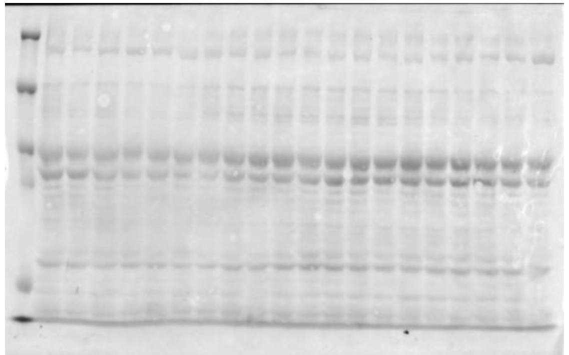

PONCEAU 4 quantification

CT	END	INT	STR
55338	16595	45319	27092
26801	35592	43712	17656
44928	40147	35561	28488
16856	28657	35824	24543
22510	53718	26069	28892

ANOVA summary	
F	1.012
P value	0.4132
P value summary	ns
Significant diff. among means (P < 0.05)?	No
R square	0.1595

Figure 3 - Gastrocnemius

Original images for Western Blot

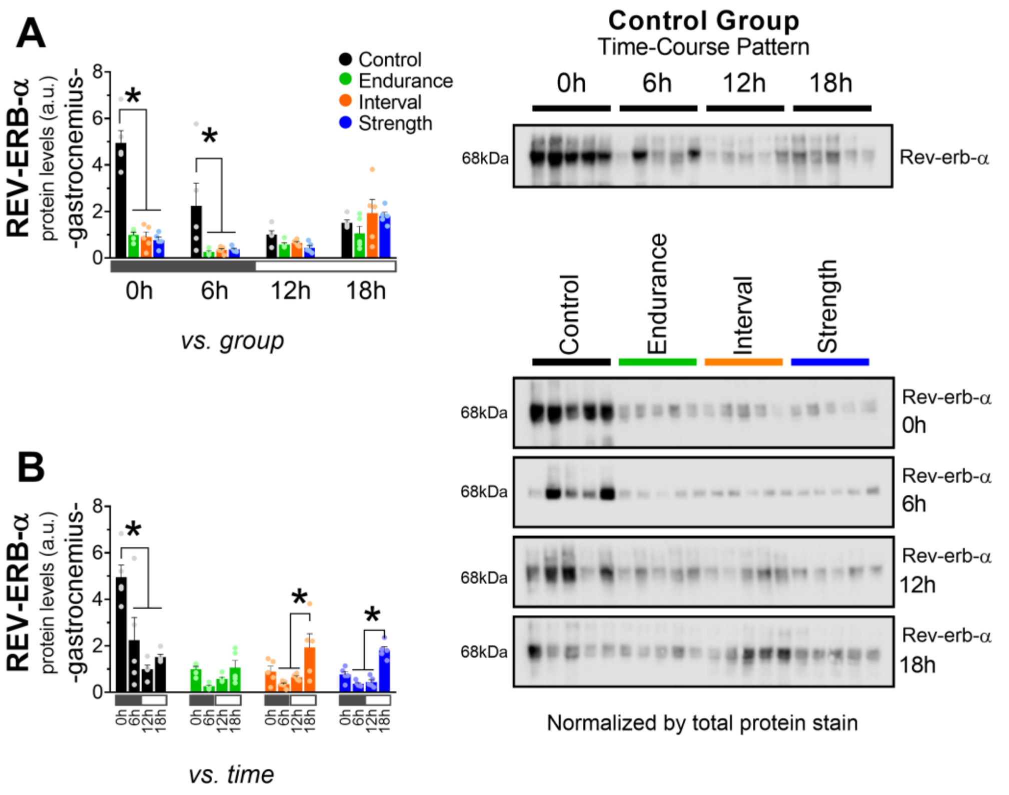

Membrane edges

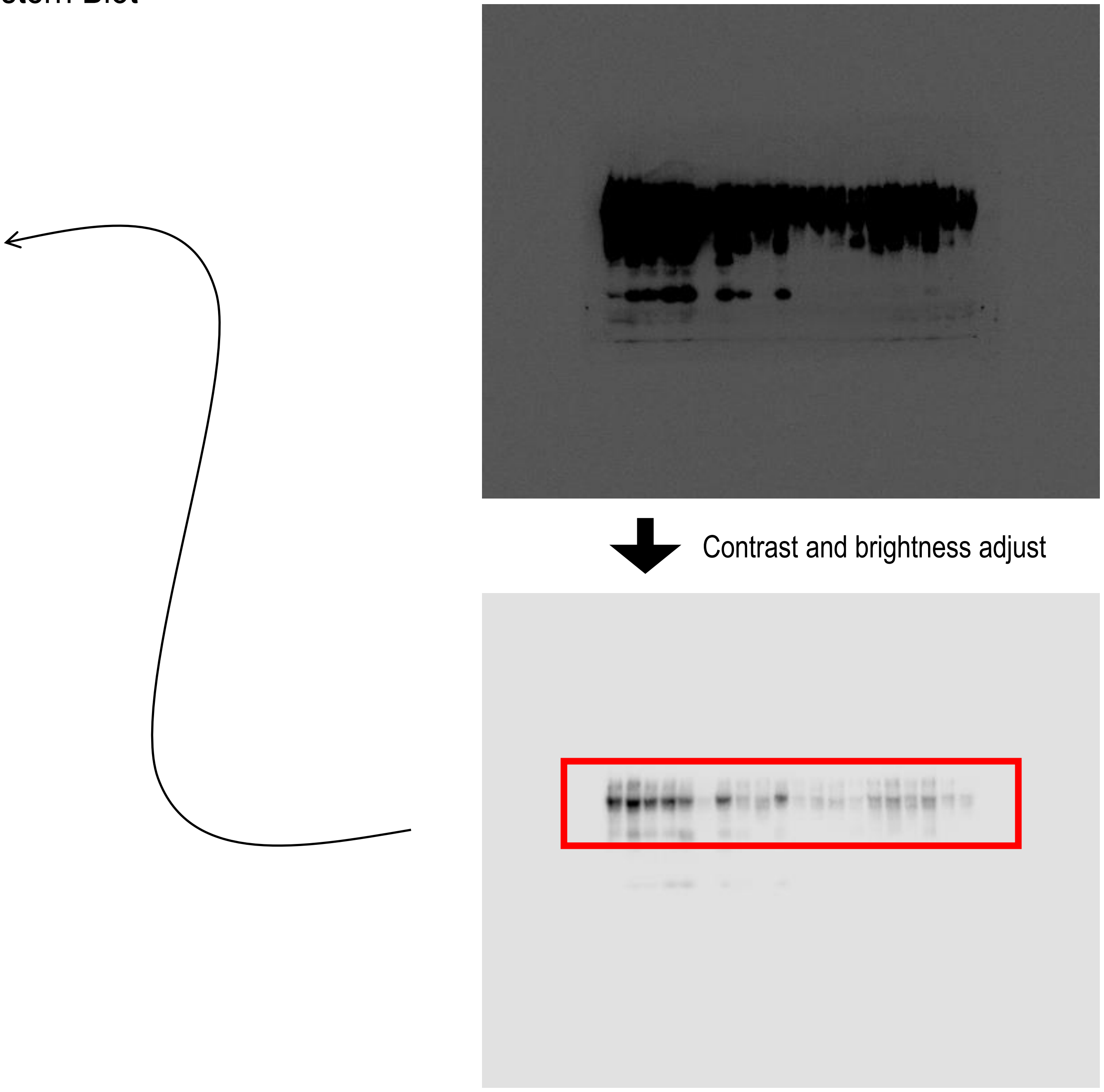

Figure 3 - Gastrocnemius

Original images for Western Blot

Membrane edges

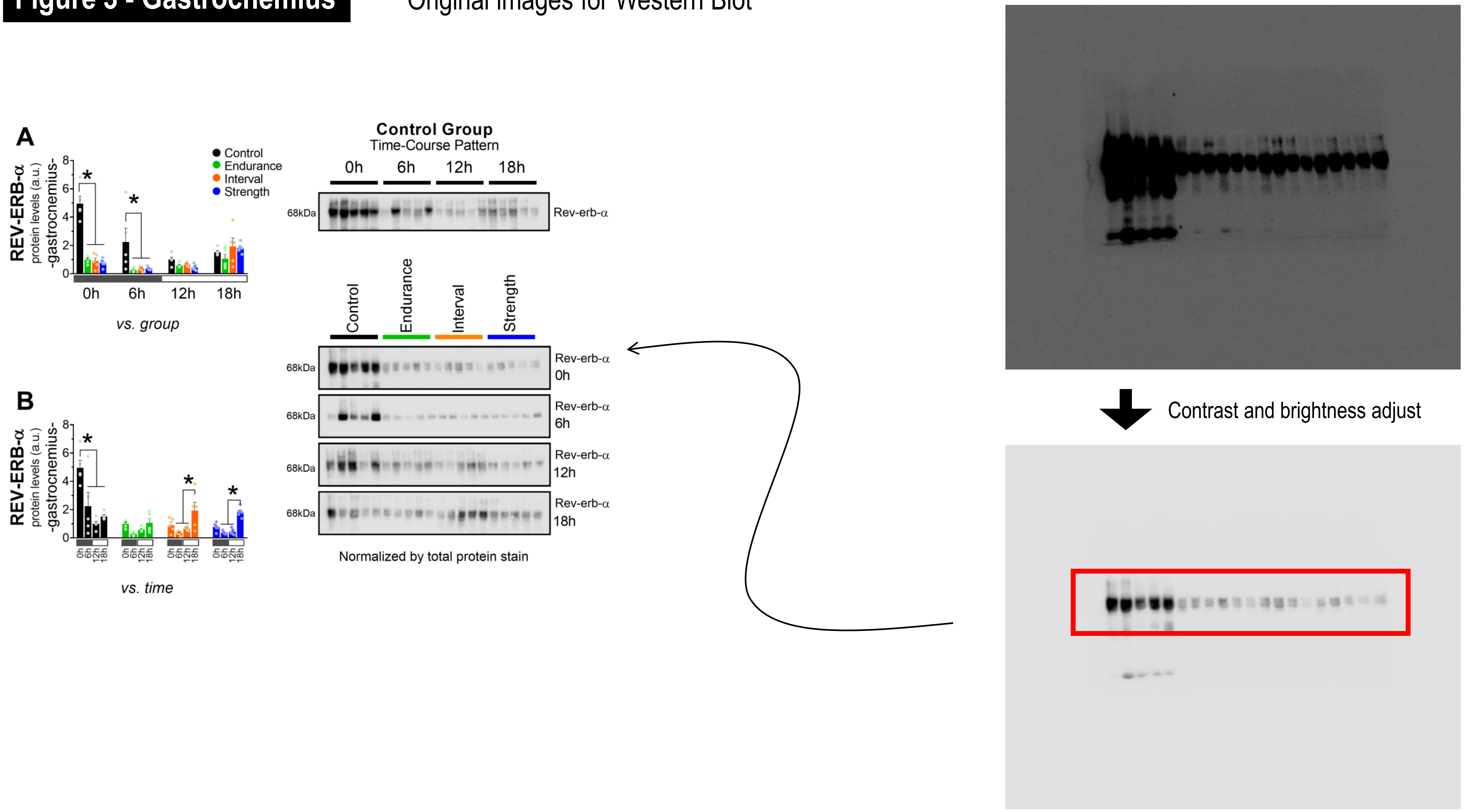

Figure 3 - Gastrocnemius

Original images for Western Blot

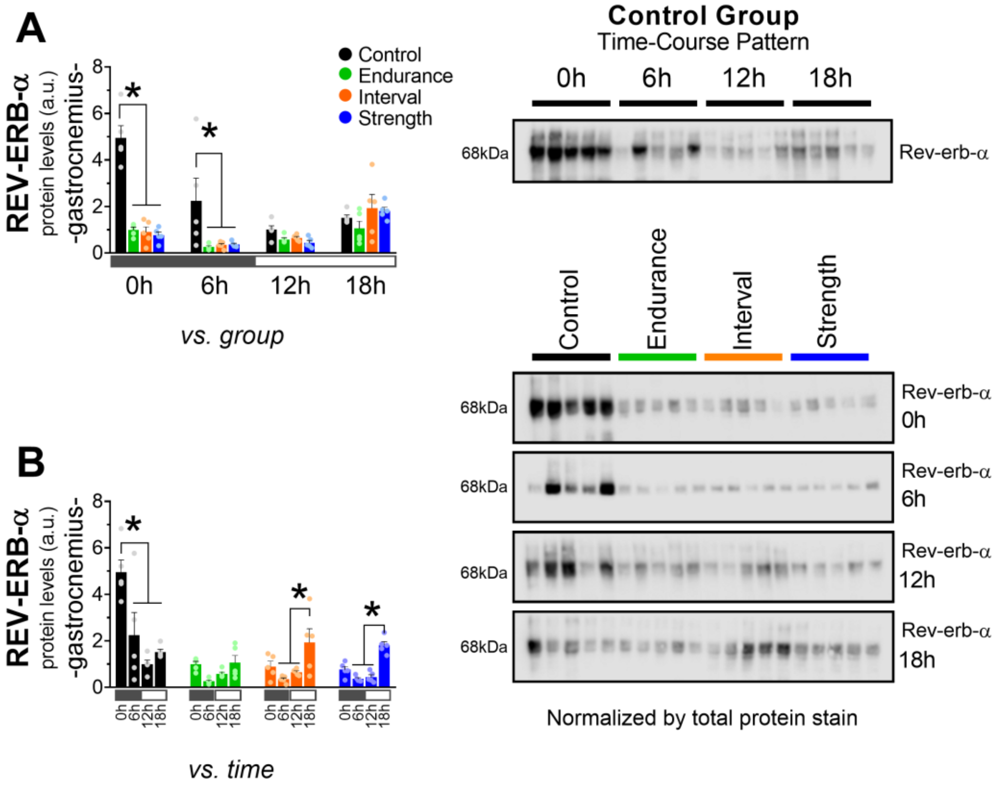

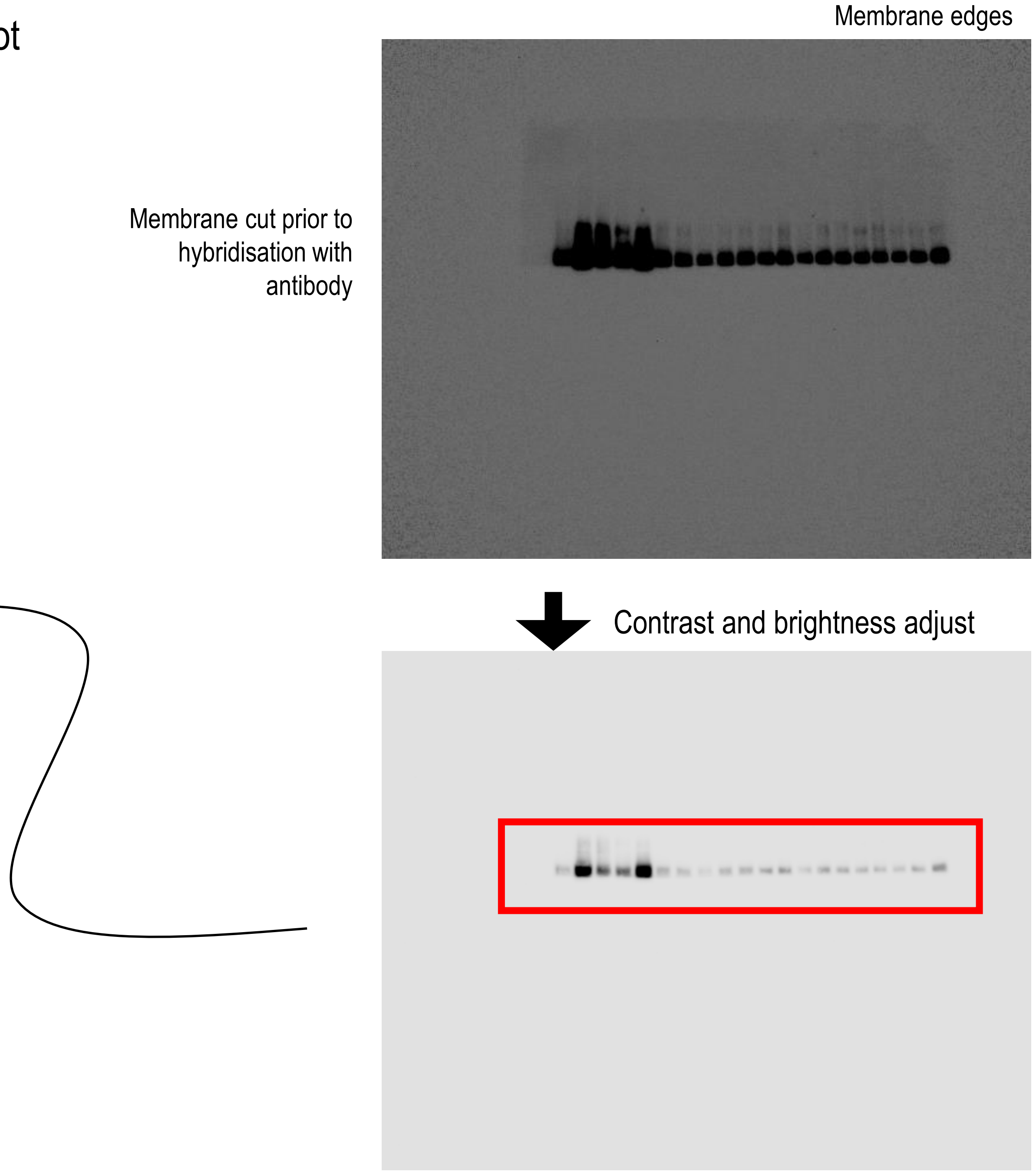

Figure 3 - Gastrocnemius

Original images for Western Blot

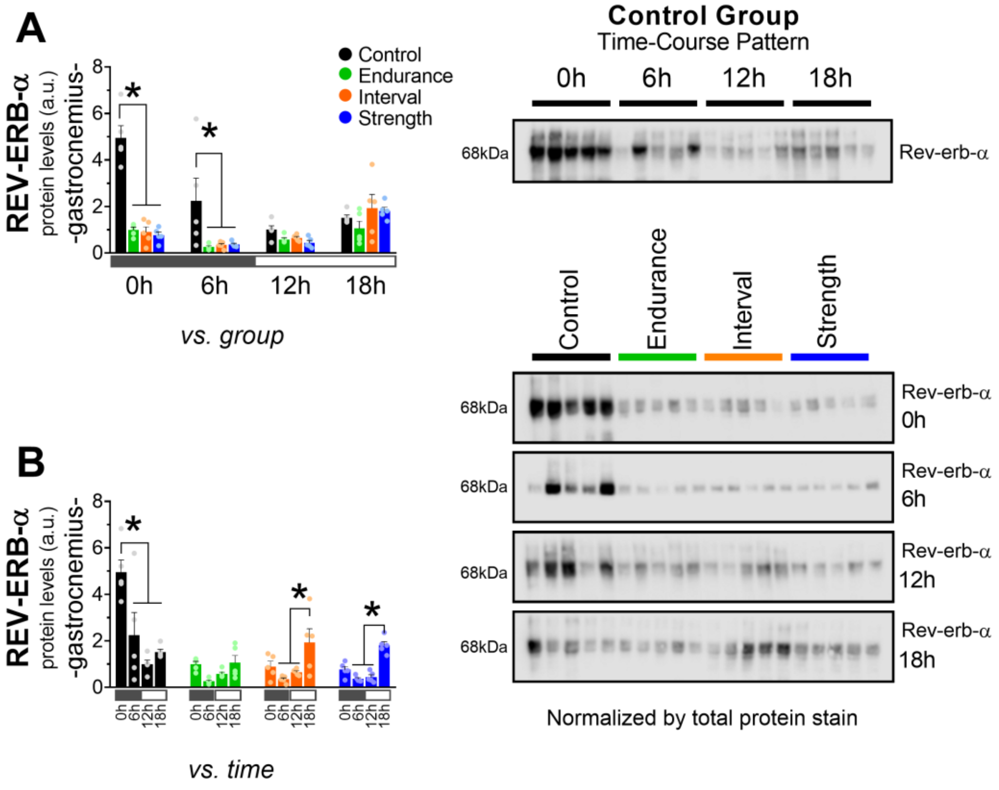

Membrane edges

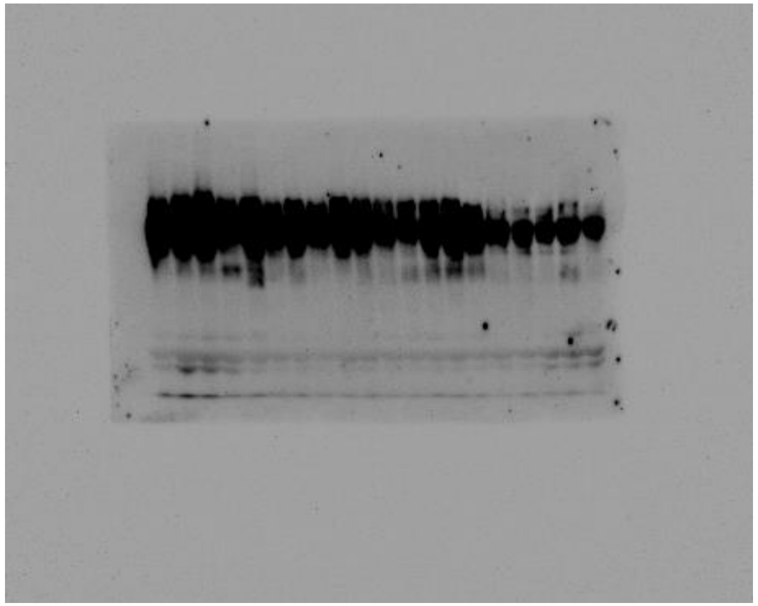

Contrast and brightness adjust

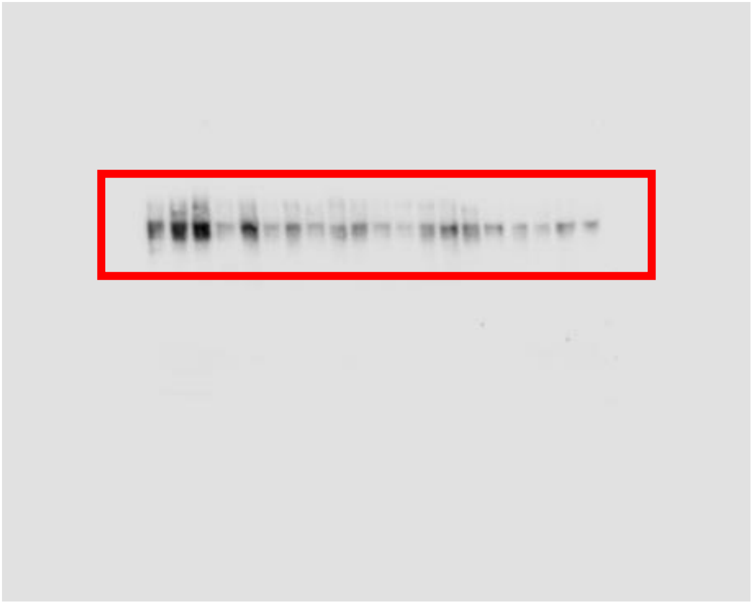

Figure 3 - Gastrocnemius

Original images for Western Blot

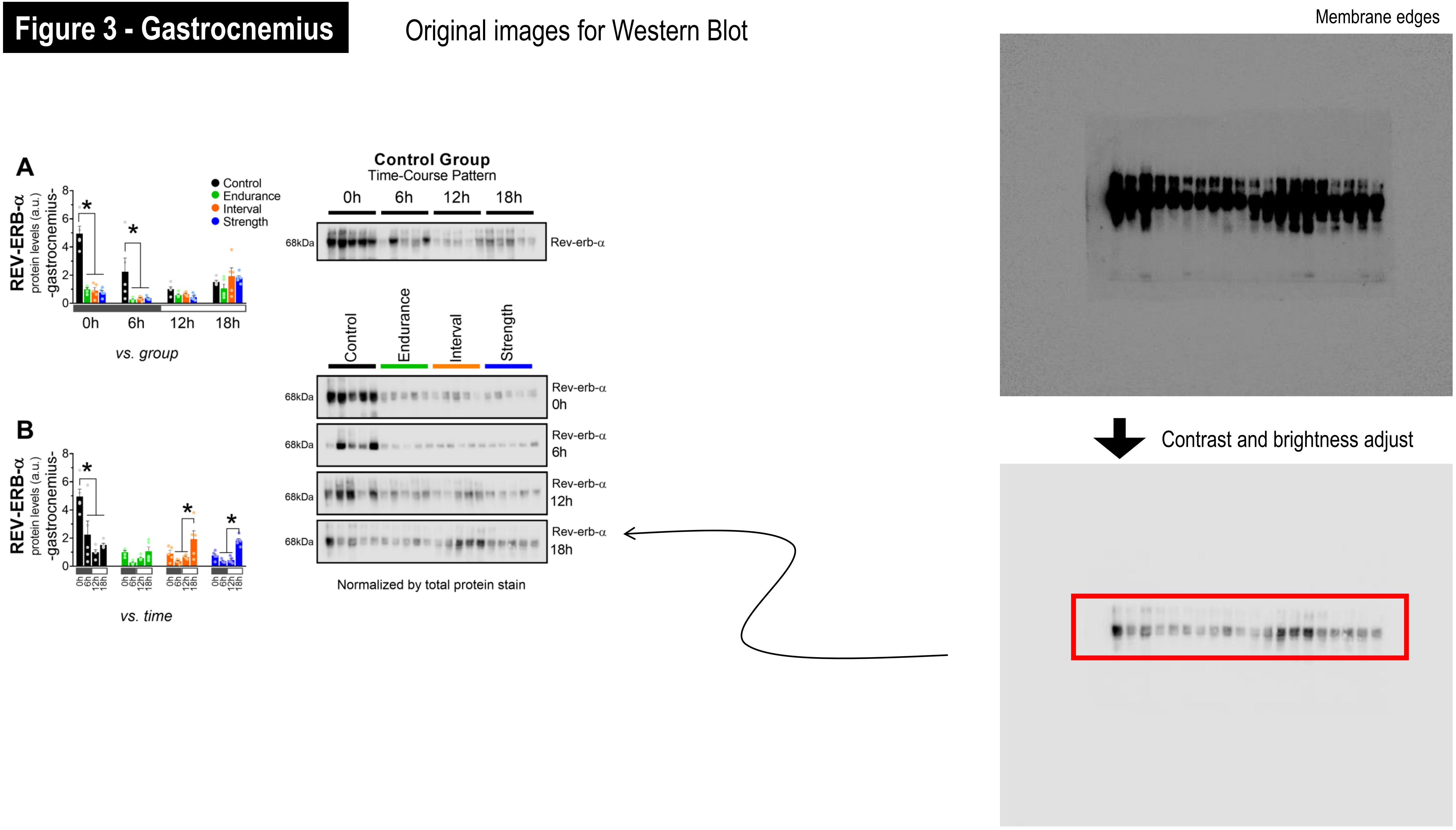

Figure 3 - Gastrocnemius

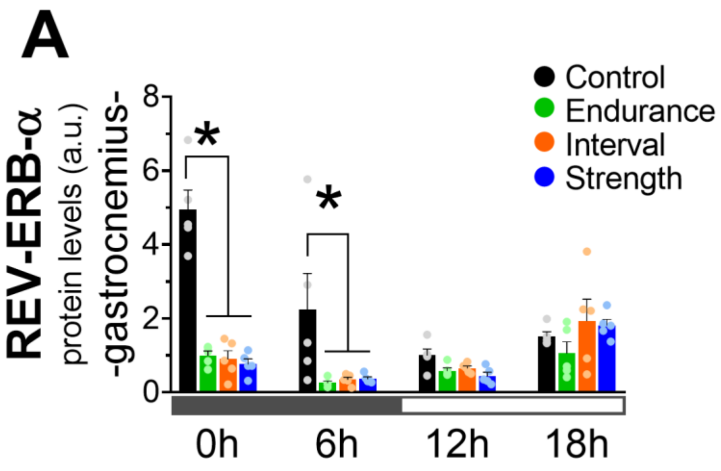

vs. group

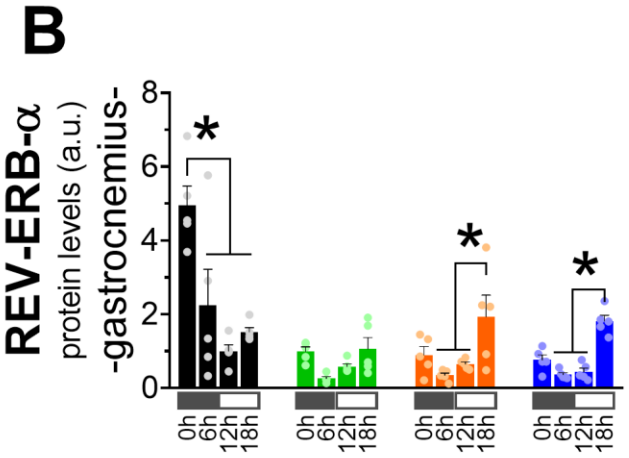

vs. time

Gastrocnemius

	Control	Endurance	Interval	Strength
Time course - 0h	5,213626	1,231002	0,64391	0,714626
	4,546796	1,202331	1,327361	0,926049
	3,691915	0,844517	1,448906	0,716912
	4,45935	1,099549	0,85863	0,315536
	6,831443	0,614851	0,216185	1,141531
Mean	4,949	0,9985	0,899	0,7629
Std. Deviation	1,183	0,263	0,5047	0,306
Std. Error of Mean	0,5289	0,1176	0,2257	0,1369
**Shapiro-Wilk test**				
W	0,9199	0,8886	0,9509	0,9612
P value	0,5291	0,3503	0,7435	0,8162
Passed normality test (alpha=0.05)?	Yes	Yes	Yes	Yes
P value summary	ns	ns	ns	ns

	Control	Endurance	Interval	Strength
Time course - 6h	0,332478	0,448407	0,32383	0,272337
	2,906912	0,22329	0,338802	0,379644
	1,34486	0,13705	0,122896	0,301742
	0,850245	0,241752	0,450278	0,294802
	5,764596	0,239111	0,493953	0,56176
Mean	2,24	0,2579	0,346	0,3621
Std. Deviation	2,193	0,1148	0,1441	0,1187
Std. Error of Mean	0,9808	0,05134	0,06445	0,0531
**Shapiro-Wilk test**				
W	0,8767	0,8478	0,9244	0,8032
P value	0,2944	0,1877	0,559	0,086
Passed normality test (alpha=0.05)?	Yes	Yes	Yes	Yes
P value summary	ns	ns	ns	ns

	Control	Endurance	Interval	Strength
Time course - 12h	0,971464	0,4747	0,547758	0,762647
	1,026663	0,50335	0,504789	0,349892
	1,560177	0,468964	0,716195	0,217012
	0,450928	0,592308	0,829774	0,573393
	0,990767	0,878357	0,664881	0,311006
Mean	1	0,5835	0,6527	0,4428
Std. Deviation	0,3927	0,172	0,1308	0,2216
Std. Error of Mean	0,1756	0,07694	0,05849	0,09908
**Shapiro-Wilk test**				
W	0,9132	0,7626	0,9627	0,9242
P value	0,4872	0,0388	0,8263	0,5575
Passed normality test (alpha=0.05)?	Yes	No	Yes	Yes
P value summary	ns	*	ns	ns

	Control	Endurance	Interval	Strength
Time course - 18h	1,987098	1,909376	0,486707	1,803743
	1,512864	0,593307	0,91855	2,358331
	1,342884	0,704574	2,244544	1,669121
	1,330003	1,693645	2,207905	1,855548
	1,416448	0,410538	3,810463	1,373319
Mean	1,518	1,062	1,934	1,812
Std. Deviation	0,2722	0,6872	1,306	0,3582
Std. Error of Mean	0,1217	0,3073	0,5839	0,1602
**Shapiro-Wilk test**				
W	0,7701	0,8429	0,9381	0,9537
P value	0,0452	0,173	0,6528	0,7637
Passed normality test (alpha=0.05)?	No	Yes	Yes	Yes
P value summary	*	ns	ns	ns

Figure 3 - Gastrocnemius

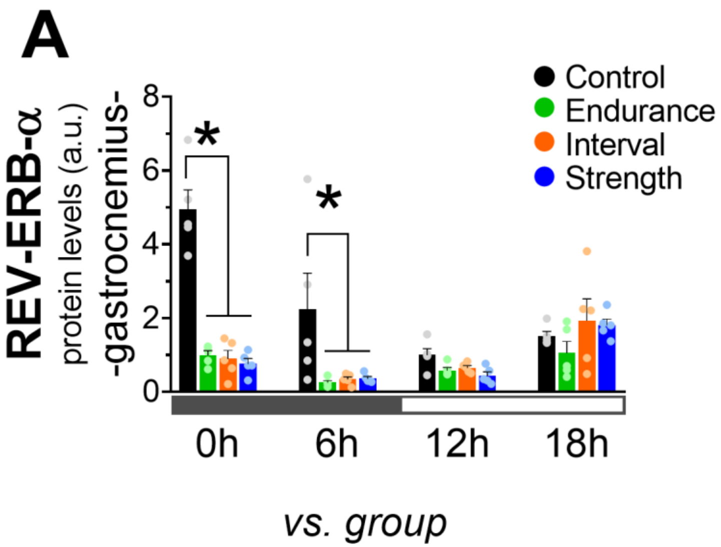

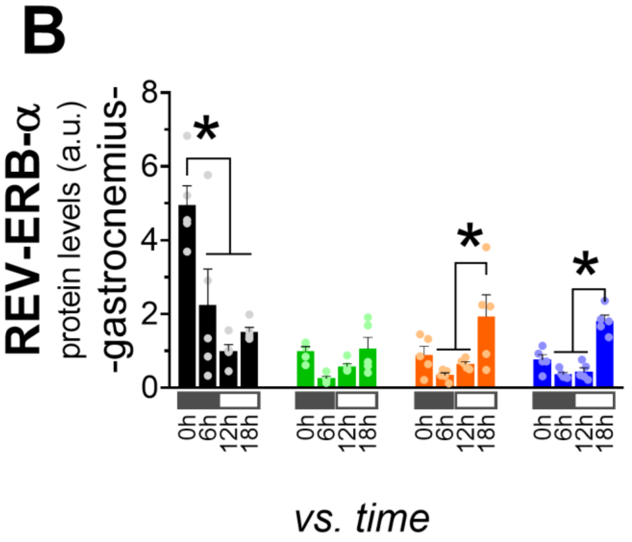

Two-way ANOVA	Ordinary
Alpha	0,05

Source of Variation	% of total variation	P value	P value summary	Significant?
Interaction	29,62	<0,0001	****	Yes
Row Factor	15,59	<0,0001	****	Yes
Column Factor	27,74	<0,0001	****	Yes

ANOVA table	SS (Type III)	DF	MS	F (DFn, DFd)	P value
Interaction	40,76	9	4,529	F (9, 64) = 7,789	P<0,0001
Row Factor	21,45	3	7,151	F (3, 64) = 12,30	P<0,0001
Column Factor	38,17	3	12,72	F (3, 64) = 21,88	P<0,0001
Residual	37,21	64	0,5815		

Figure 3 - Gastrocnemius

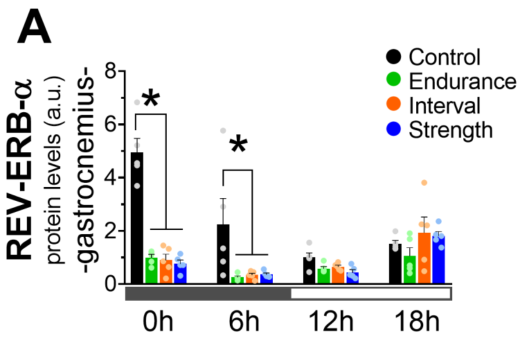

vs. group

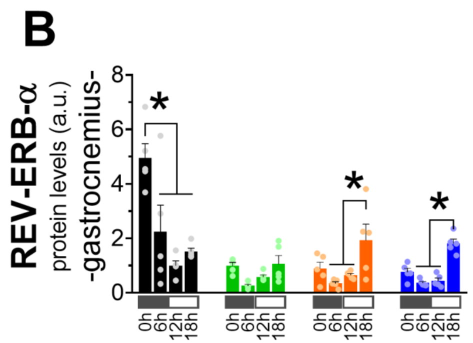

vs. time

Tukey's multiple comparisons test	Mean Diff.	95.00% CI of diff.	Significant?	Summary	Adjusted P Value
0h					
Control vs. Endurance	3,95	2,678 to 5,222	Yes	****	<0,0001
Control vs. Interval	4,05	2,777 to 5,322	Yes	****	<0,0001
Control vs. Strength	4,186	2,914 to 5,458	Yes	****	<0,0001
Endurance vs. Interval	0,09945	-1,173 to 1,372	No	ns	0,9969
Endurance vs. Strength	0,2355	-1,037 to 1,508	No	ns	0,9614
Interval vs. Strength	0,1361	-1,136 to 1,408	No	ns	0,9921
6h					
Control vs. Endurance	1,982	0,7097 to 3,254	Yes	***	0,0007
Control vs. Interval	1,894	0,6217 to 3,166	Yes	**	0,0012
Control vs. Strength	1,878	0,6056 to 3,150	Yes	**	0,0013
Endurance vs. Interval	-0,08803	-1,360 to 1,184	No	ns	0,9978
Endurance vs. Strength	-0,1041	-1,376 to 1,168	No	ns	0,9964
Interval vs. Strength	-0,01611	-1,288 to 1,256	No	ns	>0,9999
12h					
Control vs. Endurance	0,4165	-0,8557 to 1,689	No	ns	0,8235
Control vs. Interval	0,3473	-0,9248 to 1,619	No	ns	0,8887
Control vs. Strength	0,5572	-0,7149 to 1,829	No	ns	0,6569
Endurance vs. Interval	-0,06914	-1,341 to 1,203	No	ns	0,9989
Endurance vs. Strength	0,1407	-1,131 to 1,413	No	ns	0,9913
Interval vs. Strength	0,2099	-1,062 to 1,482	No	ns	0,9722
18h					
Control vs. Endurance	0,4556	-0,8166 to 1,728	No	ns	0,7809
Control vs. Interval	-0,4158	-1,688 to 0,8564	No	ns	0,8243
Control vs. Strength	-0,2942	-1,566 to 0,9780	No	ns	0,9286
Endurance vs. Interval	-0,8713	-2,143 to 0,4008	No	ns	0,2797
Endurance vs. Strength	-0,7497	-2,022 to 0,5224	No	ns	0,4117
Interval vs. Strength	0,1216	-1,151 to 1,394	No	ns	0,9943

Tukey's multiple comparisons test	Mean Diff.	95.00% CI of diff.	Significant?	Summary	Adjusted P Value
Control					
0h vs. 6h	2,709	1,437 to 3,981	Yes	****	<0,0001
0h vs. 12h	3,949	2,676 to 5,221	Yes	****	<0,0001
0h vs. 18h	3,431	2,159 to 4,703	Yes	****	<0,0001
6h vs. 12h	1,24	-0,03233 to 2,512	No	ns	0,0588
6h vs. 18h	0,722	-0,5502 to 1,994	No	ns	0,4453
12h vs. 18h	-0,5179	-1,790 to 0,7543	No	ns	0,7066
Endurance					
0h vs. 6h	0,7405	-0,5316 to 2,013	No	ns	0,4227
0h vs. 12h	0,4149	-0,8572 to 1,687	No	ns	0,8251
0h vs. 18h	-0,06384	-1,336 to 1,208	No	ns	0,9992
6h vs. 12h	-0,3256	-1,598 to 0,9465	No	ns	0,9061
6h vs. 18h	-0,8044	-2,077 to 0,4678	No	ns	0,349
12h vs. 18h	-0,4788	-1,751 to 0,7934	No	ns	0,7541
Interval					
0h vs. 6h	0,553	-0,7191 to 1,825	No	ns	0,6622
0h vs. 12h	0,2463	-1,026 to 1,518	No	ns	0,9562
0h vs. 18h	-1,035	-2,307 to 0,2375	No	ns	0,15
6h vs. 12h	-0,3067	-1,579 to 0,9654	No	ns	0,92
6h vs. 18h	-1,588	-2,860 to -0,3155	Yes	**	0,0086
12h vs. 18h	-1,281	-2,553 to - 0,008803	Yes	*	0,0478
Strength					
0h vs. 6h	0,4009	-0,8713 to 1,673	No	ns	0,8394
0h vs. 12h	0,3201	-0,9520 to 1,592	No	ns	0,9103
0h vs. 18h	-1,049	-2,321 to 0,2231	No	ns	0,1412
6h vs. 12h	-0,08073	-1,353 to 1,191	No	ns	0,9983
6h vs. 18h	-1,45	-2,722 to -0,1778	Yes	*	0,0193
12h vs. 18h	-1,369	-2,641 to -0,09707	Yes	*	0,0301

Figure 4 - Gastrocnemius

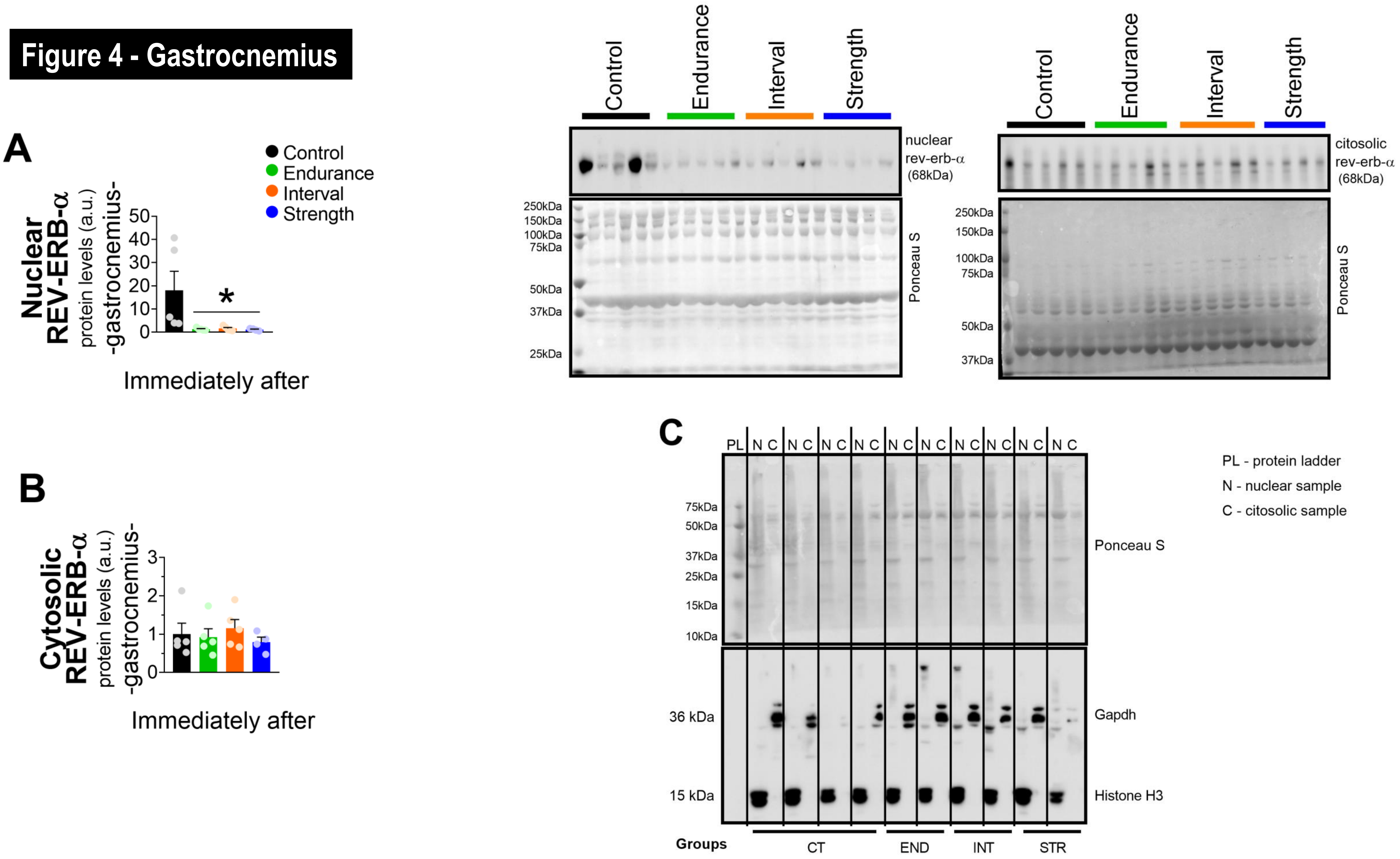

Figure 4 - Gastrocnemius

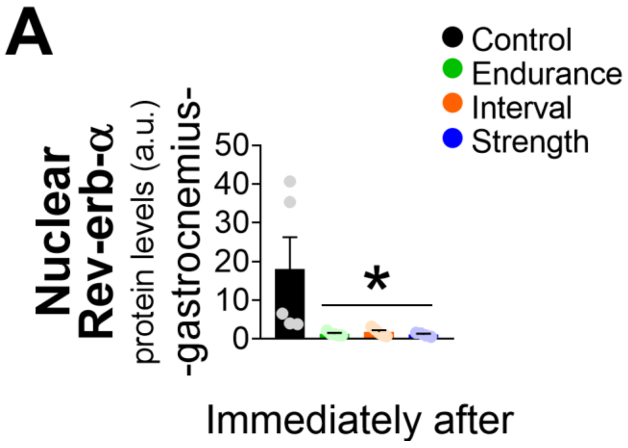

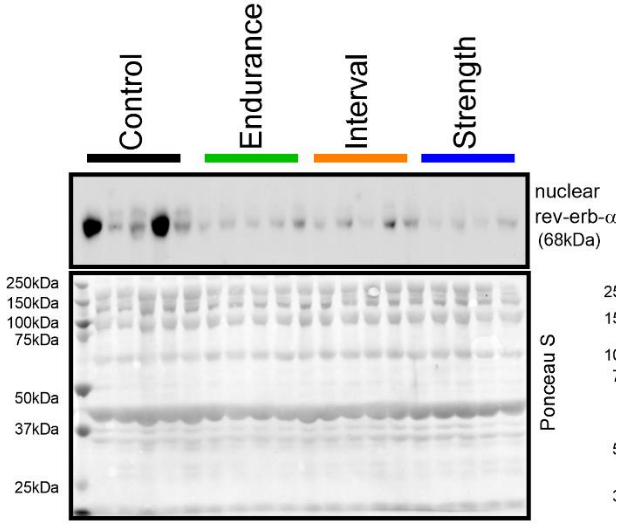

Ponceau quantification

	Control	Endurance	Interval	Strength
	37586	27112	33636	37254
	30316	33667	27928	41980
	40048	29401	29429	38171
	28543	23985	39133	31568
	33815	34273	43768	
Mean	34062	29688	34779	37243
Std. Deviation	4817	4362	6645	4301
Std. Error of Mean	2154	1951	2972	2151

ANOVA summary	
F	1.709
P value	0.2078
P value summary	ns
Significant diff. among means (P < 0.05)?	No
R square	0.2548

Figure 4 - Gastrocnemius

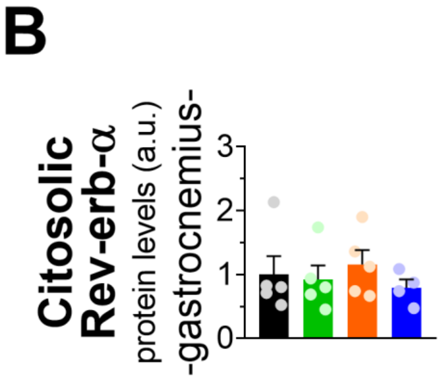

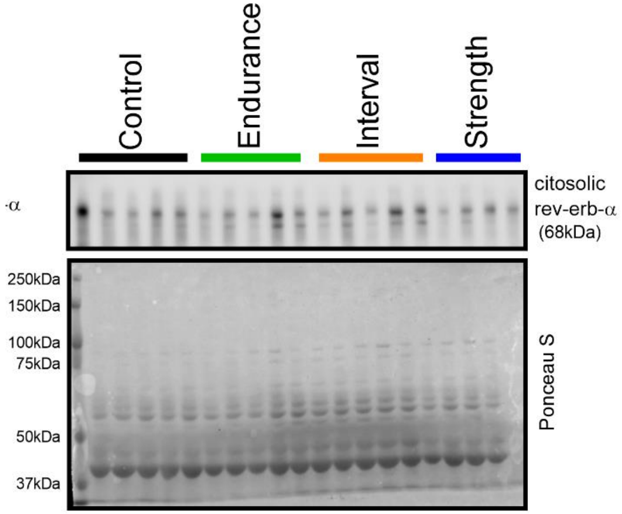

Ponceau quantification

	Control	Endurance	Interval	Strength
	37844	30857	28642	31610
	32248	28473	26586.5	36601
	33813	24036	31406	33699
	29325	36346	34559	23890
	40716	23226	36698	
Mean	34789	28588	31578	31450
Std. Deviation	4520	5358	4143	5440
Std. Error of Mean	2021	2396	1853	2720

ANOVA summary	
F	1.360
P value	0.2928
P value summary	ns
Significant diff. among means (P < 0.05)?	No
R square	0.2139

Figure 4 - Gastrocnemius

Original images for Western Blot

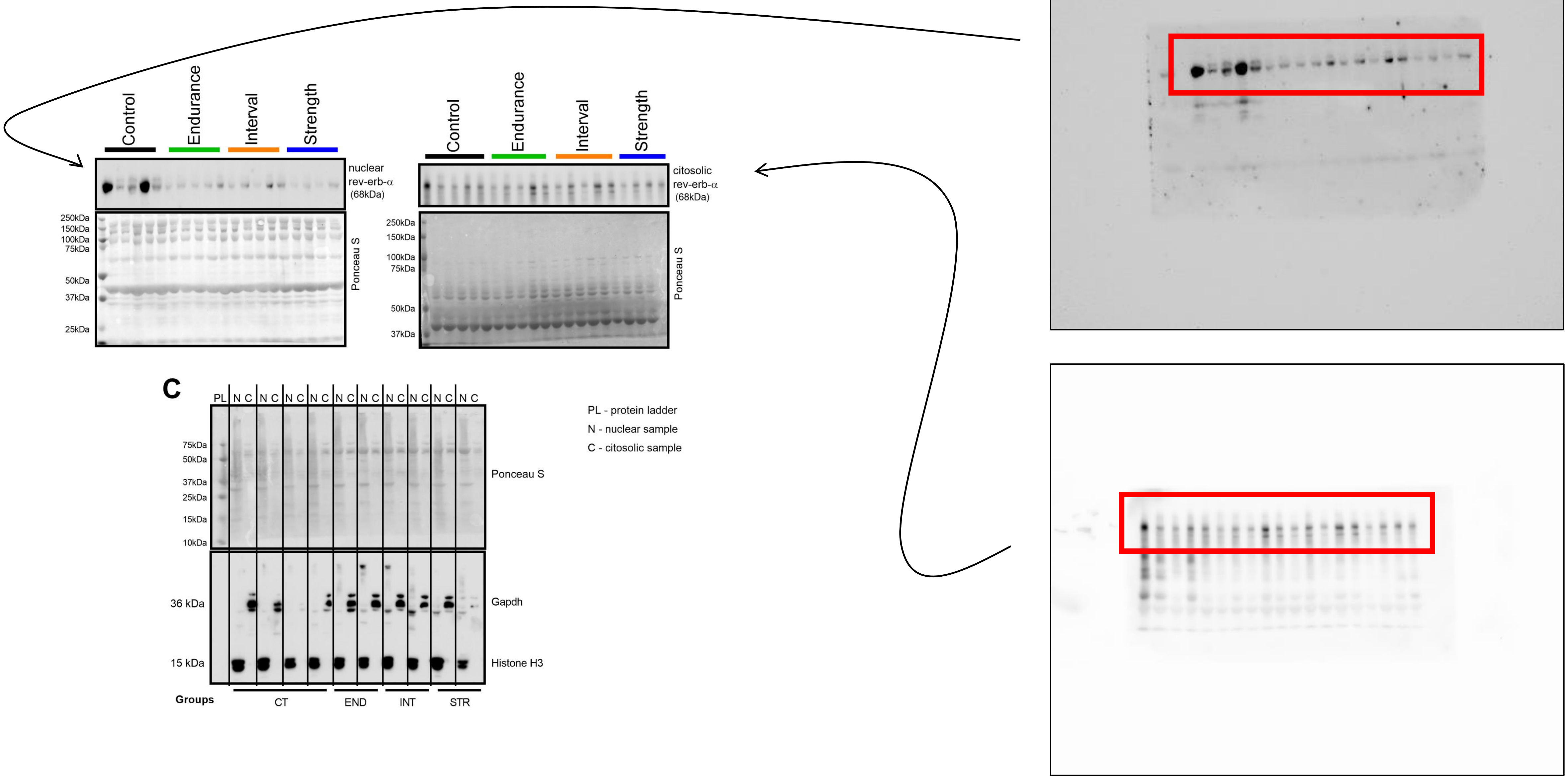

Figure 4 - Gastrocnemius

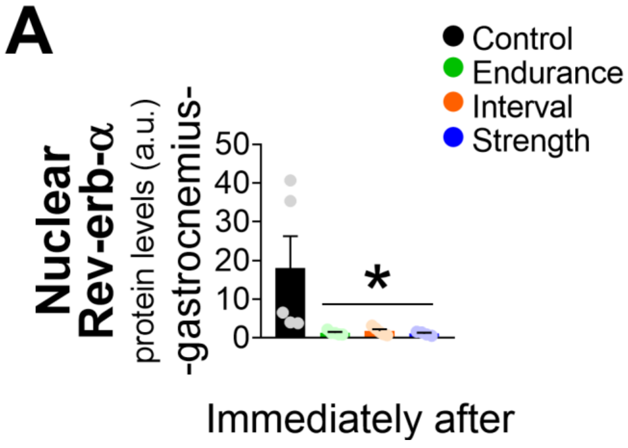

	Control	Endurance	Interval	Strength
Nuclear	35.378826	0.899118	0.9833709	0.8867829
	6.5432942	1.2704534	1.1591566	0.5734557
	4.0303656	0.9404313	0.6704416	1.590545
	40.745681	1.2977015	2.9989807	1.4337171
	3.8037307	2.189763	2.1410663	
Mean	18.1	1.319	1.591	1.121
Std. Deviation	18.35	0.5198	0.9602	0.4736
Std. Error of Mean	8.208	0.2324	0.4294	0.2368
Shapiro-Wilk test				
W	0.7657	0.8247	0.9051	0.9274
P value	0.0414	0.1269	0.4388	0.5792
Passed normality test (alpha=0.05)?	No	Yes	Yes	Yes
P value summary	*	ns	ns	ns
Kruskal-Wallis test				
P value	0.0129			
Exact or approximate P value?	Approximate			
P value summary	*			
Do the medians vary signif. (P < 0.05)?	Yes			
Number of groups	4			
Kruskal-Wallis statistic	10.79			

Dunn's multiple comparisons test	Mean rank diff.	Significant?	Summary	Adjusted P Value	A-?	Dunn's multiple comparisons test
Control vs. Endurance	9.2	Yes	*	0.0292	B	Control vs. Endurance
Control vs. Interval	8.8	Yes	*	0.0402	C	Control vs. Interval
Control vs. Strength	10.75	Yes	*	0.0132	D	Control vs. Strength

Figure 4 - Gastrocnemius

B

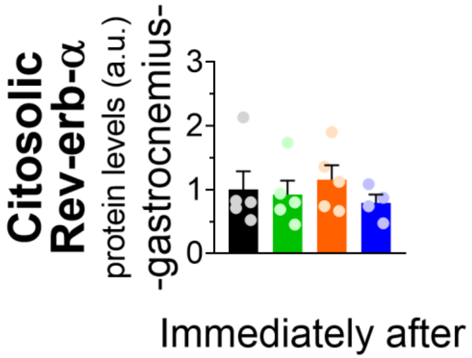

	Control	Endurance	Interval	Strength
Citosolic	2.133989	0.453292	0.742183	0.473943
	0.71126	0.800105	1.124686	0.874978
	0.523603	0.688458	0.665289	1.086372
	0.803837	1.73973	1.902415	0.742374
	0.827311	0.938011	1.361281	
Mean	1	0.9239	1.159	0.7944
Std. Deviation	0.6451	0.4894	0.5031	0.2563
Std. Error of Mean	0.2885	0.2189	0.225	0.1282
Shapiro-Wilk test				
W	0.7247	0.8744	0.9327	0.9953
P value	0.0171	0.2847	0.615	0.9827
Passed normality test (alpha=0.05)?	No	Yes	Yes	Yes
P value summary	*	ns	ns	ns
Kruskal-Wallis test				
P value	0.8201			
Exact or approximate P value?	Approximate			
P value summary	ns			
Do the medians vary signif. (P < 0.05)?	No			
Number of groups	4			
Kruskal-Wallis statistic	0.9221			

Figure 5 - Gastrocnemius

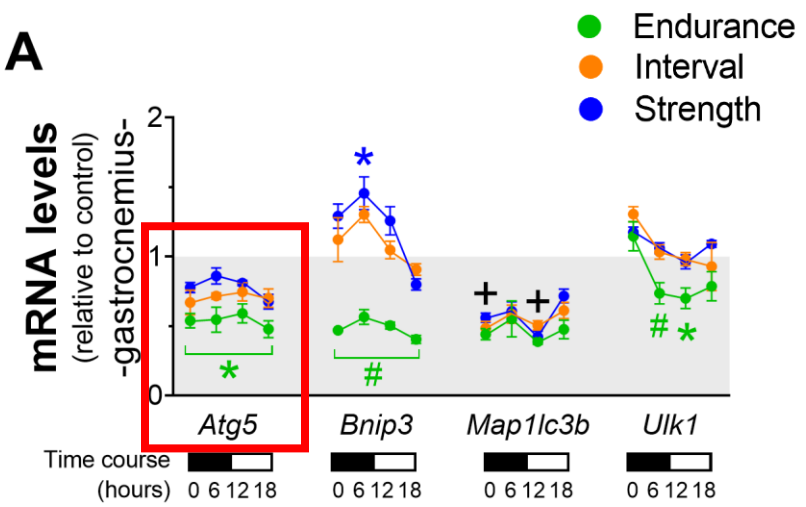

Atg5 - Gastrocnemius

	Control				Endurance				Interval				Strength			
	0h	6h	12h	18h	0h	6h	12h	18h	0h	6h	12h	18h	0h	6h	12h	18h
	1	0.507	0.579	0.673	0.397	0.475	0.458	0.227	0.243	0.416	0.469	0.36	0.413	0.438	0.511	0.362
	0.371	0.695	0.696	0.719	0.326	0.261	0.282	0.366	0.395	0.349	0.522	0.403	0.489	0.372	0.454	0.507
	0.558	0.633	0.914	0.352	0.268	0.263	0.39	0.348	0.477	0.404	0.528	0.579	0.372	0.518	0.489	0.384
	0.622	0.37	0.602	0.707	0.251	0.182	0.297	0.232	0.364	0.392	0.313	0.446	0.414	0.551	0.474	0.404
	0.206		0.225		0.247	0.326				0.414	0.421	0.359	0.456	0.494	0.515	
Mean	0.5514	0.5513	0.6032	0.6128	0.2978	0.3014	0.3568	0.2933	0.3698	0.395	0.4506	0.4294	0.4288	0.4746	0.4886	0.4143
Std. Deviation	0.2992	0.1439	0.2494	0.1749	0.06383	0.1097	0.08269	0.07401	0.09702	0.02742	0.08837	0.091	0.04489	0.07065	0.02554	0.06417
Std. Error of Mean	0.1338	0.07197	0.1115	0.08746	0.02854	0.04904	0.04135	0.037	0.04851	0.01226	0.03952	0.0407	0.02007	0.03159	0.01142	0.03208

Figure 5 - Gastrocnemius

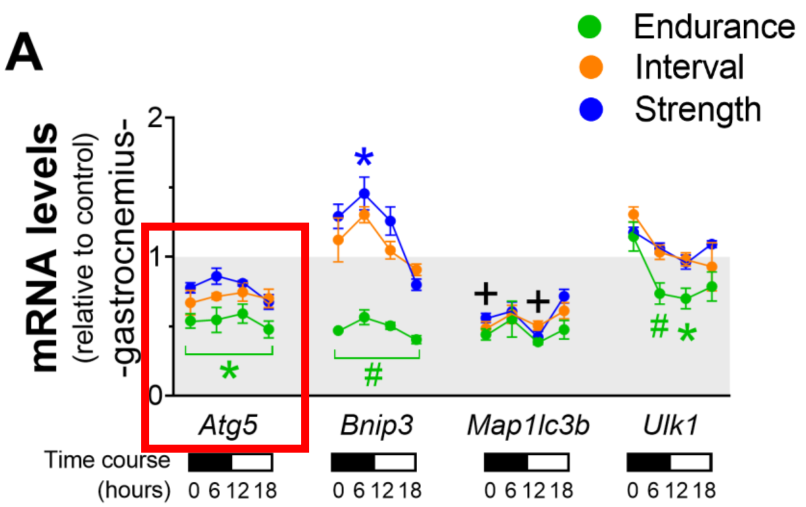

Two-way ANOVA	Ordinary
Alpha	0.05

Source of Variation	% of total variation	P value	P value summary	Significant?
Interaction	1.311	0.998	ns	No
Row Factor	2.273	0.5219	ns	No
Column Factor	37.88	<0.0001	****	Yes

Tukey's multiple comparisons test	Mean Diff.	95.00% CI of diff.	Significant?	Summary	Adjusted P Value
0h					
Control vs. Endurance	0.2536	0.03384 to 0.4734	Yes	*	0.0175
Control vs. Interval	0.1817	-0.05144 to 0.4147	No	ns	0.178
Control vs. Strength	0.1226	-0.09716 to 0.3424	No	ns	0.4586
Endurance vs. Interval	-0.07195	-0.3050 to 0.1611	No	ns	0.8464
Endurance vs. Strength	-0.131	-0.3508 to 0.08876	No	ns	0.3997
Interval vs. Strength	-0.05905	-0.2921 to 0.1740	No	ns	0.9079
6h					
Control vs. Endurance	0.2499	0.01676 to 0.4829	Yes	*	0.0311
Control vs. Interval	0.1563	-0.07684 to 0.3893	No	ns	0.2965
Control vs. Strength	0.07665	-0.1564 to 0.3097	No	ns	0.8204
Endurance vs. Interval	-0.0936	-0.3134 to 0.1262	No	ns	0.6747
Endurance vs. Strength	-0.1732	-0.3930 to 0.04656	No	ns	0.1702
Interval vs. Strength	-0.0796	-0.2994 to 0.1402	No	ns	0.7736
12h					
Control vs. Endurance	0.2465	0.01336 to 0.4795	Yes	*	0.0343
Control vs. Interval	0.1526	-0.06716 to 0.3724	No	ns	0.2669
Control vs. Strength	0.1146	-0.1052 to 0.3344	No	ns	0.5172
Endurance vs. Interval	-0.09385	-0.3269 to 0.1392	No	ns	0.712
Endurance vs. Strength	-0.1319	-0.3649 to 0.1012	No	ns	0.4463
Interval vs. Strength	-0.038	-0.2578 to 0.1818	No	ns	0.9679
18h					
Control vs. Endurance	0.3195	0.07381 to 0.5652	Yes	**	0.0058
Control vs. Interval	0.1834	-0.04974 to 0.4164	No	ns	0.1716
Control vs. Strength	0.1985	-0.04719 to 0.4442	No	ns	0.1537
Endurance vs. Interval	-0.1362	-0.3692 to 0.09694	No	ns	0.4178
Endurance vs. Strength	-0.121	-0.3667 to 0.1247	No	ns	0.565
Interval vs. Strength	0.01515	-0.2179 to 0.2482	No	ns	0.9982

Figure 5 - Gastrocnemius

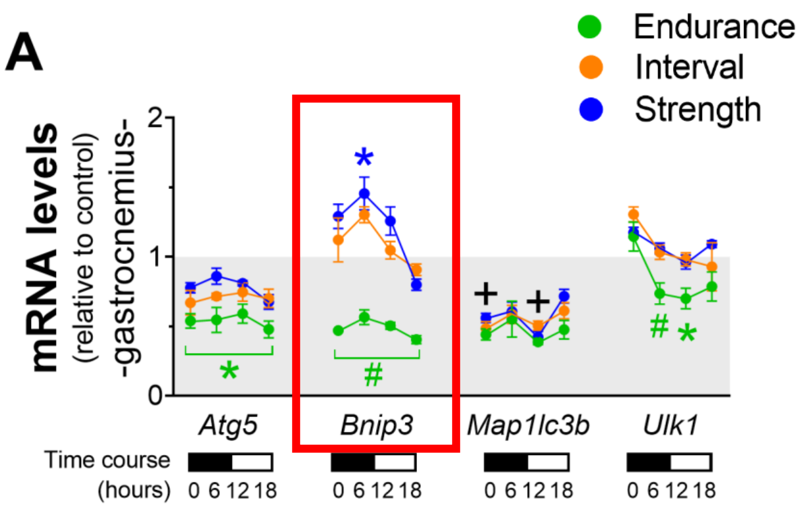

Bnip3 - Gastrocnemius

	Control				Endurance				Interval				Strength			
	**0h**	**6h**	**12h**	**18h**	**0h**	**6h**	**12h**	**18h**	**0h**	**6h**	**12h**	**18h**	**0h**	**6h**	**12h**	**18h**
	1	0.567	0.984	1.061	0.333	0.486	0.451	0.384	0.562	1.046	1.056	0.853	0.791	1.062	1.367	0.732
	0.629	0.885	1.146	1.234	0.45	0.343	0.521	0.512	0.805	0.81	1.005	1.016	1.129	0.994	0.948	0.87
	0.933	0.945	1.198	0.784	0.313	0.527	0.45	0.451	1.122	1.033	1.069	1.107	0.896	0.85	1.027	0.881
	0.88	0.591	0.748	1.198	0.324	0.322	0.414	0.386	1.004	0.973	0.852	0.979	1.094	1.149	1.371	0.936
	0.448		0.472		0.411	0.438				1.006	0.785	0.896	1.113	1.383	1.011	
Mean	0.778	0.747	0.9096	1.069	0.3662	0.4232	0.459	0.4333	0.8733	0.9736	0.9534	0.9702	1.005	1.088	1.145	0.8548
Std. Deviation	0.2317	0.1958	0.3009	0.2043	0.06071	0.0889	0.04477	0.06103	0.2453	0.09564	0.1277	0.1002	0.1522	0.1981	0.2068	0.08678
Std. Error of Mean	0.1036	0.09789	0.1346	0.1021	0.02715	0.03976	0.02239	0.03052	0.1226	0.04277	0.05709	0.04479	0.06806	0.08857	0.09248	0.04339

Figure 5 - Gastrocnemius

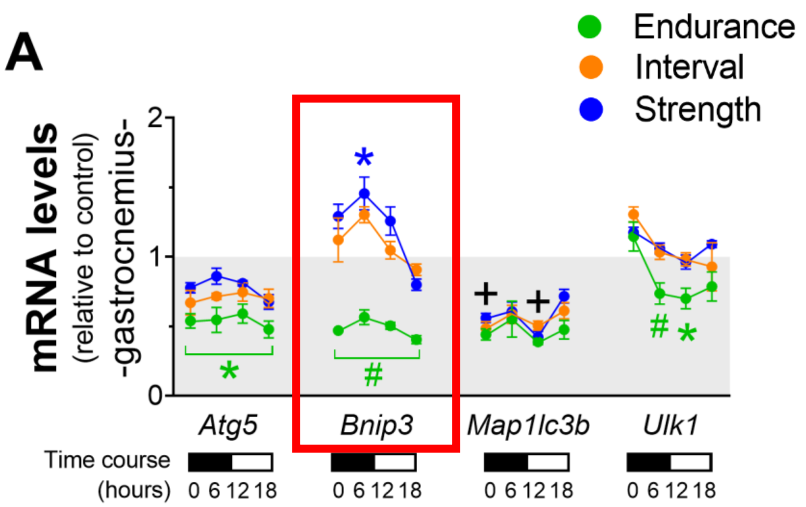

Tukey's multiple comparisons test	Mean Diff.	95.00% CI of diff.	Significant?	Summary	Adjusted P Value
0h					
Control vs. Endurance	0.4118	0.1300 to 0.6936	Yes	**	0.0016
Control vs. Interval	-0.09525	-0.3942 to 0.2037	No	ns	0.8338
Control vs. Strength	-0.2266	-0.5084 to 0.05525	No	ns	0.1568
Endurance vs. Interval	-0.5071	-0.8060 to -0.2081	Yes	***	0.0002
Endurance vs. Strength	-0.6384	-0.9202 to -0.3566	Yes	****	<0.0001
Interval vs. Strength	-0.1314	-0.4303 to 0.1676	No	ns	0.6528
6h					
Control vs. Endurance	0.3238	0.02486 to 0.6227	Yes	*	0.0288
Control vs. Interval	-0.2266	-0.5255 to 0.07234	No	ns	0.198
Control vs. Strength	-0.3406	-0.6395 to -0.04166	Yes	*	0.0195
Endurance vs. Interval	-0.5504	-0.8322 to -0.2686	Yes	****	<0.0001
Endurance vs. Strength	-0.6644	-0.9462 to -0.3826	Yes	****	<0.0001
Interval vs. Strength	-0.114	-0.3958 to 0.1678	No	ns	0.7091
12h					
Control vs. Endurance	0.4506	0.1517 to 0.7495	Yes	**	0.0011
Control vs. Interval	-0.0438	-0.3256 to 0.2380	No	ns	0.9763
Control vs. Strength	-0.2352	-0.5170 to 0.04665	No	ns	0.1333
Endurance vs. Interval	-0.4944	-0.7933 to -0.1955	Yes	***	0.0003
Endurance vs. Strength	-0.6858	-0.9847 to -0.3869	Yes	****	<0.0001
Interval vs. Strength	-0.1914	-0.4732 to 0.09045	No	ns	0.2855
18h					
Control vs. Endurance	0.636	0.3209 to 0.9511	Yes	****	<0.0001
Control vs. Interval	0.09905	-0.1999 to 0.3980	No	ns	0.817
Control vs. Strength	0.2145	-0.1006 to 0.5296	No	ns	0.2835
Endurance vs. Interval	-0.537	-0.8359 to -0.2380	Yes	****	<0.0001
Endurance vs. Strength	-0.4215	-0.7366 to -0.1064	Yes	**	0.0043
Interval vs. Strength	0.1155	-0.1835 to 0.4144	No	ns	0.7376

Two-way ANOVA	Ordinary
Alpha	0.05

Source of Variation	% of total variation	P value	P value summary	Significant?
Interaction	6.427	0.1419	ns	No
Row Factor	1.949	0.2403	ns	No
Column Factor	62.81	<0.0001	****	Yes

Figure 5 - Gastrocnemius

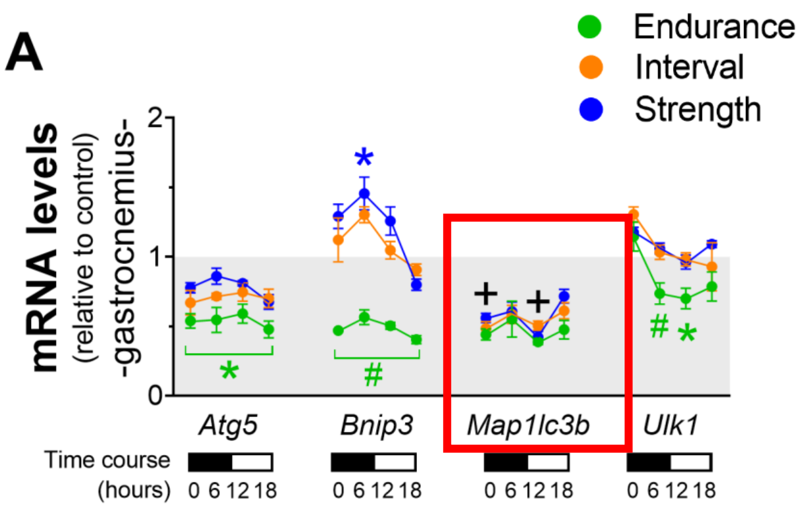

Map1lc3b - Gastrocnemius

	Control				Endurance				Interval				Strength			
	**0h**	**6h**	**12h**	**18h**	**0h**	**6h**	**12h**	**18h**	**0h**	**6h**	**12h**	**18h**	**0h**	**6h**	**12h**	**18h**
	1	0.463	0.533	0.445	0.234	0.348	0.304	0.17	0.251	0.418	0.343	0.279	0.342	0.282	0.345	0.279
	0.529	0.806	0.654	0.515	0.195	0.215	0.241	0.231	0.262	0.402	0.443	0.265	0.38	0.296	0.279	0.336
	0.752	0.61	1.607	0.478	0.315	0.628	0.296	0.316	0.378	0.451	0.391	0.383	0.287	0.357	0.382	0.376
	0.584	0.6	0.658	0.474	0.322	0.166	0.276	0.194	0.272	0.225	0.31	0.321	0.296	0.45	0.3	0.38
	0.335		0.144		0.26	0.353				0.321	0.332	0.216	0.378	0.497	0.216	
Mean	0.64	0.6198	0.7192	0.478	0.2652	0.342	0.2793	0.2278	0.2908	0.3634	0.3638	0.2928	0.3366	0.3764	0.3044	0.3428
Std. Deviation	0.2503	0.1411	0.5389	0.02872	0.05393	0.1796	0.02809	0.06396	0.0588	0.09096	0.05327	0.06283	0.04397	0.09449	0.06354	0.04691
Std. Error of Mean	0.1119	0.07056	0.241	0.01436	0.02412	0.08033	0.01404	0.03198	0.0294	0.04068	0.02382	0.0281	0.01967	0.04226	0.02842	0.02346

Figure 5 - Gastrocnemius

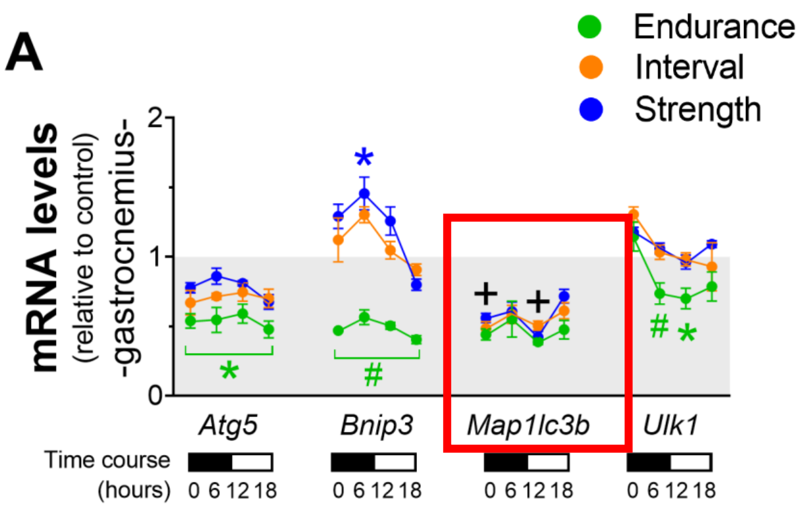

Two-way ANOVA	Ordinary
Alpha	0.05

Source of Variation	% of total variation	P value	P value summary	Significant?
Interaction	3.398	0.9278	ns	No
Row Factor	2.698	0.4164	ns	No
Column Factor	37.86	<0.0001	****	Yes

Tukey's multiple comparisons test	Mean Diff.	95.00% CI of diff.	Significant?	Summary	Adjusted P Value
0h					
Control vs. Endurance	0.3748	0.08283 to 0.6668	Yes	**	0.0066
Control vs. Interval	0.3493	0.03957 to 0.6589	Yes	*	0.0211
Control vs. Strength	0.3034	0.01143 to 0.5954	Yes	*	0.0387
Endurance vs. Interval	-0.02555	-0.3352 to 0.2841	No	ns	0.9963
Endurance vs. Strength	-0.0714	-0.3634 to 0.2206	No	ns	0.9162
Interval vs. Strength	-0.04585	-0.3555 to 0.2638	No	ns	0.9794
6h					
Control vs. Endurance	0.2778	-0.03193 to 0.5874	No	ns	0.0939
Control vs. Interval	0.2564	-0.05333 to 0.5660	No	ns	0.1383
Control vs. Strength	0.2434	-0.06633 to 0.5530	No	ns	0.1723
Endurance vs. Interval	-0.0214	-0.3134 to 0.2706	No	ns	0.9974
Endurance vs. Strength	-0.0344	-0.3264 to 0.2576	No	ns	0.9894
Interval vs. Strength	-0.013	-0.3050 to 0.2790	No	ns	0.9994
12h					
Control vs. Endurance	0.44	0.1303 to 0.7496	Yes	**	0.0022
Control vs. Interval	0.3554	0.06343 to 0.6474	Yes	*	0.011
Control vs. Strength	0.4148	0.1228 to 0.7068	Yes	**	0.0022
Endurance vs. Interval	-0.08455	-0.3942 to 0.2251	No	ns	0.8879
Endurance vs. Strength	-0.02515	-0.3348 to 0.2845	No	ns	0.9965
Interval vs. Strength	0.0594	-0.2326 to 0.3514	No	ns	0.9493
18h					
Control vs. Endurance	0.2503	-0.07618 to 0.5767	No	ns	0.1898
Control vs. Interval	0.1852	-0.1245 to 0.4949	No	ns	0.3968
Control vs. Strength	0.1353	-0.1912 to 0.4617	No	ns	0.6934
Endurance vs. Interval	-0.06505	-0.3747 to 0.2446	No	ns	0.9447
Endurance vs. Strength	-0.115	-0.4414 to 0.2114	No	ns	0.7879
Interval vs. Strength	-0.04995	-0.3596 to 0.2597	No	ns	0.9737

Figure 5 - Gastrocnemius

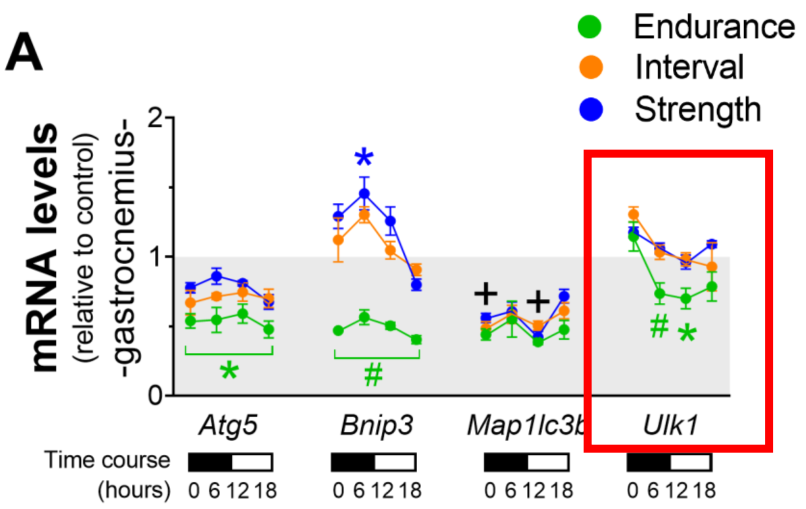

Ulk1 - Gastrocnemius

	Control				Endurance				Interval				Strength			
	0h	6h	12h	18h	0h	6h	12h	18h	0h	6h	12h	18h	0h	6h	12h	18h
	1	0.686	1.086	0.894	0.856	0.524	0.743	0.743	1.136	0.775	0.853	0.973	0.828	1.006	0.859	0.958
	0.472	0.95	0.854	0.636	0.967	0.564	0.587	0.562	0.957	1.017	0.878	0.95	0.973	0.964	0.915	0.916
	0.9	0.925	0.952	0.92	1.068	0.558	0.783	0.465	0.986	0.957	0.911	0.953	0.953	0.847	0.917	0.862
	0.951	0.867	0.781	0.878	0.958	0.689	0.487	0.851	0.969	0.823	0.839	0.774	0.853	0.898	1.031	0.896
	0.552		0.966		0.593	0.523				0.857	1.078	0.216	0.947	0.83	0.743	
Mean	0.775	0.857	0.9278	0.832	0.8884	0.5716	0.65	0.6553	1.012	0.8858	0.9118	0.7732	0.9108	0.909	0.893	0.908
Std. Deviation	0.2443	0.1192	0.1163	0.1318	0.1814	0.06829	0.1377	0.1741	0.08352	0.09916	0.09685	0.3217	0.06549	0.07523	0.1046	0.0401
Std. Error of Mean	0.1093	0.05959	0.05199	0.0659	0.08111	0.03054	0.06885	0.08704	0.04176	0.04435	0.04331	0.1439	0.02929	0.03365	0.04678	0.02005

Figure 5 - Gastrocnemius

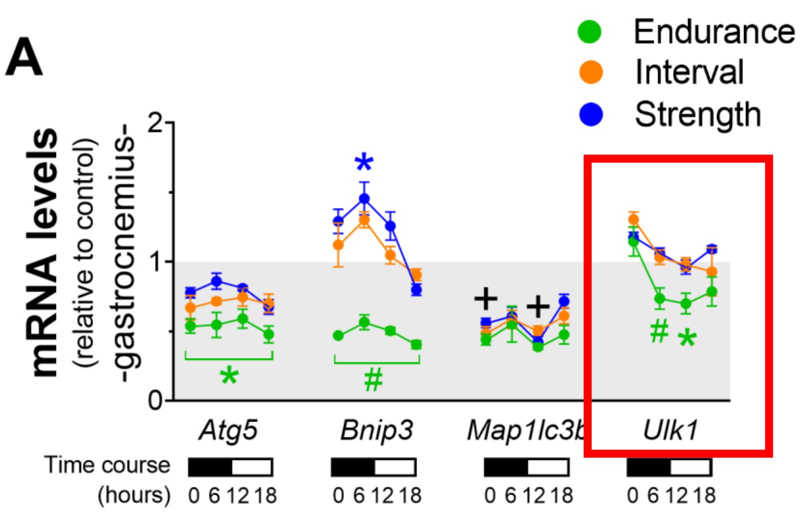

Two-way ANOVA	Ordinary
Alpha	0.05

Source of Variation	% of total variation	P value	P value summary	Significant?
Interaction	15.22	0.1046	ns	No
Row Factor	5.331	0.1556	ns	No
Column Factor	23.57	0.0002	***	Yes

Tukey's multiple comparisons test	Mean Diff.	95.00% CI of diff.	Significant?	Summary	Adjusted P Value
0h					
Control vs. Endurance	-0.1134	-0.3625 to 0.1357	No	ns	0.6267
Control vs. Interval	-0.237	-0.5012 to 0.02719	No	ns	0.0938
Control vs. Strength	-0.1358	-0.3849 to 0.1133	No	ns	0.4788
Endurance vs. Interval	-0.1236	-0.3878 to 0.1406	No	ns	0.6059
Endurance vs. Strength	-0.0224	-0.2715 to 0.2267	No	ns	0.9952
Interval vs. Strength	0.1012	-0.1630 to 0.3654	No	ns	0.7424
6h					
Control vs. Endurance	0.2854	0.02121 to 0.5496	Yes	*	0.0294
Control vs. Interval	-0.0288	-0.2930 to 0.2354	No	ns	0.9916
Control vs. Strength	-0.052	-0.3162 to 0.2122	No	ns	0.9538
Endurance vs. Interval	-0.3142	-0.5633 to -0.06512	Yes	**	0.0079
Endurance vs. Strength	-0.3374	-0.5865 to -0.08832	Yes	**	0.0038
Interval vs. Strength	-0.0232	-0.2723 to 0.2259	No	ns	0.9947
12h					
Control vs. Endurance	0.2778	0.01361 to 0.5420	Yes	*	0.0357
Control vs. Interval	0.016	-0.2331 to 0.2651	No	ns	0.9982
Control vs. Strength	0.0348	-0.2143 to 0.2839	No	ns	0.9826
Endurance vs. Interval	-0.2618	-0.5260 to 0.002387	No	ns	0.053
Endurance vs. Strength	-0.243	-0.5072 to 0.02119	No	ns	0.0821
Interval vs. Strength	0.0188	-0.2303 to 0.2679	No	ns	0.9972
18h					
Control vs. Endurance	0.1768	-0.1017 to 0.4552	No	ns	0.344
Control vs. Interval	0.0588	-0.2054 to 0.3230	No	ns	0.9351
Control vs. Strength	-0.076	-0.3545 to 0.2025	No	ns	0.888
Endurance vs. Interval	-0.118	-0.3821 to 0.1462	No	ns	0.6412
Endurance vs. Strength	-0.2528	-0.5312 to 0.02573	No	ns	0.0882
Interval vs. Strength	-0.1348	-0.3990 to 0.1294	No	ns	0.5356

Figure 5 - Gastrocnemius

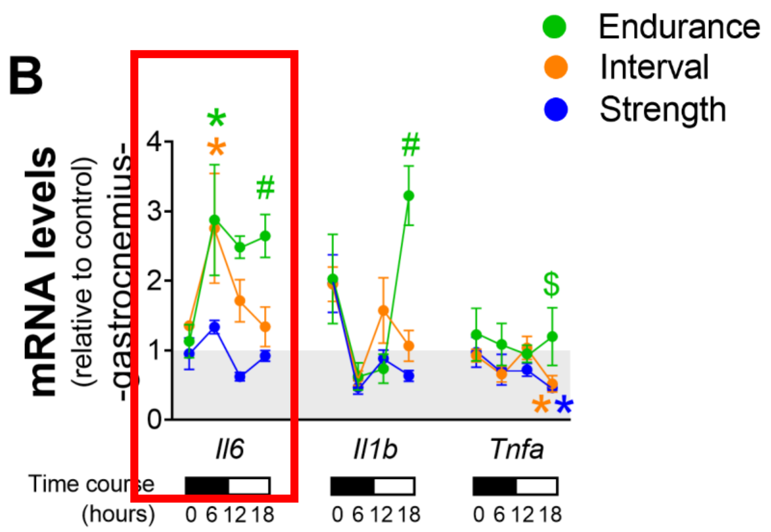

Il6 - Gastrocnemius

	Control				Endurance				Interval				Strength			
	**0h**	**6h**	**12h**	**18h**	**0h**	**6h**	**12h**	**18h**	**0h**	**6h**	**12h**	**18h**	**0h**	**6h**	**12h**	**18h**
	1	0.468	0.554	1.29	0.799	1.197	1.701	3.199	1.535	1.512	0.774	1.177	0.86	0.638	0.377	1.016
	1.288	0.689	0.545	0.862	1.759	1.138	1.597	1.803	1.442	3.393	1.481	0.97	2.137	0.781	0.255	0.716
	1.45	0.73	0.695	0.828	1.728	5.48	1.364	2.96	1.574	1.339	0.874	2.26	0.786	0.865	0.398	1.054
	1.138	0.536	0.661		0.495	1.389		2.556	1.705	1.856	1.574	0.628	0.962	0.771	0.454	0.877
	0.894		0.67		1.75	1.324				0.848	0.656	1.621	0.753	0.991	0.461	
Mean	1.154	0.6058	0.625	0.9933	1.306	2.106	1.554	2.63	1.564	1.79	1.072	1.331	1.1	0.8092	0.389	0.9158
Std. Deviation	0.222	0.1241	0.07011	0.2575	0.6114	1.889	0.1726	0.6116	0.1091	0.9672	0.4244	0.6315	0.5855	0.1301	0.08305	0.1534
Std. Error of Mean	0.09928	0.06205	0.03135	0.1487	0.2734	0.8448	0.09963	0.3058	0.05455	0.4326	0.1898	0.2824	0.2618	0.05819	0.03714	0.07668

Figure 5 - Gastrocnemius

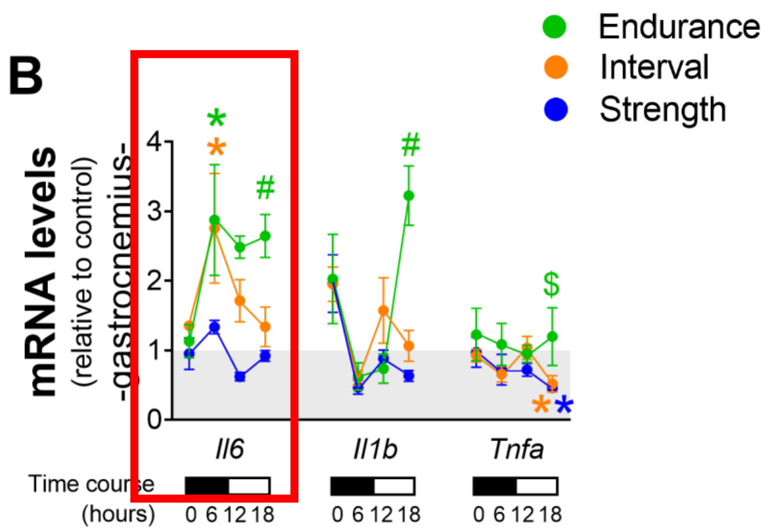

Two-way ANOVA	Ordinary
Alpha	0.05

Source of Variation	% of total variation	P value	P value summary	Significant?
Interaction	11.14	0.2465	ns	No
Row Factor	5.965	0.1073	ns	No
Column Factor	29.25	<0.0001	****	Yes

Tukey's multiple comparisons test	Mean Diff.	95.00% CI of diff.	Significant?	Summary	Adjusted P Value	
0h						
Control vs. Endurance	-0.1522	-1.271 to 0.9662	No	ns	0.9838	
Control vs. Interval	-0.41	-1.596 to 0.7762	No	ns	0.7968	
Control vs. Strength	0.0544	-1.064 to 1.173	No	ns	0.9992	
Endurance vs. Interval	-0.2578	-1.444 to 0.9284	No	ns	0.939	
Endurance vs. Strength	0.2066	-0.9118 to 1.325	No	ns	0.9612	
Interval vs. Strength	0.4644	-0.7218 to 1.651	No	ns	0.7288	
6h						
Control vs. Endurance	-1.5	-2.686 to -0.3136	Yes	**	0.0077	
Control vs. Interval	-1.184	-2.370 to 0.002382	No	ns	0.0506	
Control vs. Strength	-0.2035	-1.390 to 0.9828	No	ns	0.9686	
Endurance vs. Interval	0.316	-0.8024 to 1.434	No	ns	0.8771	
Endurance vs. Strength	1.296	0.1780 to 2.415	Yes	*	0.0169	*
Interval vs. Strength	0.9804	-0.1380 to 2.099	No	ns	0.1054	
12h						
Control vs. Endurance	-0.929	-2.220 to 0.3624	No	ns	0.2377	
Control vs. Interval	-0.4468	-1.565 to 0.6716	No	ns	0.7163	
Control vs. Strength	0.236	-0.8824 to 1.354	No	ns	0.9438	
Endurance vs. Interval	0.4822	-0.8092 to 1.774	No	ns	0.7564	
Endurance vs. Strength	1.165	-0.1264 to 2.456	No	ns	0.091	
Interval vs. Strength	0.6828	-0.4356 to 1.801	No	ns	0.3778	
18h						
Control vs. Endurance	-1.636	-2.987 to -0.2856	Yes	*	0.0115	
Control vs. Interval	-0.3379	-1.629 to 0.9535	No	ns	0.8994	
Control vs. Strength	0.07758	-1.273 to 1.428	No	ns	0.9987	
Endurance vs. Interval	1.298	0.1121 to 2.485	Yes	*	0.0267	
Endurance vs. Strength	1.714	0.4634 to 2.964	Yes	**	0.0034	
Interval vs. Strength	0.4155	-0.7708 to 1.602	No	ns	0.7903	

* Data not plotted on the graph (the main aim was to highlight the differences in relation to control)

Figure 5 - Gastrocnemius

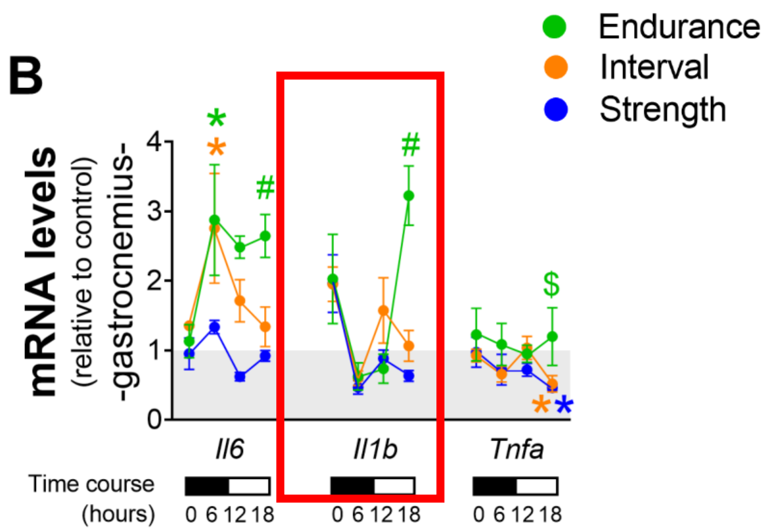

`

Il1beta - Gastrocnemius

	Control				Endurance				Interval				Strength			
	**0h**	**6h**	**12h**	**18h**	**0h**	**6h**	**12h**	**18h**	**0h**	**6h**	**12h**	**18h**	**0h**	**6h**	**12h**	**18h**
	1	0.573	1.513	1.905	1.641	0.832	0.773	7.354	1.933	0.741	1.184	2.309	2.284	0.318	2.545	1.404
		2.445	4.073	2.606	1.362	1.753	2.172	8.409	3.621	1.532	4.583	1.223		0.701	2.808	1.77
	0.489	1.847	2.149	3.088	4.499		2.345	5.252	1.778	0.946	6.62	4.1	1.348	1.178	1.118	0.942
	1.406	2.426	1.623	1.085	2.106	1.699			2.11	0.685	5.38	2.094	3.032	1.309	1.896	1.387
	1.166		2.611			0.273				1.551	1.085	1.838	2.57	0.772	2.194	
Mean	1.015	1.823	2.394	2.171	2.402	1.139	1.763	7.005	2.361	1.091	3.77	2.313	2.309	0.8556	2.112	1.376
Std. Deviation	0.3884	0.8782	1.036	0.8718	1.431	0.7153	0.862	1.607	0.8512	0.4226	2.514	1.079	0.7106	0.3967	0.6545	0.3389
Std. Error of Mean	0.1942	0.4391	0.4635	0.4359	0.7156	0.3576	0.4977	0.9279	0.4256	0.189	1.124	0.4824	0.3553	0.1774	0.2927	0.1694

Figure 5 - Gastrocnemius

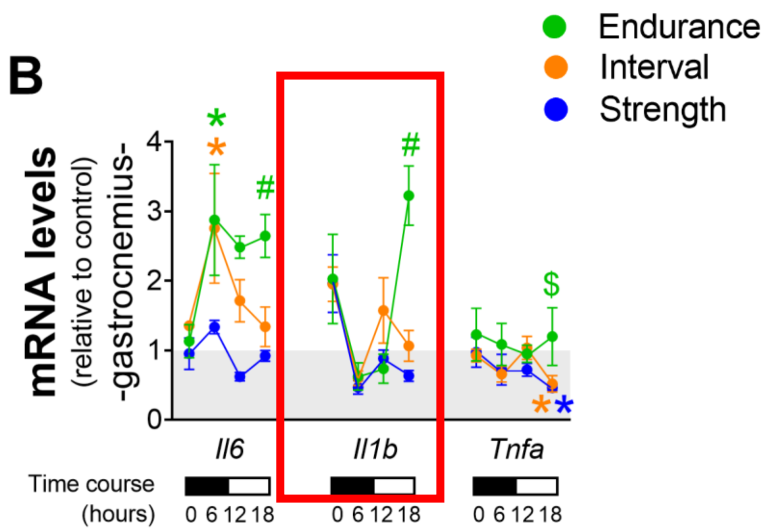

Two-way ANOVA	Ordinary
Alpha	0.05

Source of Variation	% of total variation	P value	P value summary	Significant?
Interaction	38.22	<0.0001	****	Yes
Row Factor	20.33	<0.0001	****	Yes
Column Factor	10.68	0.0031	**	Yes

Tukey's multiple comparisons test	Mean Diff.	95.00% CI of diff.	Significant?	Summary	Adjusted P Value
0h					
Control vs. Endurance	-1.387	-3.414 to 0.6408	No	ns	0.2779
Control vs. Interval	-1.345	-3.373 to 0.6823	No	ns	0.3036
Control vs. Strength	-1.293	-3.321 to 0.7343	No	ns	0.3377
Endurance vs. Interval	0.04150	-1.986 to 2.069	No	ns	>0.9999
Endurance vs. Strength	0.09350	-1.934 to 2.121	No	ns	0.9993
Interval vs. Strength	0.05200	-1.976 to 2.080	No	ns	0.9999
6h					
Control vs. Endurance	0.6835	-1.344 to 2.711	No	ns	0.8076
Control vs. Interval	0.7317	-1.192 to 2.655	No	ns	0.7445
Control vs. Strength	0.9672	-0.9564 to 2.891	No	ns	0.5456
Endurance vs. Interval	0.04825	-1.875 to 1.972	No	ns	0.9999
Endurance vs. Strength	0.2837	-1.640 to 2.207	No	ns	0.9794
Interval vs. Strength	0.2354	-1.578 to 2.049	No	ns	0.9858
12h					
Control vs. Endurance	0.6305	-1.464 to 2.725	No	ns	0.8545
Control vs. Interval	-1.377	-3.190 to 0.4369	No	ns	0.1958
Control vs. Strength	0.2816	-1.532 to 2.095	No	ns	0.9761
Endurance vs. Interval	-2.007	-4.101 to 0.08698	No	ns	0.0648
Endurance vs. Strength	-0.3489	-2.443 to 1.745	No	ns	0.9708
Interval vs. Strength	1.658	-0.1553 to 3.472	No	ns	0.0845
18h					
Control vs. Endurance	-4.834	-7.024 to -2.644	Yes	****	<0.0001
Control vs. Interval	-0.1418	-2.065 to 1.782	No	ns	0.9973
Control vs. Strength	0.7952	-1.232 to 2.823	No	ns	0.7263
Endurance vs. Interval	4.692	2.598 to 6.786	Yes	****	<0.0001
Endurance vs. Strength	5.629	3.439 to 7.819	Yes	****	<0.0001
Interval vs. Strength	0.9370	-0.9865 to 2.861	No	ns	0.5715

Figure 5 - Gastrocnemius

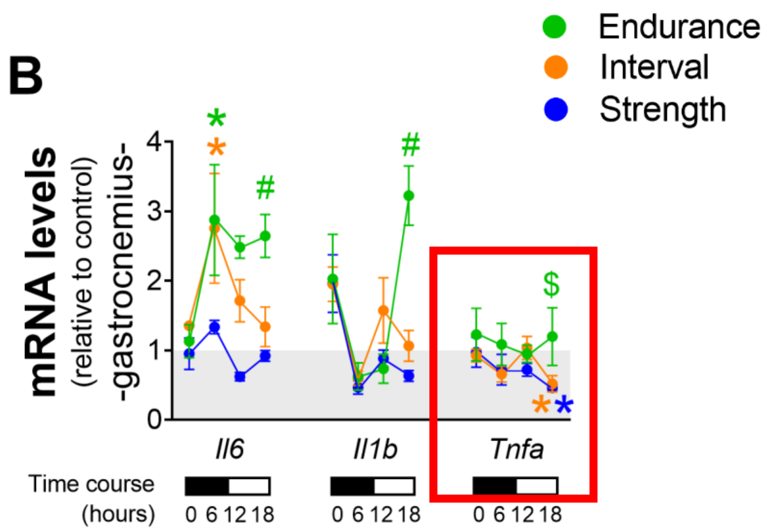

`

Tnfa - Gastrocnemius

	Control				Endurance				Interval				Strength			
	0h	6h	12h	18h	0h	6h	12h	18h	0h	6h	12h	18h	0h	6h	12h	18h
	1	1.286	0.881	2.309	1.002	0.961	1.206	1.782	0.835	0.554	1.101	0.839	1.244	0.159	0.533	1.367
	0.71	0.895	1.219	2.61		0.646	1.13	1.192	0.812	1.045	1.599	0.587	1.002	1.326	0.689	0.901
	0.888	0.777	1.601	3.92	1.718	2.233	0.772	5.838	0.735	0.478	1.075	2.3	0.344	1.101	0.955	1.15
	0.637	0.991	1.029	1.131	0.273	0.605	1.507	3.144	0.523	0.438	1.821	1.474	0.44	0.577	1.091	1.205
	0.685		1.334		0.853	0.916				0.738	0.688	1.262	0.787	0.407	1.102	
Mean	0.784	0.9873	1.213	2.493	0.9615	1.072	1.154	2.989	0.7263	0.6506	1.257	1.292	0.7634	0.714	0.874	1.156
Std. Deviation	0.1536	0.2175	0.2781	1.146	0.5943	0.6679	0.3021	2.068	0.1421	0.2488	0.4517	0.6619	0.3771	0.4861	0.2531	0.1932
Std. Error of Mean	0.06869	0.1088	0.1244	0.5729	0.2972	0.2987	0.1511	1.034	0.07104	0.1112	0.202	0.296	0.1687	0.2174	0.1132	0.0966

Figure 5 - Gastrocnemius

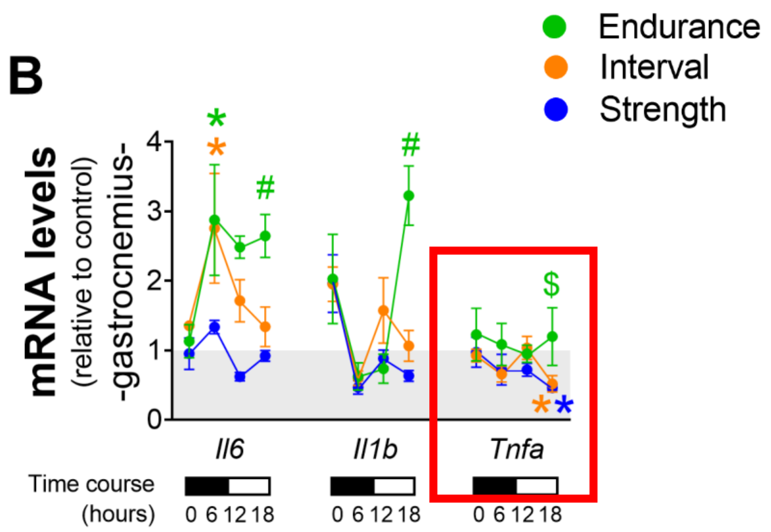

Two-way ANOVA	Ordinary
Alpha	0.05

Source of Variation	% of total variation	P value	P value summary	Significant?
Interaction	12.57	0.1329	ns	No
Row Factor	30.01	<0.0001	****	Yes
Column Factor	10.46	0.0114	*	Yes

Tukey's multiple comparisons test	Mean Diff.	95.00% CI of diff.	Significant?	Summary	Adjusted P Value
0h					
Control vs. Endurance	-0.1775	-1.358 to 1.003	No	ns	0.9784
Control vs. Interval	0.05775	-1.122 to 1.238	No	ns	0.9992
Control vs. Strength	0.0206	-1.092 to 1.133	No	ns	>0.9999
Endurance vs. Interval	0.2353	-1.009 to 1.479	No	ns	0.9586
Endurance vs. Strength	0.1981	-0.9820 to 1.378	No	ns	0.9705
Interval vs. Strength	-0.03715	-1.217 to 1.143	No	ns	0.9998
6h					
Control vs. Endurance	-0.08495	-1.265 to 1.095	No	ns	0.9975
Control vs. Interval	0.3367	-0.8435 to 1.517	No	ns	0.8742
Control vs. Strength	0.2733	-0.9069 to 1.453	No	ns	0.9276
Endurance vs. Interval	0.4216	-0.6911 to 1.534	No	ns	0.7484
Endurance vs. Strength	0.3582	-0.7545 to 1.471	No	ns	0.8293
Interval vs. Strength	-0.0634	-1.176 to 1.049	No	ns	0.9988
12h					
Control vs. Endurance	0.05905	-1.121 to 1.239	No	ns	0.9992
Control vs. Interval	-0.044	-1.157 to 1.069	No	ns	0.9996
Control vs. Strength	0.3388	-0.7739 to 1.451	No	ns	0.8514
Endurance vs. Interval	-0.1031	-1.283 to 1.077	No	ns	0.9956
Endurance vs. Strength	0.2798	-0.9004 to 1.460	No	ns	0.9229
Interval vs. Strength	0.3828	-0.7299 to 1.495	No	ns	0.7993
18h					
Control vs. Endurance	-0.4965	-1.740 to 0.7475	No	ns	0.7172
Control vs. Interval	1.2	0.01996 to 2.380	Yes	*	0.0448
Control vs. Strength	1.337	0.09277 to 2.581	Yes	*	0.0306
Endurance vs. Interval	1.697	0.5165 to 2.877	Yes	**	0.0019
Endurance vs. Strength	1.833	0.5893 to 3.077	Yes	**	0.0014
Interval vs. Strength	0.1367	-1.043 to 1.317	No	ns	0.9899

Figure 5 - Soleus

C

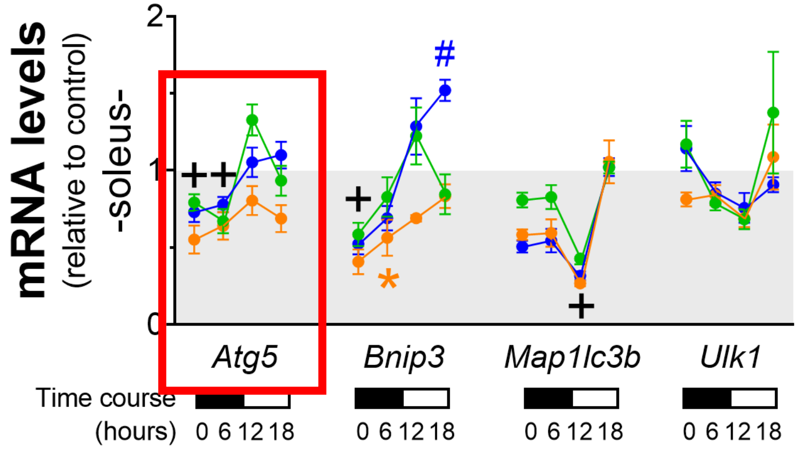

Atg5 - Soleus

	Control				Endurance				Interval				Strength			
	**0h**	**6h**	**12h**	**18h**	**0h**	**6h**	**12h**	**18h**	**0h**	**6h**	**12h**	**18h**	**0h**	**6h**	**12h**	**18h**
	1	0.617	0.642	0.593	0.629	0.567	0.804	0.532	0.306	0.411	0.512	0.381	0.615	0.482	0.62	0.751
	0.646	0.779	0.472	0.582	0.379	0.494	0.556	0.7	0.602	0.312	0.356	0.338	0.584	0.567	0.692	0.706
	0.632	0.654	0.689	0.635	0.615	0.312	0.685	0.473	0.335	0.675	0.502	0.34	0.412	0.401	0.553	0.686
	0.651	0.713	0.37	0.583	0.449	0.483	0.743	0.455	0.389	0.394	0.321	0.334	0.645	0.554	0.501	0.49
	0.762		0.452	0.498	0.553					0.423		0.597	0.439	0.447	0.4	0.546
Mean	0.7382	0.6908	0.525	0.5782	0.525	0.464	0.697	0.54	0.408	0.443	0.4228	0.398	0.539	0.4902	0.5532	0.6358
Std. Deviation	0.1553	0.07088	0.1349	0.04979	0.1082	0.108	0.1058	0.1116	0.1338	0.1368	0.09841	0.1129	0.1063	0.07046	0.1117	0.1118
Std. Error of Mean	0.06946	0.03544	0.06031	0.02227	0.04837	0.05399	0.05291	0.05581	0.06691	0.06116	0.04921	0.05047	0.04752	0.03151	0.04996	0.05002

Figure 5 - Soleus

C

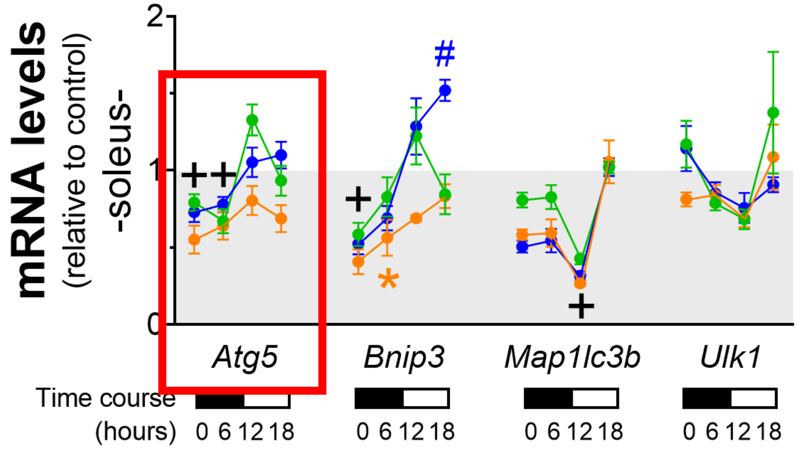

Two-way ANOVA	Ordinary
Alpha	0.05

Source of Variation	% of total variation	P value	P value summary	Significant?
Interaction	21.2	0.0087	**	Yes
Row Factor	0.7056	0.8407	ns	No
Column Factor	29.67	<0.0001	****	Yes

Tukey's multiple comparisons test	Mean Diff.	95.00% CI of diff.	Significant?	Summary	Adjusted P Value	
0h						
Control vs. Endurance	0.2132	0.02674 to 0.3997	Yes	*	0.0189	
Control vs. Interval	0.3302	0.1324 to 0.5280	Yes	***	0.0003	
Control vs. Strength	0.1992	0.01274 to 0.3857	Yes	*	0.0319	
Endurance vs. Interval	0.117	-0.08077 to 0.3148	No	ns	0.4064	
Endurance vs. Strength	-0.014	-0.2005 to 0.1725	No	ns	0.9972	
Interval vs. Strength	-0.131	-0.3288 to 0.06677	No	ns	0.3068	
6h						
Control vs. Endurance	0.2268	0.01829 to 0.4352	Yes	*	0.0279	
Control vs. Interval	0.2478	0.04998 to 0.4455	Yes	**	0.0084	
Control vs. Strength	0.2006	0.002785 to 0.3983	Yes	*	0.0457	
Endurance vs. Interval	0.021	-0.1768 to 0.2188	No	ns	0.9922	
Endurance vs. Strength	-0.0262	-0.2240 to 0.1716	No	ns	0.9851	
Interval vs. Strength	-0.0472	-0.2337 to 0.1393	No	ns	0.9081	
12h						
Control vs. Endurance	-0.172	-0.3698 to 0.02577	No	ns	0.1097	
Control vs. Interval	0.1023	-0.09552 to 0.3000	No	ns	0.5245	
Control vs. Strength	-0.0282	-0.2147 to 0.1583	No	ns	0.9781	
Endurance vs. Interval	0.2743	0.06579 to 0.4827	Yes	**	0.0052	*
Endurance vs. Strength	0.1438	-0.05397 to 0.3416	No	ns	0.2297	
Interval vs. Strength	-0.1305	-0.3282 to 0.06732	No	ns	0.3104	
18h						
Control vs. Endurance	0.0382	-0.1596 to 0.2360	No	ns	0.9562	
Control vs. Interval	0.1802	-0.006255 to 0.3667	No	ns	0.0618	
Control vs. Strength	-0.0576	-0.2441 to 0.1289	No	ns	0.8461	
Endurance vs. Interval	0.142	-0.05577 to 0.3398	No	ns	0.2397	
Endurance vs. Strength	-0.0958	-0.2936 to 0.1020	No	ns	0.5784	
Interval vs. Strength	-0.2378	-0.4243 to -0.05134	Yes	**	0.0071	*

* Data not plotted on the graph (the main aim was to highlight the differences in relation to control)

Figure 5 - Soleus

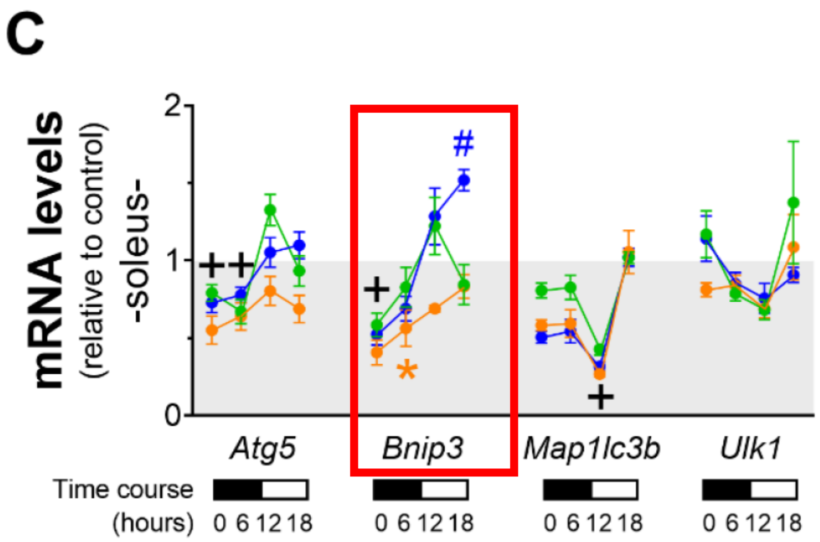

Bnip3 - Soleus

	Control				Endurance				Interval				Strength			
	**0h**	**6h**	**12h**	**18h**	**0h**	**6h**	**12h**	**18h**	**0h**	**6h**	**12h**	**18h**	**0h**	**6h**	**12h**	**18h**
	1	0.566	0.37	0.288	0.401	0.67	0.621	0.213	0.158	0.217	0.331	0.259	0.591	0.403	0.701	0.681
	0.749	0.794	0.416	0.373	0.317	0.558	0.33	0.345	0.319	0.327	0.291	0.195	0.392	0.515	0.795	0.548
	0.681	0.595	0.718	0.414	0.616	0.297	0.5	0.216	0.323	0.646	0.303	0.311	0.267	0.294	0.54	0.542
	0.584	0.615	0.283	0.322	0.361	0.604	0.704	0.384	0.456	0.267	0.291	0.321	0.401	0.59	0.389	0.519
	0.834		0.413	0.314	0.558					0.354		0.34	0.367	0.419	0.404	0.471
Mean	0.7696	0.6425	0.44	0.3422	0.4506	0.5323	0.5388	0.2895	0.314	0.3622	0.304	0.2852	0.4036	0.4442	0.5658	0.5522
Std. Deviation	0.1581	0.103	0.1644	0.05061	0.1296	0.1634	0.1624	0.08806	0.1219	0.1673	0.01887	0.05868	0.1175	0.1131	0.1795	0.07811
Std. Error of Mean	0.07069	0.05149	0.07353	0.02263	0.05798	0.08172	0.08122	0.04403	0.06097	0.07483	0.009434	0.02624	0.05256	0.05058	0.08029	0.03493

Figure 5 - Soleus

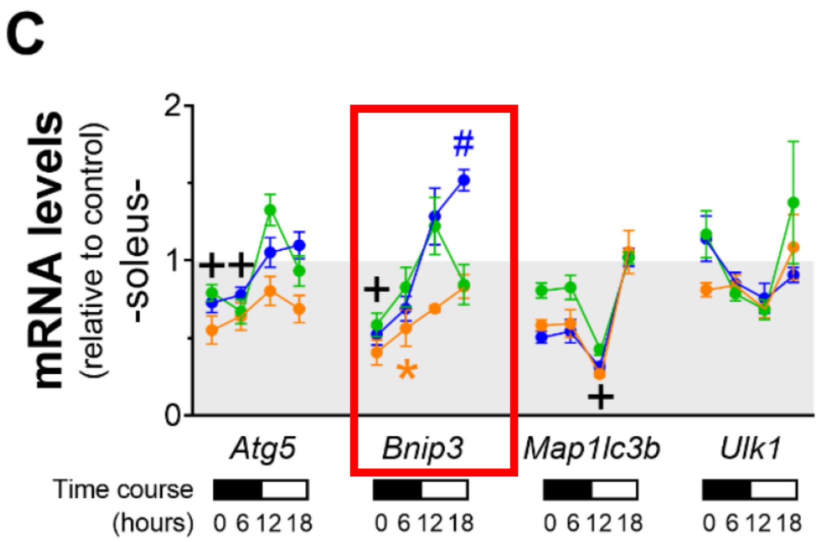

Two-way ANOVA	Ordinary
Alpha	0.05

Source of Variation	% of total variation	P value	P value summary	Significant?
Interaction	28.64	0.0002	***	Yes
Row Factor	8.361	0.0125	*	Yes
Column Factor	23.46	<0.0001	****	Yes

Tukey's multiple comparisons test	Mean Diff.	95.00% CI of diff.	Significant?	Summary	Adjusted P Value
0h					
Control vs. Endurance	0.319	0.1070 to 0.5310	Yes	**	0.0011
Control vs. Interval	0.4556	0.2308 to 0.6804	Yes	****	<0.0001
Control vs. Strength	0.366	0.1540 to 0.5780	Yes	***	0.0002
Endurance vs. Interval	0.1366	-0.08822 to 0.3614	No	ns	0.3827
Endurance vs. Strength	0.047	-0.1650 to 0.2590	No	ns	0.9358
Interval vs. Strength	-0.0896	-0.3144 to 0.1352	No	ns	0.7184
6h					
Control vs. Endurance	0.1103	-0.1267 to 0.3472	No	ns	0.6102
Control vs. Interval	0.2803	0.05548 to 0.5051	Yes	**	0.0088
Control vs. Strength	0.1983	-0.02652 to 0.4231	No	ns	0.1023
Endurance vs. Interval	0.1701	-0.05477 to 0.3949	No	ns	0.1996
Endurance vs. Strength	0.08805	-0.1368 to 0.3129	No	ns	0.7292
Interval vs. Strength	-0.082	-0.2940 to 0.1300	No	ns	0.7366
12h					
Control vs. Endurance	-0.09875	-0.3236 to 0.1261	No	ns	0.6531
Control vs. Interval	0.136	-0.08882 to 0.3608	No	ns	0.3866
Control vs. Strength	-0.1258	-0.3378 to 0.08616	No	ns	0.4036
Endurance vs. Interval	0.2348	-0.002232 to 0.4717	No	ns	0.0531
Endurance vs. Strength	-0.02705	-0.2519 to 0.1978	No	ns	0.9887
Interval vs. Strength	-0.2618	-0.4866 to -0.03698	Yes	*	0.0162 *
18h					
Control vs. Endurance	0.0527	-0.1721 to 0.2775	No	ns	0.9253
Control vs. Interval	0.057	-0.1550 to 0.2690	No	ns	0.8922
Control vs. Strength	-0.21	-0.4220 to 0.001963	No	ns	0.053
Endurance vs. Interval	0.0043	-0.2205 to 0.2291	No	ns	>0.9999
Endurance vs. Strength	-0.2627	-0.4875 to -0.03788	Yes	*	0.0158
Interval vs. Strength	-0.267	-0.4790 to -0.05504	Yes	**	0.008

* Data not plotted on the graph (the main aim was to highlight the differences in relation to control)

Figure 5 - Soleus

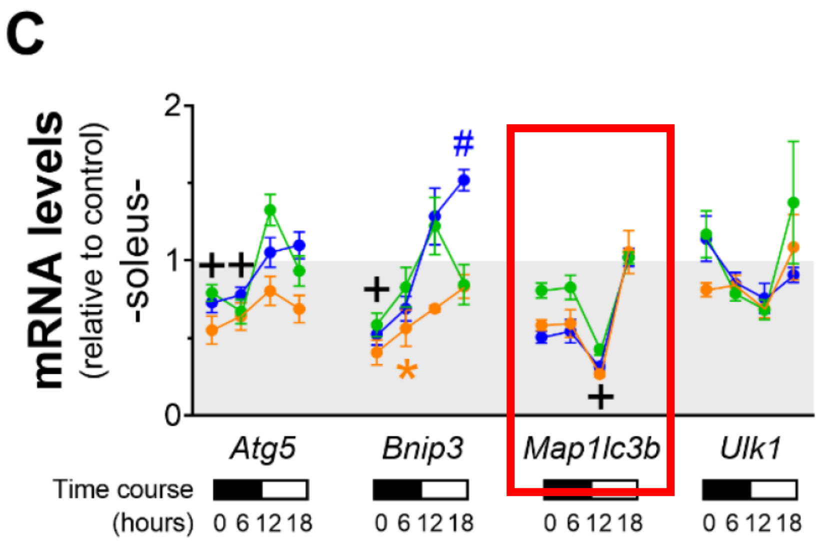

Map1lc3b - Soleus

	Control				Endurance				Interval				Strength			
	**0h**	**6h**	**12h**	**18h**	**0h**	**6h**	**12h**	**18h**	**0h**	**6h**	**12h**	**18h**	**0h**	**6h**	**12h**	**18h**
	1	0.646	0.988	0.454	0.606	0.433	0.575	0.416	0.336	0.337	0.378	0.331	0.362	0.256	0.357	0.401
	0.643	0.762	2.847	0.378	0.51	0.599	0.455	0.386	0.439	0.238	0.313	0.286	0.318	0.287	0.439	0.445
	0.592	0.453	1.017	0.436	0.577	0.407	0.409	0.451	0.396	0.558	0.286	0.401	0.268	0.482	0.357	0.472
	0.516	0.554	0.287	0.366	0.447	0.56	0.582	0.386	0.428	0.356	0.286	0.571	0.428	0.386	0.448	0.335
	0.672		0.75	0.376	0.626					0.306		0.535	0.359	0.236	0.268	0.402
Mean	0.6846	0.6038	1.178	0.402	0.5532	0.4998	0.5053	0.4098	0.3998	0.359	0.3158	0.4248	0.347	0.3294	0.3738	0.411
Std. Deviation	0.186	0.1317	0.9778	0.04002	0.07382	0.09405	0.08669	0.03092	0.04625	0.1199	0.04341	0.1246	0.05919	0.103	0.07334	0.05204
Std. Error of Mean	0.08318	0.06585	0.4373	0.0179	0.03301	0.04703	0.04334	0.01546	0.02312	0.05364	0.0217	0.05574	0.02647	0.04604	0.0328	0.02327

Figure 5 - Soleus

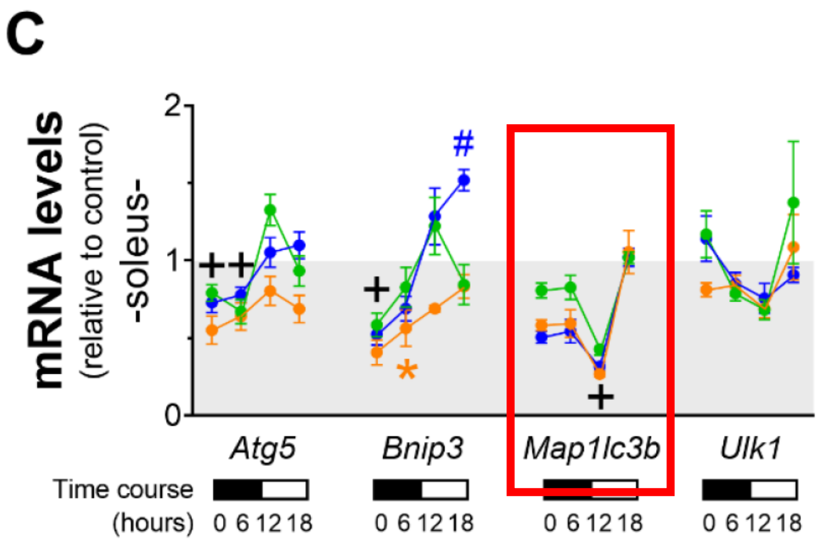

Two-way ANOVA	Ordinary
Alpha	0.05

Source of Variation	% of total variation	P value	P value summary	Significant?
Interaction	17.31	0.0603	ns	No
Row Factor	4.41	0.2238	ns	No
Column Factor	19.96	0.0005	***	Yes

Tukey's multiple comparisons test	Mean Diff.	95.00% CI of diff.	Significant?	Summary	Adjusted P Value
0h					
Control vs. Endurance	0.1314	-0.3249 to 0.5877	No	ns	0.8713
Control vs. Interval	0.2849	-0.1992 to 0.7689	No	ns	0.4111
Control vs. Strength	0.3376	-0.1187 to 0.7939	No	ns	0.2163
Endurance vs. Interval	0.1535	-0.3306 to 0.6375	No	ns	0.8359
Endurance vs. Strength	0.2062	-0.2501 to 0.6625	No	ns	0.6323
Interval vs. Strength	0.05275	-0.4313 to 0.5368	No	ns	0.9916
6h					
Control vs. Endurance	0.104	-0.4062 to 0.6142	No	ns	0.9491
Control vs. Interval	0.2448	-0.2393 to 0.7288	No	ns	0.5432
Control vs. Strength	0.2744	-0.2097 to 0.7584	No	ns	0.4445
Endurance vs. Interval	0.1408	-0.3433 to 0.6248	No	ns	0.868
Endurance vs. Strength	0.1704	-0.3137 to 0.6544	No	ns	0.7884
Interval vs. Strength	0.0296	-0.4267 to 0.4859	No	ns	0.9982
12h					
Control vs. Endurance	0.6726	0.1885 to 1.157	Yes	**	0.0029
Control vs. Interval	0.8621	0.3780 to 1.346	Yes	****	<0.0001
Control vs. Strength	0.804	0.3477 to 1.260	Yes	***	0.0001
Endurance vs. Interval	0.1895	-0.3207 to 0.6997	No	ns	0.7599
Endurance vs. Strength	0.1315	-0.3526 to 0.6155	No	ns	0.8894
Interval vs. Strength	-0.05805	-0.5421 to 0.4260	No	ns	0.9888
18h					
Control vs. Endurance	-0.00775	-0.4918 to 0.4763	No	ns	>0.9999
Control vs. Interval	-0.0228	-0.4791 to 0.4335	No	ns	0.9992
Control vs. Strength	-0.009	-0.4653 to 0.4473	No	ns	>0.9999
Endurance vs. Interval	-0.01505	-0.4991 to 0.4690	No	ns	0.9998
Endurance vs. Strength	-0.00125	-0.4853 to 0.4828	No	ns	>0.9999
Interval vs. Strength	0.0138	-0.4425 to 0.4701	No	ns	0.9998

Figure 5 - Soleus

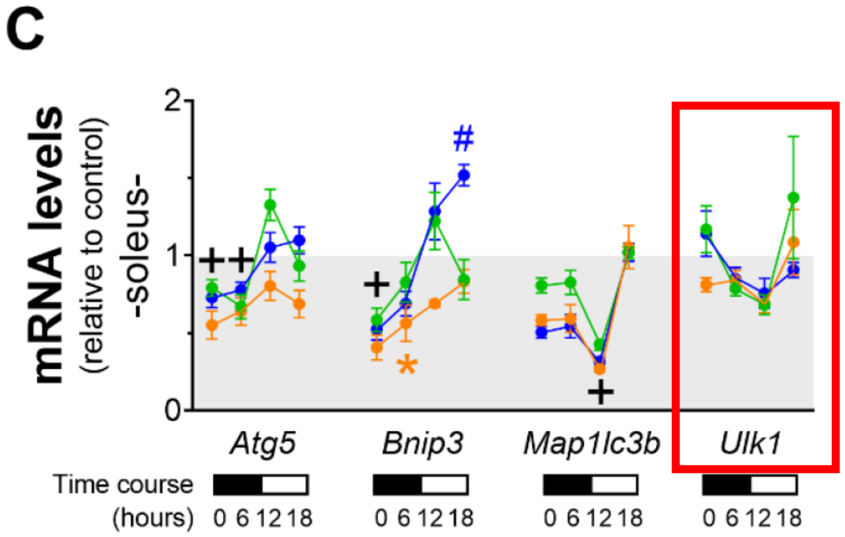

Ulk1 - Soleus

	Control				Endurance				Interval				Strength			
	0h	6h	12h	18h	0h	6h	12h	18h	0h	6h	12h	18h	0h	6h	12h	18h
	1	0.771	0.672	0.913	0.962	0.707	0.838	1.735	0.654	0.609	0.829	0.592	1.263	0.626	0.591	0.688
	0.736	0.831	2.472	0.685	0.838	0.558	0.547	1.071	0.702	0.574	0.586	0.643	0.672	0.684	0.8	0.759
	0.683	0.581	0.638	0.683	1.262	0.548	0.611	0.473	0.548	0.843	0.701	0.763	1.124	0.826	1.09	0.674
	0.814	0.883	0.25	0.698	0.691	0.607	0.686	1.46	0.701	0.599	0.595	0.571	0.754	0.643	0.62	0.544
	0.774		0.871	0.661	1.749					0.594		1.39	0.763	0.481	0.613	0.641
Mean	0.8014	0.7665	0.9806	0.728	1.1	0.605	0.6705	1.185	0.6513	0.6438	0.6778	0.7918	0.9152	0.652	0.7428	0.6612
Std. Deviation	0.1211	0.1319	0.8635	0.1043	0.4191	0.07272	0.1253	0.5471	0.07238	0.1121	0.1136	0.3426	0.2612	0.1238	0.2114	0.07841
Std. Error of Mean	0.05415	0.06593	0.3862	0.04663	0.1874	0.03636	0.06264	0.2736	0.03619	0.05013	0.05678	0.1532	0.1168	0.05536	0.09455	0.03506

Figure 5 - Soleus

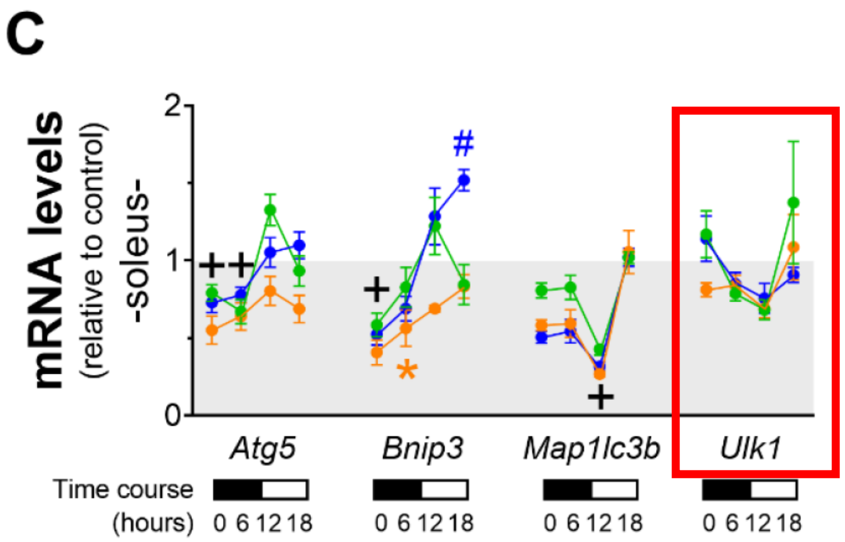

Two-way ANOVA	Ordinary
Alpha	0.05

Source of Variation	% of total variation	P value	P value summary	Significant?
Interaction	14.5	0.2831	ns	No
Row Factor	5.553	0.2411	ns	No
Column Factor	5.056	0.2802	ns	No

Figure 5 - Hippocampus

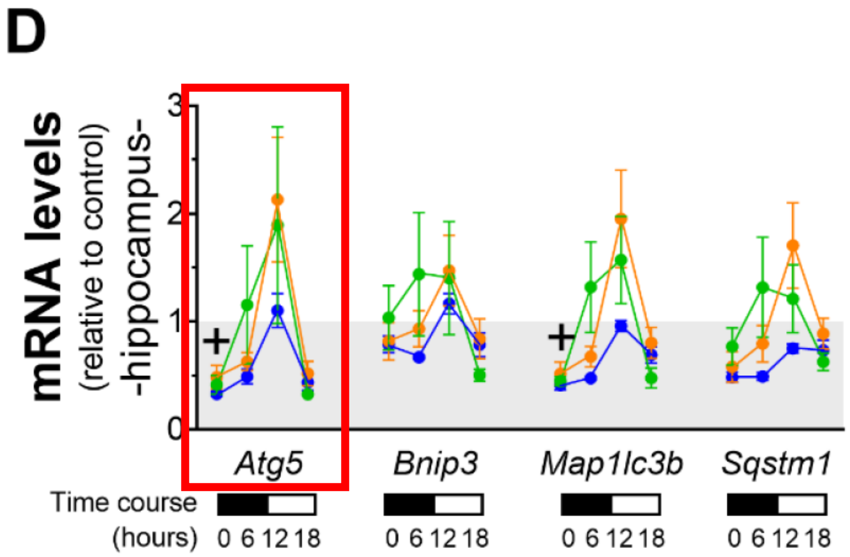

Atg5 - Hippocampus

	Control				Endurance				Interval				Strength			
	0h	6h	12h	18h	0h	6h	12h	18h	0h	6h	12h	18h	0h	6h	12h	18h
	1	0.27	0.12	0.351	0.101	0.195	0.089	0.123	0.319	0.14	0.272	0.108	0.113	0.179	0.095	0.108
	0.544	0.569	0.143	0.156	0.189	0.618	0.124	0.147	0.188	0.209	0.104	0.334	0.113	0.169	0.177	0.18
	0.143	0.096	0.084	0.132	0.253	0.142	0.554	0.134	0.129	0.144	0.503	0.146	0.134	0.123	0.17	0.102
	0.219	0.168	0.204	0.192	0.121		0.142	0.088	0.14	0.245	0.253	0.141	0.138	0.075	0.134	0.244
			0.197					0.079		0.129	0.147	0.179	0.162	0.128	0.085	0.131
Mean	0.4765	0.2758	0.1496	0.2078	0.166	0.3183	0.2273	0.1142	0.194	0.1734	0.2558	0.1816	0.132	0.1348	0.1322	0.153
Std. Deviation	0.3899	0.2081	0.05106	0.09863	0.06916	0.2609	0.2189	0.02946	0.08718	0.05086	0.1551	0.08883	0.02038	0.04149	0.04198	0.05941
Std. Error of Mean	0.195	0.1041	0.02284	0.04932	0.03458	0.1506	0.1095	0.01317	0.04359	0.02274	0.06938	0.03973	0.009116	0.01855	0.01878	0.02657

Figure 5 - Hippocampus

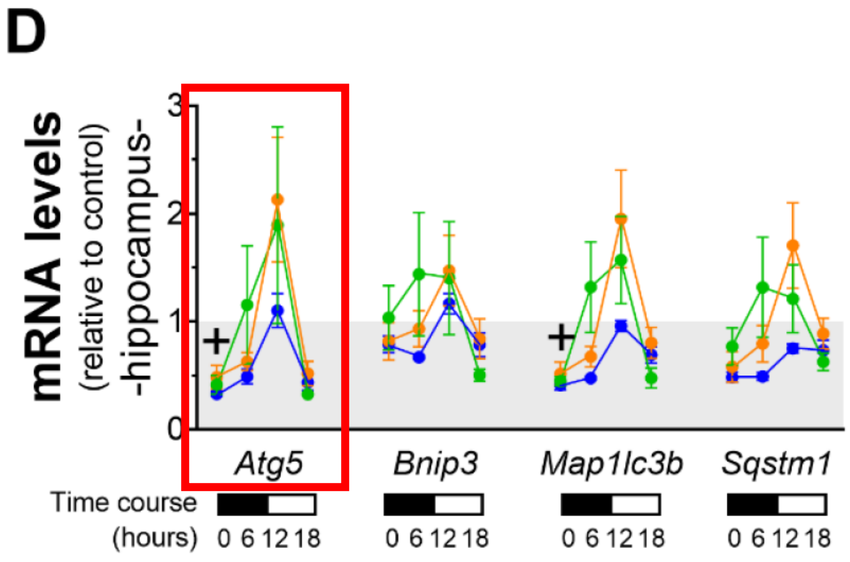

Two-way ANOVA	Ordinary			
Alpha	0.05			
Source of Variation	% of total variation	P value	P value summary	Significant?
Interaction	18.99	0.101	ns	No
Row Factor	3.963	0.361	ns	No
Column Factor	10.84	0.0389	*	Yes

Tukey's multiple comparisons test	Mean Diff.	95.00% CI of diff.	Significant?	Summary	Adjusted P Value
0h					
Control vs. Endurance	0.3105	0.04641 to 0.5746	Yes	*	0.015
Control vs. Interval	0.2825	0.01841 to 0.5466	Yes	*	0.0316
Control vs. Strength	0.3445	0.09396 to 0.5950	Yes	**	0.0032
Endurance vs. Interval	-0.028	-0.2921 to 0.2361	No	ns	0.9922
Endurance vs. Strength	0.034	-0.2165 to 0.2845	No	ns	0.9839
Interval vs. Strength	0.062	-0.1885 to 0.3125	No	ns	0.9133
6h					
Control vs. Endurance	-0.04258	-0.3278 to 0.2427	No	ns	0.9789
Control vs. Interval	0.1024	-0.1482 to 0.3529	No	ns	0.702
Control vs. Strength	0.141	-0.1096 to 0.3915	No	ns	0.4505
Endurance vs. Interval	0.1449	-0.1278 to 0.4177	No	ns	0.5004
Endurance vs. Strength	0.1835	-0.08922 to 0.4563	No	ns	0.2928
Interval vs. Strength	0.0386	-0.1976 to 0.2748	No	ns	0.9726
12h					
Control vs. Endurance	-0.07765	-0.3282 to 0.1729	No	ns	0.8445
Control vs. Interval	-0.1062	-0.3424 to 0.1300	No	ns	0.6353
Control vs. Strength	0.0174	-0.2188 to 0.2536	No	ns	0.9973
Endurance vs. Interval	-0.02855	-0.2791 to 0.2220	No	ns	0.9904
Endurance vs. Strength	0.09505	-0.1555 to 0.3456	No	ns	0.7474
Interval vs. Strength	0.1236	-0.1126 to 0.3598	No	ns	0.5136
18h					
Control vs. Endurance	0.09355	-0.1570 to 0.3441	No	ns	0.7564
Control vs. Interval	0.02615	-0.2244 to 0.2767	No	ns	0.9925
Control vs. Strength	0.05475	-0.1958 to 0.3053	No	ns	0.9381
Endurance vs. Interval	-0.0674	-0.3036 to 0.1688	No	ns	0.8739
Endurance vs. Strength	-0.0388	-0.2750 to 0.1974	No	ns	0.9722
Interval vs. Strength	0.0286	-0.2076 to 0.2648	No	ns	0.9885

Figure 5 - Hippocampus

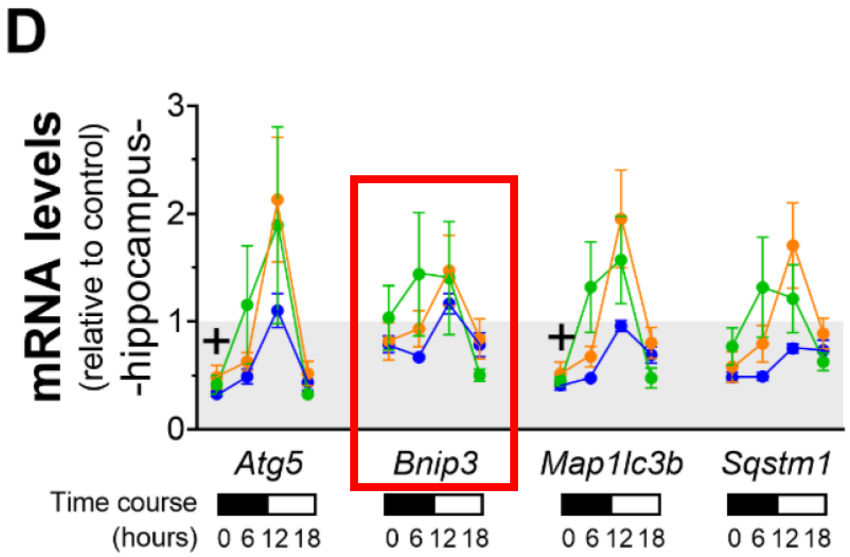

Bnip3 - Hippocampus

	Control				Endurance				Interval				Strength			
	**0h**	**6h**	**12h**	**18h**	**0h**	**6h**	**12h**	**18h**	**0h**	**6h**	**12h**	**18h**	**0h**	**6h**	**12h**	**18h**
	1	0.269	0.26	0.494	0.28	0.376	0.174	0.224	0.518	0.231	0.609	0.243	0.229	0.248	0.238	0.322
	0.166	0.653	0.248	0.435	0.312	0.896	0.288	0.35	0.219	0.26	0.194	0.771	0.269	0.227	0.325	0.513
	0.09	0.228	0.22	0.381	0.751	0.239	0.767	0.243	0.268	0.223	0.522	0.326	0.287	0.249	0.354	0.232
	0.302	0.249	0.282	0.463	0.27		0.229	0.234	0.268	0.53	0.401	0.388	0.357	0.214	0.346	0.5
			0.289					0.188		0.387	0.188	0.346	0.393	0.231	0.251	0.372
Mean	0.3895	0.3498	0.2598	0.4433	0.4033	0.5037	0.3645	0.2478	0.3183	0.3262	0.3828	0.4148	0.307	0.2338	0.3028	0.3878
Std. Deviation	0.4163	0.2029	0.0277	0.04799	0.2325	0.3466	0.2723	0.06083	0.1352	0.1316	0.19	0.206	0.06675	0.01482	0.05446	0.1195
Std. Error of Mean	0.2082	0.1014	0.01239	0.02399	0.1163	0.2001	0.1362	0.0272	0.06758	0.05888	0.08499	0.09212	0.02985	0.006629	0.02435	0.05344

Figure 5 - Hippocampus

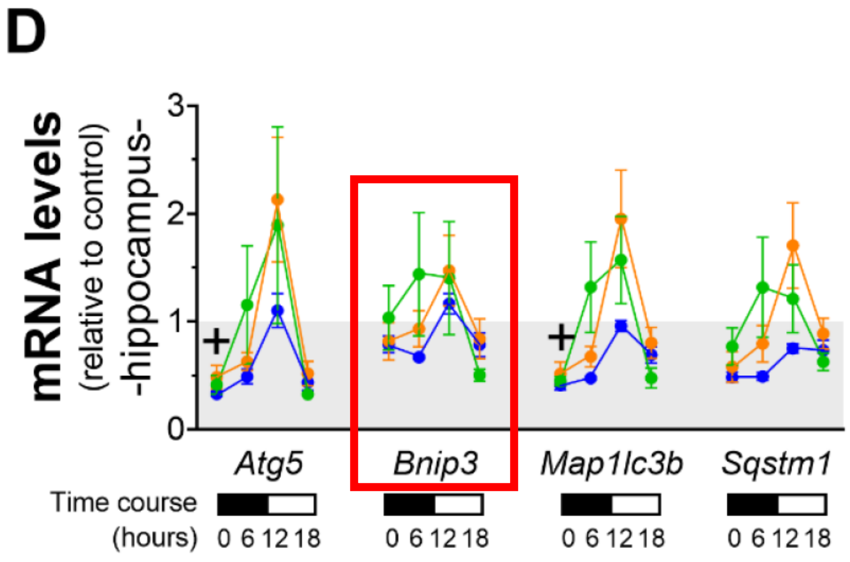

Two-way ANOVA	Ordinary
Alpha	0.05

Source of Variation	% of total variation	P value	P value summary	Significant?
Interaction	13.07	0.4767	ns	No
Row Factor	0.9286	0.8918	ns	No
Column Factor	2.462	0.6526	ns	No

Figure 5 - Hippocampus

D

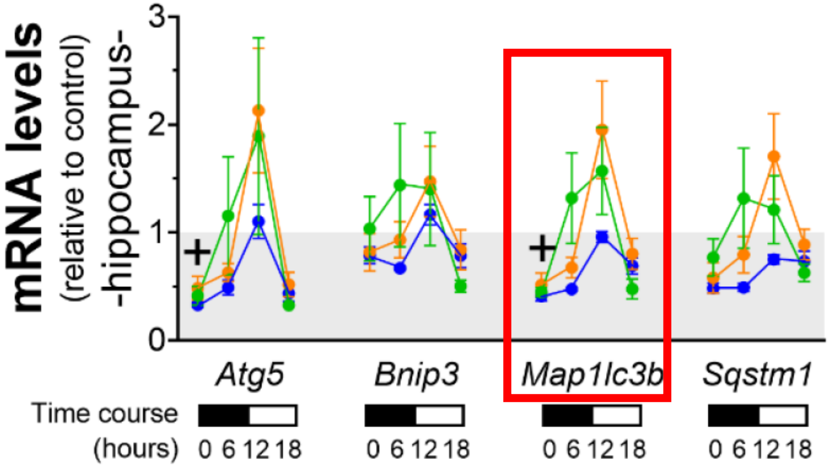

Map1lc3b - Hippocampus

	Control				Endurance				Interval				Strength			
	0h	6h	12h	18h	0h	6h	12h	18h	0h	6h	12h	18h	0h	6h	12h	18h
	1	0.212	0.172	0.298	0.139	0.361	0.154	0.141	0.362	0.14	0.374	0.176	0.142	0.131	0.152	0.165
	0.39	0.609	0.163	0.26	0.217	0.626	0.202	0.19	0.173	0.193	0.171	0.39	0.13	0.135	0.174	0.246
	0.29	0.191	0.208	0.198	0.211	0.196	0.469	0.209	0.161	0.171	0.538	0.242	0.172	0.148	0.165	0.149
	0.214	0.184	0.218	0.256	0.206		0.256	0.054	0.197	0.308	0.455	0.138	0.214	0.141	0.192	0.262
			0.141					0.115		0.197	0.142	0.248	0.236	0.154	0.141	0.206
Mean	0.4735	0.299	0.1804	0.253	0.1933	0.3943	0.2703	0.1418	0.2233	0.2018	0.336	0.2388	0.1788	0.1418	0.1648	0.2056
Std. Deviation	0.3583	0.207	0.03202	0.04126	0.03645	0.2169	0.1389	0.06178	0.0937	0.06354	0.1741	0.09626	0.04553	0.009365	0.01972	0.04916
Std. Error of Mean	0.1792	0.1035	0.01432	0.02063	0.01822	0.1252	0.06945	0.02763	0.04685	0.02841	0.07787	0.04305	0.02036	0.004188	0.008817	0.02198

Figure 5 - Hippocampus

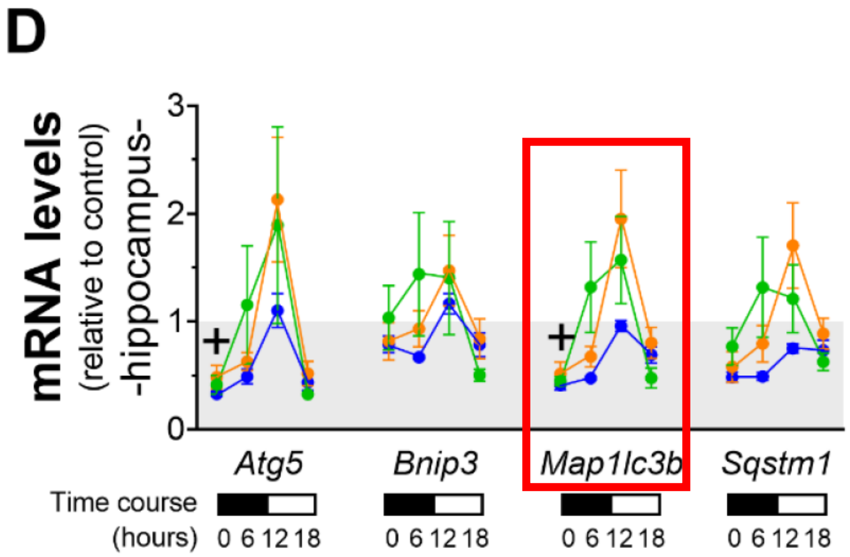

Two-way ANOVA	Ordinary
Alpha	0.05

Source of Variation	% of total variation	P value	P value summary	Significant?
Interaction	25.22	0.018	*	Yes
Row Factor	2.436	0.5434	ns	No
Column Factor	10.85	0.0297	*	Yes

Tukey's multiple comparisons test	Mean Diff.	95.00% CI of diff.	Significant?	Summary	Adjusted P Value	
0h						
Control vs. Endurance	0.2803	0.04093 to 0.5196	Yes	*	0.0155	
Control vs. Interval	0.2503	0.01093 to 0.4896	Yes	*	0.0371	
Control vs. Strength	0.2947	0.06766 to 0.5217	Yes	**	0.006	
Endurance vs. Interval	-0.03	-0.2693 to 0.2093	No	ns	0.9873	
Endurance vs. Strength	0.01445	-0.2126 to 0.2415	No	ns	0.9983	
Interval vs. Strength	0.04445	-0.1826 to 0.2715	No	ns	0.9543	
6h						
Control vs. Endurance	-0.09533	-0.3538 to 0.1632	No	ns	0.7633	
Control vs. Interval	0.0972	-0.1298 to 0.3242	No	ns	0.6705	
Control vs. Strength	0.1572	-0.06984 to 0.3842	No	ns	0.2688	
Endurance vs. Interval	0.1925	-0.05463 to 0.4397	No	ns	0.1781	
Endurance vs. Strength	0.2525	0.005368 to 0.4997	Yes	*	0.0435	*
Interval vs. Strength	0.06	-0.1541 to 0.2741	No	ns	0.8796	
12h						
Control vs. Endurance	-0.08985	-0.3169 to 0.1372	No	ns	0.7222	
Control vs. Interval	-0.1556	-0.3697 to 0.05845	No	ns	0.2295	
Control vs. Strength	0.0156	-0.1985 to 0.2297	No	ns	0.9974	
Endurance vs. Interval	-0.06575	-0.2928 to 0.1613	No	ns	0.869	
Endurance vs. Strength	0.1055	-0.1216 to 0.3325	No	ns	0.6108	
Interval vs. Strength	0.1712	-0.04285 to 0.3853	No	ns	0.16	
18h						
Control vs. Endurance	0.1112	-0.1158 to 0.3382	No	ns	0.5687	
Control vs. Interval	0.0142	-0.2128 to 0.2412	No	ns	0.9984	
Control vs. Strength	0.0474	-0.1796 to 0.2744	No	ns	0.9454	
Endurance vs. Interval	-0.097	-0.3111 to 0.1171	No	ns	0.6295	
Endurance vs. Strength	-0.0638	-0.2779 to 0.1503	No	ns	0.859	
Interval vs. Strength	0.0332	-0.1809 to 0.2473	No	ns	0.9764	

* Data not plotted on the graph (the main aim was to highlight the differences in relation to control)

Figure 5 - Hippocampus

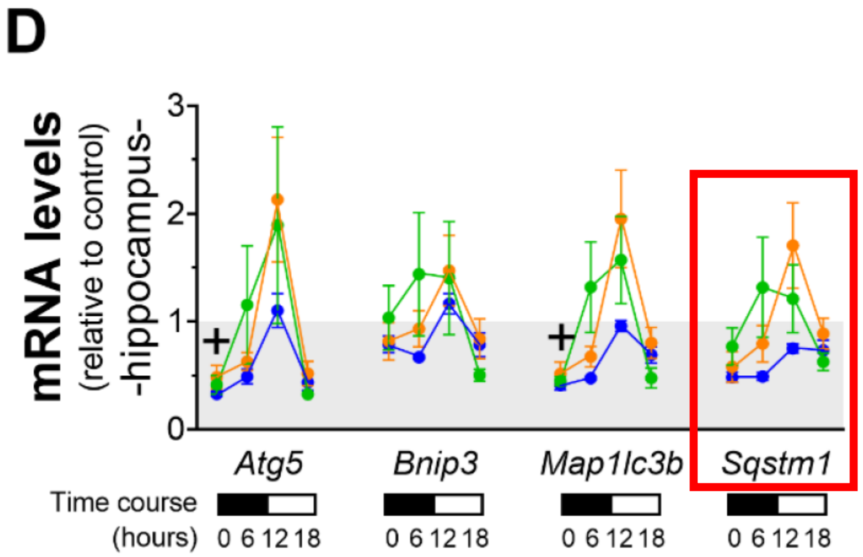

Sqstm1 - Hippocampus

	Control				Endurance				Interval				Strength			
	0h	6h	12h	18h	0h	6h	12h	18h	0h	6h	12h	18h	0h	6h	12h	18h
	1	0.207	0.263	0.368	0.192	0.4	0.172	0.193	0.468	0.176	0.512	0.231	0.161	0.154	0.163	0.205
	0.468	0.761	0.171	0.345	0.436	0.762	0.282	0.318	0.19	0.206	0.23	0.53	0.195	0.158	0.211	0.326
	0.17	0.22	0.206	0.262	0.545	0.213	0.556	0.274	0.2	0.226	0.581	0.283	0.245	0.21	0.193	0.185
	0.233	0.203	0.262	0.379	0.265		0.264	0.218	0.226	0.504	0.731	0.277	0.244	0.14	0.228	0.367
			0.265					0.149		0.268	0.188	0.312				
Mean	0.4678	0.3478	0.2334	0.3385	0.3595	0.4583	0.3185	0.2304	0.271	0.276	0.4484	0.3266	0.2113	0.1655	0.1988	0.2708
Std. Deviation	0.3773	0.2756	0.04283	0.05293	0.1605	0.2791	0.1655	0.06662	0.1322	0.1318	0.2329	0.1174	0.04083	0.03065	0.02779	0.08943
Std. Error of Mean	0.1886	0.1378	0.01915	0.02647	0.08023	0.1611	0.08275	0.02979	0.0661	0.05893	0.1042	0.05248	0.02041	0.01533	0.01389	0.04471

Figure 5 - Hippocampus

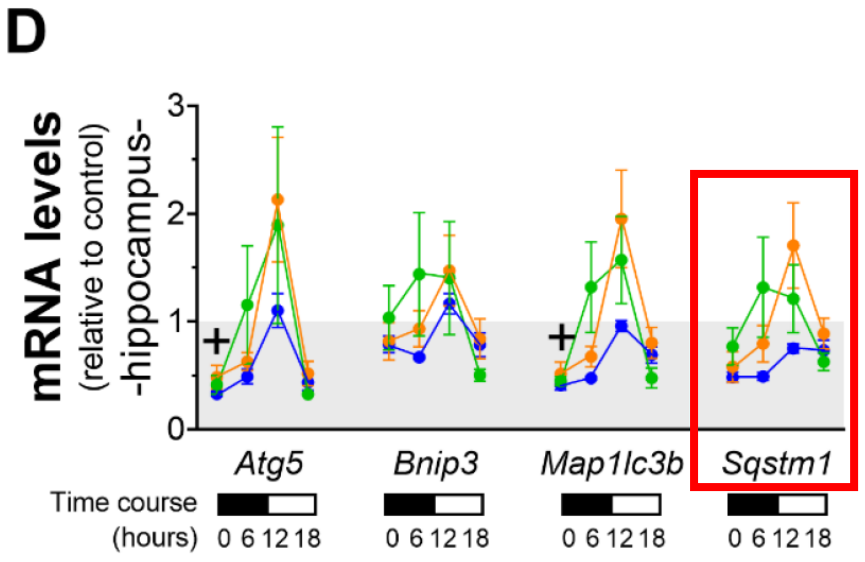

Two-way ANOVA	Ordinary
Alpha	0.05

Source of Variation	% of total variation	P value	P value summary	Significant?
Interaction	16.82	0.1896	ns	No
Row Factor	0.8047	0.8904	ns	No
Column Factor	11.16	0.0436	*	Yes

Tukey's multiple comparisons test	Mean Diff.	95.00% CI of diff.	Significant?	Summary	Adjusted P Value
0h					
Control vs. Endurance	0.1083	-0.1916 to 0.4081	No	ns	0.7748
Control vs. Interval	0.1968	-0.1031 to 0.4966	No	ns	0.3145
Control vs. Strength	0.2422	-0.04233 to 0.5266	No	ns	0.1215
Endurance vs. Interval	0.0885	-0.2114 to 0.3884	No	ns	0.8625
Endurance vs. Strength	0.1339	-0.1506 to 0.4184	No	ns	0.6005
Interval vs. Strength	0.0454	-0.2391 to 0.3299	No	ns	0.9744
6h					
Control vs. Endurance	-0.1106	-0.4345 to 0.2133	No	ns	0.8027
Control vs. Interval	0.07175	-0.2127 to 0.3562	No	ns	0.9088
Control vs. Strength	0.1772	-0.1073 to 0.4616	No	ns	0.3602
Endurance vs. Interval	0.1823	-0.1274 to 0.4920	No	ns	0.4101
Endurance vs. Strength	0.2877	-0.02197 to 0.5974	No	ns	0.0776
Interval vs. Strength	0.1054	-0.1628 to 0.3736	No	ns	0.7265
12h					
Control vs. Endurance	-0.0851	-0.3696 to 0.1994	No	ns	0.8577
Control vs. Interval	-0.215	-0.4832 to 0.05321	No	ns	0.1585
Control vs. Strength	0.0362	-0.2320 to 0.3044	No	ns	0.9842
Endurance vs. Interval	-0.1299	-0.4144 to 0.1546	No	ns	0.6238
Endurance vs. Strength	0.1213	-0.1632 to 0.4058	No	ns	0.6733
Interval vs. Strength	0.2512	-0.01701 to 0.5194	No	ns	0.0742
18h					
Control vs. Endurance	0.1081	-0.1764 to 0.3926	No	ns	0.7465
Control vs. Interval	0.0119	-0.2726 to 0.2964	No	ns	0.9995
Control vs. Strength	0.0691	-0.2154 to 0.3536	No	ns	0.9175
Endurance vs. Interval	-0.0962	-0.3644 to 0.1720	No	ns	0.7782
Endurance vs. Strength	-0.039	-0.3072 to 0.2292	No	ns	0.9804
Interval vs. Strength	0.0572	-0.2110 to 0.3254	No	ns	0.9421

Figure 5 - Hippocampus

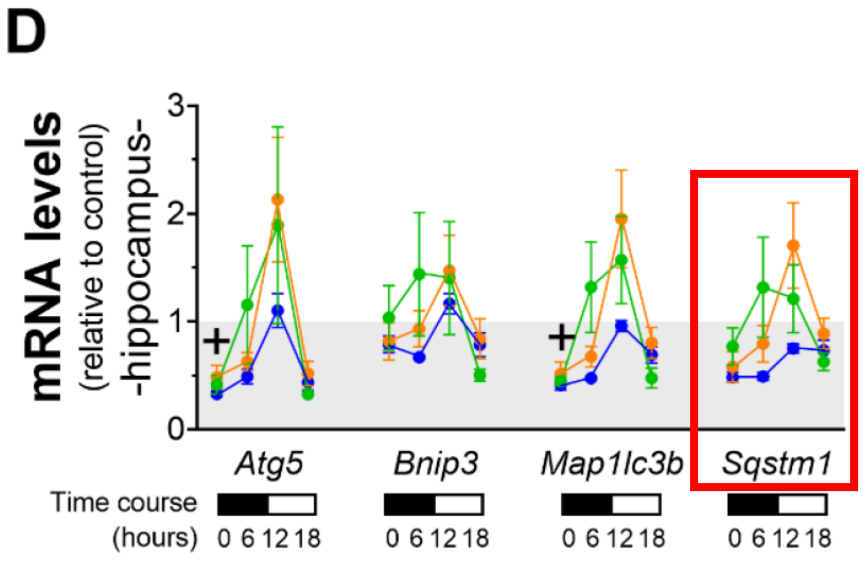

Two-way ANOVA	Ordinary
Alpha	0.05

Source of Variation	% of total variation	P value	P value summary	Significant?
Interaction	16.82	0.1896	ns	No
Row Factor	0.8047	0.8904	ns	No
Column Factor	11.16	0.0436	*	Yes

Tukey's multiple comparisons test	Mean Diff.	95.00% CI of diff.	Significant?	Summary	Adjusted P Value
0h					
Control vs. Endurance	0.1083	-0.1916 to 0.4081	No	ns	0.7748
Control vs. Interval	0.1968	-0.1031 to 0.4966	No	ns	0.3145
Control vs. Strength	0.2422	-0.04233 to 0.5266	No	ns	0.1215
Endurance vs. Interval	0.0885	-0.2114 to 0.3884	No	ns	0.8625
Endurance vs. Strength	0.1339	-0.1506 to 0.4184	No	ns	0.6005
Interval vs. Strength	0.0454	-0.2391 to 0.3299	No	ns	0.9744
6h					
Control vs. Endurance	-0.1106	-0.4345 to 0.2133	No	ns	0.8027
Control vs. Interval	0.07175	-0.2127 to 0.3562	No	ns	0.9088
Control vs. Strength	0.1772	-0.1073 to 0.4616	No	ns	0.3602
Endurance vs. Interval	0.1823	-0.1274 to 0.4920	No	ns	0.4101
Endurance vs. Strength	0.2877	-0.02197 to 0.5974	No	ns	0.0776
Interval vs. Strength	0.1054	-0.1628 to 0.3736	No	ns	0.7265
12h					
Control vs. Endurance	-0.0851	-0.3696 to 0.1994	No	ns	0.8577
Control vs. Interval	-0.215	-0.4832 to 0.05321	No	ns	0.1585
Control vs. Strength	0.0362	-0.2320 to 0.3044	No	ns	0.9842
Endurance vs. Interval	-0.1299	-0.4144 to 0.1546	No	ns	0.6238
Endurance vs. Strength	0.1213	-0.1632 to 0.4058	No	ns	0.6733
Interval vs. Strength	0.2512	-0.01701 to 0.5194	No	ns	0.0742
18h					
Control vs. Endurance	0.1081	-0.1764 to 0.3926	No	ns	0.7465
Control vs. Interval	0.0119	-0.2726 to 0.2964	No	ns	0.9995
Control vs. Strength	0.0691	-0.2154 to 0.3536	No	ns	0.9175
Endurance vs. Interval	-0.0962	-0.3644 to 0.1720	No	ns	0.7782
Endurance vs. Strength	-0.039	-0.3072 to 0.2292	No	ns	0.9804
Interval vs. Strength	0.0572	-0.2110 to 0.3254	No	ns	0.9421

Figure 6 - Gastrocnemius

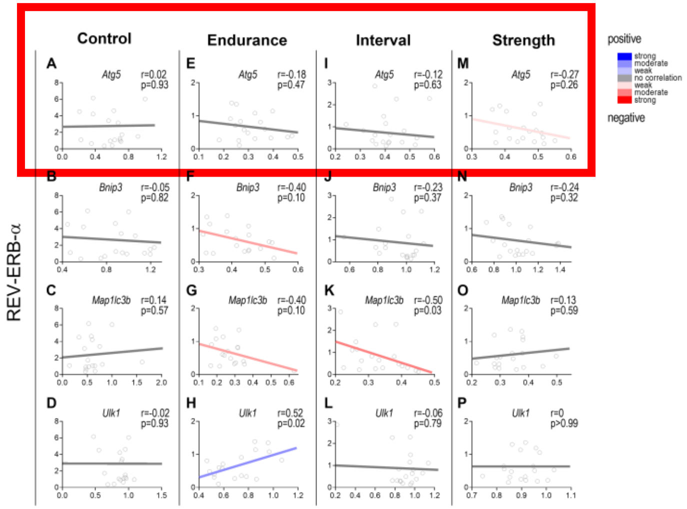

	Control	
	Atg5	Rev-erb-α
	1	6,038635
	0,371	6,156449
	0,558	3,403044
	0,622	4,626273
	0,206	4,518728
	0,507	0,353675
	0,695	1,232309
	0,633	1,082445
	0,37	4,212334
	0,579	0,947484
	0,696	1,0407
	0,914	1,509728
	0,602	0,408426
	0,225	1,093662
	0,673	3,147474
	0,719	1,726931
	0,352	0,641692
	0,707	0,912632
Mean	0,5794	2,392
Std. Deviation	0,2136	1,957
Std. Error of Mean	0,05033	0,4613
**Shapiro-Wilk test**		
W	0,9559	0,8467
P value	0,5256	0,0075
Passed normality test (alpha=0.05)?	Yes	No
P value summary	ns	**
**Spearman r**		
r	0,02167	
95% confidence interval	-0,4616 to 0,4950	
**P value**		
P (two-tailed)	0,932	
P value summary	ns	
Exact or approximate P value?	Approximate	
Significant? (alpha = 0.05)	No	

Figure 6 - Gastrocnemius

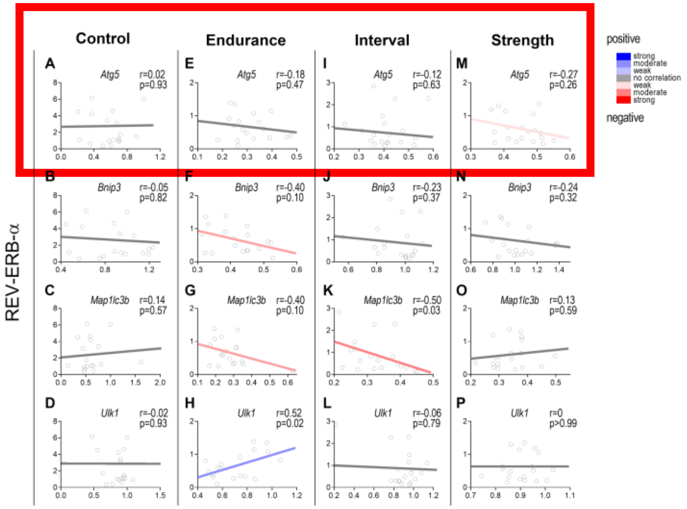

Endurance

Atg5	Rev-erb-α
0.397	1.141376
0.326	1.079966
0.268	0.827383
0.251	1.350847
0.247	0.705071
0.475	0.397243
0.261	0.2512
0.263	0.107043
0.182	0.269395
0.326	0.310628
0.458	0.355159
0.282	0.579293
0.39	0.327522
0.297	0.485072
0.227	0.90696
0.366	0.604437
0.348	0.809654
0.232	1.389225

	Atg5	Rev-erb-α
Mean	0.3109	0.661
Std. Deviation	0.08128	0.3904
Std. Error of Mean	0.01916	0.09201
**Shapiro-Wilk test**		
W	0.949	0.9357
P value	0.4099	0.2448
Passed normality test (alpha=0.05)?	Yes	Yes
P value summary	ns	ns
**Pearson r**		
r	-0.1797	
95% confidence interval	-0.5966 to 0.3134	
R squared	0.03231	
**P value**		
P (two-tailed)	0.4754	
P value summary	ns	
Significant? (alpha = 0.05)	No	

Interval

Atg5	Rev-erb-α
0.243	1.107878
0.395	1.480256
0.477	0.817224
0.364	0.215832
0.416	0.31058
0.349	0.380436
0.404	0.134215
0.392	0.310679
0.414	0.265282
0.469	0.304671
0.522	0.181579
0.528	0.492281
0.313	0.831089
0.421	0.651651
0.36	0.631346
0.403	1.149272
0.579	2.28466
0.446	2.263987
0.359	2.843293

	Atg5	Rev-erb-α
Mean	0.4134	0.8766
Std. Deviation	0.07928	0.8024
Std. Error of Mean	0.01819	0.1841
**Shapiro-Wilk test**		
W	0.9758	0.8166
P value	0.8837	0.002
Passed normality test (alpha=0.05)?	Yes	No
P value summary	ns	**
**Spearman r**		
r	-0.1193	
95% confidence interval	-0.5541 to 0.3667	
**P value**		
P (two-tailed)	0.6266	
P value summary	ns	
Exact or approximate P value?	Approximate	
Significant? (alpha = 0.05)	No	

Strength

Atg5	Rev-erb-α
0.413	0.642064
0.489	1.054293
0.372	0.508819
0.414	0.314392
0.456	0.639348
0.438	0.215245
0.372	0.188228
0.518	0.152135
0.551	0.249416
0.494	0.557413
0.511	0.480171
0.454	0.323525
0.489	0.216541
0.474	0.530745
0.515	0.345418
0.362	1.191836
0.507	1.361035
0.384	1.303525
0.404	1.135713

	Atg5	Rev-erb-α
Mean	0.4535	0.6005
Std. Deviation	0.05801	0.4056
Std. Error of Mean	0.01331	0.09305
**Shapiro-Wilk test**		
W	0.9407	0.8629
P value	0.2711	0.011
Passed normality test (alpha=0.05)?	Yes	No
P value summary	ns	*
**Spearman r**		
r	-0.2695	
95% confidence interval	-0.6532 to 0.2242	
**P value**		
P (two-tailed)	0.2645	
P value summary	ns	
Exact or approximate P value?	Approximate	
Significant? (alpha = 0.05)	No	

Figure 6 - Gastrocnemius

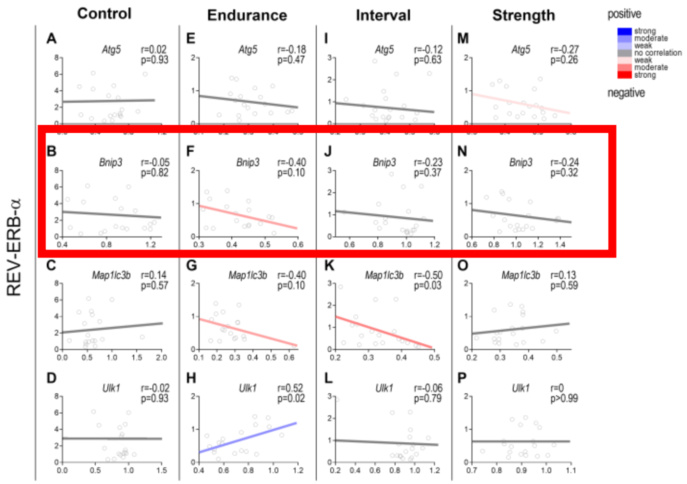

Control	
Bnip3	Rev-erb-α
1	6,038635
0,629	6,156449
0,933	3,403044
0,88	4,626273
0,448	4,518728
0,567	0,353675
0,885	1,232309
0,945	1,082445
0,591	4,212334
0,984	0,947484
1,146	1,0407
1,198	1,509728
0,748	0,408426
0,472	1,093662
1,061	3,147474
1,234	1,726931
0,784	0,641692
1,198	0,912632

	Bnip3	Rev-erb-α
Mean	0,8724	2,392
Std. Deviation	0,2528	1,957
Std. Error of Mean	0,05959	0,4613
**Shapiro-Wilk test**		
W	0,9448	0,8467
P value	0,3489	0,0075
Passed normality test (alpha=0.05)?	Yes	No
P value summary	ns	**
**Spearman r**		
r	-0,05679	
95% confidence interval	-0,5211 to 0,4335	
**P value**		
P (two-tailed)	0,8229	
P value summary	ns	
Exact or approximate P value?	Approximate	
Significant? (alpha = 0.05)	No	

Figure 6 - Gastrocnemius

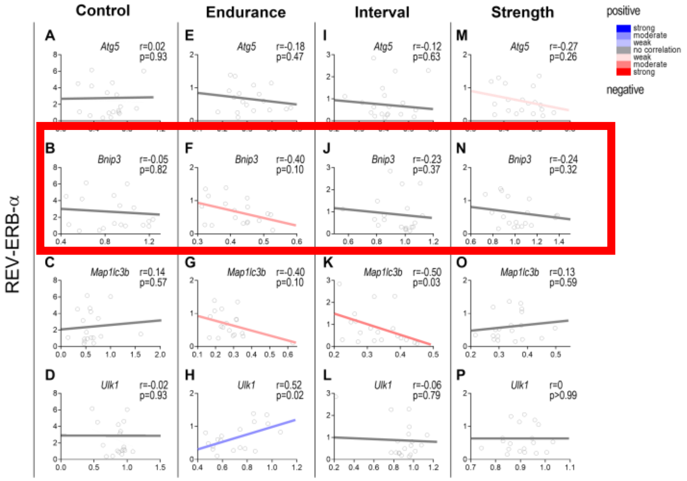

Endurance Bnip3	Endurance Rev-erb-α	Interval Bnip3	Interval Rev-erb-α	Strength Bnip3	Strength Rev-erb-α
0.333	1.141376	0.562	1.107878	0.791	0.642064
0.45	1.079966	0.805	1.480256	1.129	1.054293
0.313	0.827383	1.122	0.817224	0.896	0.508819
0.324	1.350847	1.004	0.215832	1.094	0.314392
0.411	0.705071	1.046	0.31058	1.113	0.639348
0.486	0.397243	0.81	0.380436	1.062	0.215245
0.343	0.2512	1.033	0.134215	0.994	0.188228
0.527	0.107043	0.973	0.310679	0.85	0.152135
0.322	0.269395	1.006	0.265282	1.149	0.249416
0.438	0.310628	1.056	0.304671	1.383	0.557413
0.451	0.355159	1.005	0.181579	1.367	0.480171
0.521	0.579293	1.069	0.492281	0.948	0.323525
0.45	0.327522	0.852	0.831089	1.027	0.216541
0.414	0.485072	0.785	0.651651	1.371	0.530745
0.384	0.90696	0.853	0.631346	1.011	0.345418
0.512	0.604437	1.016	1.149272	0.732	1.191836
0.451	0.809654	1.107	2.28466	0.87	1.361035
0.386	1.389225	0.979	2.263987	0.881	1.303525
		0.896	2.843293	0.936	1.135713

Endurance

	Bnip3	Rev-erb-α
Mean	0.4176	0.661
Std. Deviation	0.0706	0.3904
Std. Error of Mean	0.01664	0.09201
**Shapiro-Wilk test**		
W	0.9325	0.9357
P value	0.2147	0.2448
Passed normality test (alpha=0.05)?	Yes	Yes
P value summary	ns	ns
**Pearson r**		
r	-0.4004	
95% confidence interval	-0.7307 to 0.08179	
R squared	0.1603	
**P value**		
P (two-tailed)	0.0997	
P value summary	ns	
Significant? (alpha = 0.05)	No	

Interval

	Bnip3	Rev-erb-α
Mean	0.9463	0.8766
Std. Deviation	0.1396	0.8024
Std. Error of Mean	0.03202	0.1841
**Shapiro-Wilk test**		
W	0.8953	0.8166
P value	0.04	0.002
Passed normality test (alpha=0.05)?	No	No
P value summary	*	**
**Spearman r**		
r	-0.2333	
95% confidence interval	-0.6305 to 0.2606	
**P value**		
P (two-tailed)	0.3364	
P value summary	ns	
Exact or approximate P value?	Approximate	
Significant? (alpha = 0.05)	No	

Strength

	Bnip3	Rev-erb-α
Mean	1.032	0.6005
Std. Deviation	0.1903	0.4056
Std. Error of Mean	0.04365	0.09305
**Shapiro-Wilk test**		
W	0.9365	0.8629
P value	0.228	0.011
Passed normality test (alpha=0.05)?	Yes	No
P value summary	ns	*
**Spearman r**		
r	-0.2386	
95% confidence interval	-0.6338 to 0.2554	
**P value**		
P (two-tailed)	0.3253	
P value summary	ns	
Exact or approximate P value?	Approximate	
Significant? (alpha = 0.05)	No	

Figure 6 - Gastrocnemius

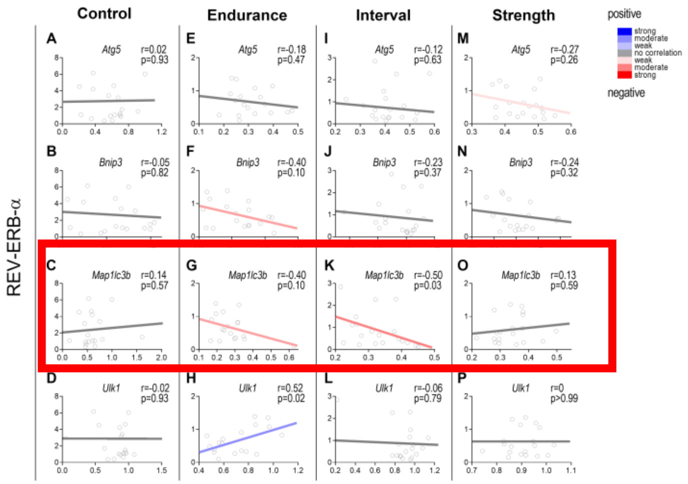

Control	
**Map1lc3b**	**Rev-erb-α**
1	6,038635
0,529	6,156449
0,752	3,403044
0,584	4,626273
0,335	4,518728
0,463	0,353675
0,806	1,232309
0,61	1,082445
0,6	4,212334
0,533	0,947484
0,654	1,0407
1,607	1,509728
0,658	0,408426
0,144	1,093662
0,445	3,147474
0,515	1,726931
0,478	0,641692
0,474	0,912632

	Map1lc3b	Rev-erb-α
Mean	0,6215	2,392
Std. Deviation	0,3074	1,957
Std. Error of Mean	0,07245	0,4613
**Shapiro-Wilk test**		
W	0,8154	0,8467
P value	0,0025	0,0075
Passed normality test (alpha=0.05)?	No	No
P value summary	**	**
**Spearman r**		
r	0,1434	
95% confidence interval	-0,3597 to 0,5820	
**P value**		
P (two-tailed)	0,5701	
P value summary	ns	
Exact or approximate P value?	Approximate	
Significant? (alpha = 0.05)	No	

Figure 6 - Gastrocnemius

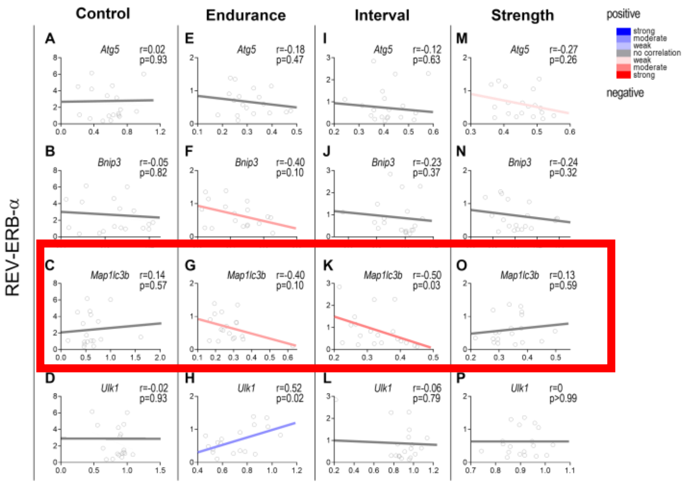

Endurance		Interval		Strength	
**Map1lc3b**	**Rev-erb-α**	**Map1lc3b**	**Rev-erb-α**	**Map1lc3b**	**Rev-erb-α**
0.234	1.141376	0.251	1.107878	0.342	0.642064
0.195	1.079966	0.262	1.480256	0.38	1.054293
0.315	0.827383	0.378	0.817224	0.287	0.508819
0.322	1.350847	0.272	0.215832	0.296	0.314392
0.26	0.705071	0.418	0.31058	0.378	0.639348
0.348	0.397243	0.402	0.380436	0.282	0.215245
0.215	0.2512	0.451	0.134215	0.296	0.188228
0.628	0.107043	0.225	0.310679	0.357	0.152135
0.166	0.269395	0.321	0.265282	0.45	0.249416
0.353	0.310628	0.343	0.304671	0.497	0.557413
0.304	0.355159	0.443	0.181579	0.345	0.480171
0.241	0.579293	0.391	0.492281	0.279	0.323525
0.296	0.327522	0.31	0.831089	0.382	0.216541
0.276	0.485072	0.332	0.651651	0.3	0.530745
0.17	0.90696	0.279	0.631346	0.216	0.345418
0.231	0.604437	0.265	1.149272	0.279	1.191836
0.316	0.809654	0.383	2.28466	0.336	1.361035
0.194	1.389225	0.321	2.263987	0.376	1.303525
		0.216	2.843293	0.38	1.135713

Endurance

Mean	0.2813	0.661
Std. Deviation	0.1048	0.3904
Std. Error of Mean	0.02469	0.09201
**Shapiro-Wilk test**		
W	0.7955	0.9357
P value	0.0013	0.2448
Passed normality test (alpha=0.05)?	No	Yes
P value summary	Ns	ns
**Pearson r**		
r	-0.4015	
95% confidence interval	-0.7313 to 0.08039	
R squared	0.1612	
**P value**		
P (two-tailed)	0.0986	
P value summary	ns	
Significant? (alpha = 0.05)	No	

Interval

Mean	0.3296	0.8766
Std. Deviation	0.07264	0.8024
Std. Error of Mean	0.01666	0.1841
**Shapiro-Wilk test**		
W	0.9565	0.8166
P value	0.5055	0.002
Passed normality test (alpha=0.05)?	Yes	No
P value summary	ns	**
**Spearman r**		
r	-0.5011	
95% confidence interval	-0.7838 to -0.04626	
**P value**		
P (two-tailed)	0.0288	
P value summary	*	
Exact or approximate P value?	Approximate	
Significant? (alpha = 0.05)	Yes	

Strength

Mean	0.3399	0.6005
Std. Deviation	0.06635	0.4056
Std. Error of Mean	0.01522	0.09305
**Shapiro-Wilk test**		
W	0.9463	0.8629
P value	0.3417	0.011
Passed normality test (alpha=0.05)?	Yes	No
P value summary	ns	*
**Spearman r**		
r	0.1309	
95% confidence interval	-0.3565 to 0.5622	
**P value**		
P (two-tailed)	0.5933	
P value summary	ns	
Exact or approximate P value?	Approximate	
Significant? (alpha = 0.05)	No	

Figure 6 - Gastrocnemius

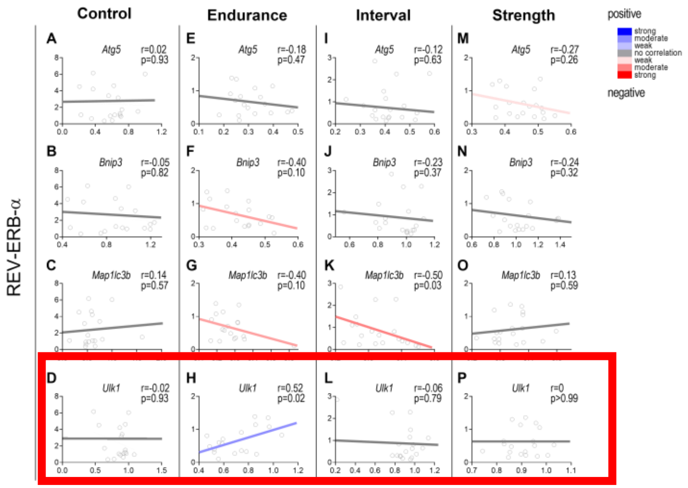

	Control	
	**Map1lc3b**	**Rev-erb-α**
	1	6,038635
	0,472	6,156449
	0,9	3,403044
	0,951	4,626273
	0,552	4,518728
	0,686	0,353675
	0,95	1,232309
	0,925	1,082445
	0,867	4,212334
	1,086	0,947484
	0,854	1,0407
	0,952	1,509728
	0,781	0,408426
	0,966	1,093662
	0,894	3,147474
	0,636	1,726931
	0,92	0,641692
	0,878	0,912632
Mean	0,8483	2,392
Std. Deviation	0,1622	1,957
Std. Error of Mean	0,03824	0,4613
**Shapiro-Wilk test**		
W	0,8853	0,8467
P value	0,0321	0,0075
Passed normality test (alpha=0.05)?	No	No
P value summary	*	**
**Spearman r**		
r	-0,02167	
95% confidence interval	-0,4950 to 0,4616	
**P value**		
P (two-tailed)	0,932	
P value summary	ns	
Exact or approximate P value?	Approximate	
Significant? (alpha = 0.05)	No	

Figure 6 - Gastrocnemius

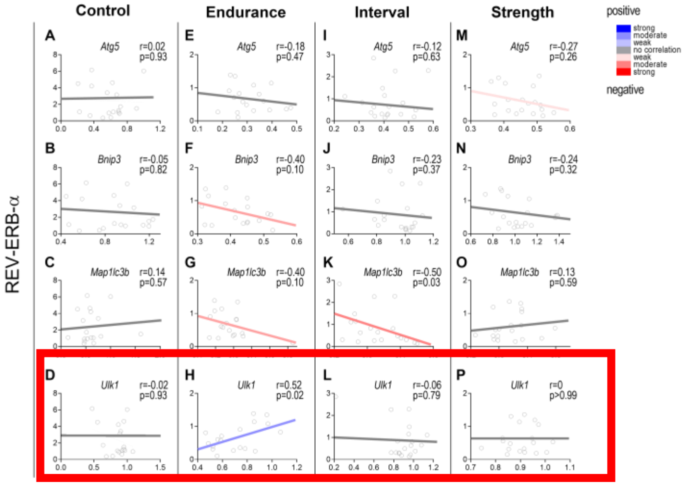

Endurance		Interval		Strength	
**Ulk1**	**Rev-erb-α**	**Ulk1**	**Rev-erb-α**	**Ulk1**	**Rev-erb-α**
0.856	1.141376	1.136	1.107878	0.828	0.642064
0.967	1.079966	0.957	1.480256	0.973	1.054293
1.068	0.827383	0.986	0.817224	0.953	0.508819
0.958	1.350847	0.969	0.215832	0.853	0.314392
0.593	0.705071	0.775	0.31058	0.947	0.639348
0.524	0.397243	1.017	0.380436	1.006	0.215245
0.864	0.2512	0.957	0.134215	0.964	0.188228
0.558	0.107043	0.823	0.310679	0.847	0.152135
0.689	0.269395	0.857	0.265282	0.898	0.249416
0.523	0.310628	0.853	0.304671	0.83	0.557413
0.743	0.355159	0.878	0.181579	0.859	0.480171
0.587	0.579293	0.911	0.492281	0.915	0.323525
0.783	0.327522	0.839	0.831089	0.917	0.216541
0.487	0.485072	1.078	0.651651	1.031	0.530745
0.743	0.90696	0.973	0.631346	0.743	0.345418
0.562	0.604437	0.95	1.149272	0.958	1.191836
0.465	0.809654	0.953	2.28466	0.916	1.361035
0.851	1.389225	0.774	2.263987	0.862	1.303525
		0.216	2.843293	0.896	1.135713

Endurance

	Ulk1	Rev-erb-α
Mean	0.7123	0.661
Std. Deviation	0.185	0.3904
Std. Error of Mean	0.0436	0.09201
**Shapiro-Wilk test**		
W	0.9347	0.9357
P value	0.2345	0.2448
Passed normality test (alpha=0.05)?	Yes	Yes
P value summary	ns	ns
**Pearson r**		
r	0.5253	
95% confidence interval	0.07737 to 0.7967	
R squared	0.2759	
**P value**		
P (two-tailed)	0.0252	
P value summary	*	
Significant? (alpha = 0.05)	Yes	

Interval

	Ulk1	Rev-erb-α
Mean	0.8896	0.8766
Std. Deviation	0.1889	0.8024
Std. Error of Mean	0.04333	0.1841
**Shapiro-Wilk test**		
W	0.7378	0.8166
P value	0.0002	0.002
Passed normality test (alpha=0.05)?	No	No
P value summary	***	**
**Spearman r**		
r	-0.06406	
95% confidence interval	-0.5144 to 0.4139	
**P value**		
P (two-tailed)	0.7944	
P value summary	ns	
Exact or approximate P value?	Approximate	
Significant? (alpha = 0.05)	No	

Strength

	Ulk1	Rev-erb-α
Mean	0.9051	0.6005
Std. Deviation	0.07047	0.4056
Std. Error of Mean	0.01617	0.09305
**Shapiro-Wilk test**		
W	0.9778	0.8629
P value	0.914	0.011
Passed normality test (alpha=0.05)?	Yes	No
P value summary	ns	*
**Spearman r**		
r	0	
95% confidence interval	-0.4656 to 0.4656	
**P value**		
P (two-tailed)	>0.9999	
P value summary	ns	
Exact or approximate P value?	Approximate	
Significant? (alpha = 0.05)	No	
